# Transcriptome and Proteome Analysis Revealed Key Pathways Regulating Final Stage of Oocyte Maturation of the Turkey (*Meleagris gallopavo*)

**DOI:** 10.3390/ijms221910589

**Published:** 2021-09-30

**Authors:** Mariola Słowińska, Łukasz Paukszto, Laura Pardyak, Jan P. Jastrzębski, Ewa Liszewska, Joanna Wiśniewska, Krzysztof Kozłowski, Jan Jankowski, Barbara Bilińska, Andrzej Ciereszko

**Affiliations:** 1 Department of Gamete and Embryo Biology, Institute of Animal Reproduction and Food Research, Polish Academy of Sciences in Olsztyn, 10-748 Olsztyn, Poland; e.liszewska@pan.olsztyn.pl (E.L.); a.ciereszko@pan.olsztyn.pl (A.C.); 2 Department of Plant Physiology, Genetics and Biotechnology, Faculty of Biology and Biotechnology, University of Warmia and Mazury in Olsztyn, 10-719 Olsztyn, Poland; lukasz.paukszto@uwm.edu.pl (Ł.P.); bioinformatyka@gmail.com (J.P.J.); 3 Center of Experimental and Innovative Medicine, University of Agriculture in Krakow, 30-248 Kraków, Poland; laura.pardyak@urk.edu.pl; 4 Department of Biological Function of Food, Institute of Animal Reproduction and Food Research, Polish Academy of Sciences in Olsztyn, 10-748 Olsztyn, Poland; j.bukowska@pan.olsztyn.pl; 5 Department of Poultry Science, Faculty of Animal Bioengineering, University of Warmia and Mazury in Olsztyn, 10-719 Olsztyn, Poland; kristof@uwm.edu.pl (K.K.); janj@uwm.edu.pl (J.J.); 6 Department of Endocrinology, Institute of Zoology, Jagiellonian University, 30-387 Kraków, Poland; barbara.bilinska@uj.edu.pl

**Keywords:** oocyte, zona pellucida glycoproteins, ubiquitination, estrogen–receptor signaling, G protein-coupled estrogen receptor, oxidative phosphorylation, sirtuins, intercellular interactions, oxidative stress response, turkey

## Abstract

In birds, the zona pellucida (ZP) matrix that surrounds the ovulated oocyte—called the inner perivitelline layer—is involved in sperm–zona interaction and successful fertilization. To identify the important genes and proteins connected with the final step of egg development, next-generation sequencing and two-dimensional electrophoresis, combined with mass spectrometry, were used for the analysis of mature oocytes at the F1 developmental stage. A total of 8161 genes and 228 proteins were annotated. Six subfamilies of genes, with codes ZP, *ZP1–4*, *ZPD*, and *ZPAX*, were identified, with the dominant expression of *ZPD*. The main expression site for ZP1 was the liver; however, granulosa cells may also participate in local ZP1 secretion. A ubiquitination system was identified in mature oocytes, where ZP1 was found to be the main ubiquitinated protein. Analysis of transcripts classified in estrogen receptor (ESR) signaling indicated the presence of *ESR1* and *ESR2*, as well as a set of estrogen-dependent genes involved in both genomic and nongenomic mechanisms for the regulation of gene expression by estrogen. Oxidative phosphorylation was found to be a possible source of adenosine triphosphate, and the nuclear factor erythroid 2-related factor 2 signaling pathway could be involved in the response against oxidative stress. Oocyte–granulosa cell communication by tight, adherens, and gap junctions seems to be essential for the final step of oocyte maturation.

## 1. Introduction

The zona pellucida (ZP) matrix that surrounds the ovulated oocyte—called the inner perivitelline layer (IPVL)—is involved in sperm binding during the initial process of fertilization in birds [1]. Fertilization begins from sperm penetration of the IPVL, mostly in the germinal disc region, and it has been suggested that ZP glycoproteins and their turnover, including ZP1 ubiquitination, play a crucial role in sperm–zona interaction [2]. The most dominant ZP glycoproteins of the bird’s mature egg coat (ZP1, ZP3, and ZPD) are synthesized by the liver and in the granulosa cells (GCs) surrounding preovulatory-growing oocytes [3,4,5,6,7,8,9]. To date, the aforementioned glycoproteins have been described in detail for Japanese quail egg development [10]. Based on general phylogenetic analysis of vertebrates, chicken ZP glycoprotein genes have been classified into six subfamilies: ZP1, ZP2/ZPA, ZP3/ZPC, ZP4/ZPB, ZPD, and ZPAX [11]; however, there is lack of comprehensive analyses of ZP genes for other bird species. 

The GC layer surrounding the bird oocyte plays a key role in the regulation of follicle secretion [12] and in the formation of the IPVL in yellow follicles up to egg ovulation [13]. GCs start to develop in the primary follicles at the initial phase of oogenesis, and are detached from the mature egg during ovulation, when the egg is released from the follicular capsule tissue [1]. In contrast to mammals, knowledge regarding the specific function of bird GCs in the last step of egg maturation is very limited, and the mechanisms of intercellular interactions of GCs—a central element of molecular transport to mammalian oocytes [14,15]—is still unknown for birds. Using microarray analyses, only a few genes thought to be involved in oocyte maturation and early embryo development in chicken hens have been identified [13]. New comprehensive technologies, such as next-generation sequencing (NGS) and proteomics tools, may significantly expand our existing knowledge surrounding the specific function of GCs in the final step of preovulatory egg maturation, such as the formation of the IPVL environment for successful fertilization. In the future, the complete set of RNA transcripts from mature egg coats could be useful for the identification of complementary sites for sperm proteins, as has already been suggested for cysteine-rich venom protein in turkey spermatozoa [16].

NGS has recently been used to research the ovary and oviduct during the process of chicken egg formation [17,18,19], chicken and duck eggshell and albumen formation [20,21], and to research higher- and lower-laying duck and chicken ovaries [22,23] and chicken cuticle deposition, in connection to naturally good and poor cuticle quality [24]. Candidate microarray analysis of the germinal disc region of chicken oocytes has revealed genes potentially involved in “laying” and “meat” line hen fertility [25], as well as a few genes involved in oocyte maturation and early embryo development in chicken hens [13]. Transcriptomic and proteomic analyses have been used to determine the mechanisms involved in chicken follicle selection [26,27,28], and proteomic analysis has been widely used for the analysis of the laid chicken egg vitelline membrane [29,30], and egg yolk [31] and egg white proteome [32]. However, there is a lack of similarly comprehensive studies for the turkey hen reproductive tract. Therefore, in the present study, NGS and two-dimensional electrophoresis (2DE) combined with mass spectrometry were used to analyze, for the first time, the transcriptome and proteome of turkey hen mature oocytes at the F1 developmental stage. The aim of this study was to determine key genes and/or proteins of the mature turkey oocyte and assign them to the molecular mechanisms and pathways associated with the final step of egg maturation (i.e., preparing the egg for successful fertilization). Overall, the pattern of genes coding ZP, *ZP1-4*, *ZPD*, and *ZPAX* were identified for the mature egg. IPA analysis revealed that many of the identified genes/proteins are involved in key steps of protein synthesis, through eukaryotic initiation factor (eIF2), eIF4 and ribosomal protein S6 kinase beta-1 (p70S6K) signaling pathways, degradation by ubiquitination, and the action of female sex hormones by estrogen receptor signaling. Oxidative phosphorylation was found to be a possible source of ATP, and the nuclear factor erythroid 2-related factor 2 (NRF2) signaling pathway could be involved in the response against oxidative stress. Finally, oocyte-granulosa cell communication by adherens, tight, and gap junctions seems to be essential for the final step of oocyte maturation.

## 2. Results

### 2.1. Sequencing Results

RNA sequencing (RNA-Seq) generated 663,192,008 raw paired-end reads. During the filtrating procedure, 654,770,024 reads indicated high-quality rating and low adapter levels (Table 1). On average, 80.88% of reads were uniquely mapped to the turkey genome. According to known gene structures located in the turkey genome, the distribution of mapped reads was as follows: 44.30% were aligned to intergenic regions, 6.74% to intronic regions, 5.92% to untranslated regions (UTRs), and 43.04% to coding regions (Table 1). The StringTie and ballgown approaches confirmed the expression of 40,384 transcripts, located within 18,510 transcriptionally active regions (TARs). According to the expression level, 4961 TARs were classified in the group of low expression, with overall fragments per kilo base per million mapped reads (FPKM) values smaller than 1. The largest number (12,984 TARs) was described as having medium expression (FPKM in the range from 1–100). The high abundance of expression levels was assigned to 565 TARs (FPKM > 100).

### 2.2. Transcriptome of Inner Perivitelline Layer: Functional Annotation and Pathway Analysis

Among the middle- and high-expressed 13,549 TARs identified in the IPVL, 8161 genes were annotated as protein coding genes, 213 as known noncoding transcripts, and 5175 as unannotated regulatory RNA sequences (see Supplementary Materials, Table S1). Transcript quantification by FPKM value showed that cytochrome c oxidase subunit 1 (*COX1*), copper metabolism domain-containing 4 (*COMMD4*), and NPC intracellular cholesterol transporter 2 (*NPC2*) were the most expressed transcripts in turkey IPVL.

The transcript analysis by IPA showed canonical pathway, molecular and cellular functions, and networks which were significant in terms of *p*-values and/or a score (Table 2, and Supplementary Materials, Table S2). The top canonical pathways of the IPVL included the protein ubiquitination pathway, eiF2, and regulation of eIF4 and p70S6K signaling, and sirtuin and estrogen receptor signaling (Table 2). The top molecular and cellular functions and the most significant physiological functions are presented in Table 2.

### 2.3. Proteome of Inner Perivitelline Layer: Functional Annotation and Pathway Analysis

A total of 458 spots were detected, of which 384 spots were successfully identified by MALDI TOF/TOF and assigned to 228 proteins (Supplementary Materials, Figure S1 and Table S3). Some of the IPVL proteins were identified in more than one protein spot, likely representing protein proteoformes differing in isoelectric point and/or molecular weight; for example, ZP1 was identified in 25 spots, ZP3 was identified in 22 spots, and elongation factor 1-alpha 1-like (EEF1A1) and a cholesterol side-chain cleavage enzyme, mitochondrial (CYP11A1), were identified in 5 spots each. The results of spot identification after 2DE are reported in the Supplementary Materials, Table S3. Spot quantification by spot area showed that ZP3, thioredoxin (TXN), and ZP1 were the most dominant proteins in turkey IPVL.

Proteomic profiling of the identified protein data set by IPA demonstrated that the top canonical pathways included oxidative phosphorylation, mitochondrial dysfunction, an NRF2-mediated oxidative stress response, the remodeling of epithelial adherens junctions, and sirtuin signaling pathway (Table 2, and Supplementary Materials, Table S2). Molecular and cellular functions and the most significant physiological functions are presented in Table 2.

### 2.4. Phylogenetic Analysis of Bird ZP Glycoproteins

Phylogenetic analysis of the ZP gene family, which contained 243 sequences of bird and 18 sequences of outgroup taxa, yielded a well-resolved tree defining the six clades corresponding to ZP1–ZP4, ZPD, and ZPAX (Supplementary Materials, Figure S2). In the tree, particular ZP clades compromised several subclades, which received high node credibility in our analyses (all posterior probability values > 0.5). Detailed analysis of the subclades indicated that turkey (Mg) was closely related to and clustered into one subclade with chicken (Gg) (ZP1–ZP4, ZPD, and ZPAX clades), helmeted guineafowl (Nm), Japanese quail (Nm) (ZP1, ZP2, ZP4, ZPD, and ZPAX clades), and Indian peafowl (Pc) (ZP2–ZP4, ZPD, and ZPAX clades). Mammal (Hs), reptile (Ps), amphibian (Xt), and fish (Dr) ZP glycoproteins were clustered into a separate clade; however, a long-stem branch preceding birds and reptiles indicated that avians and reptiles are closely related paralogs. Clades ZP2 and ZPAX, as well as ZP1 and ZP4, were closely related, originating from the same branch.

### 2.5. Identification of Ubiquitinated Proteins and Immunohistochemical Detection of Ubiquitin in the IPVL and the GC layer

**Figure 1:**
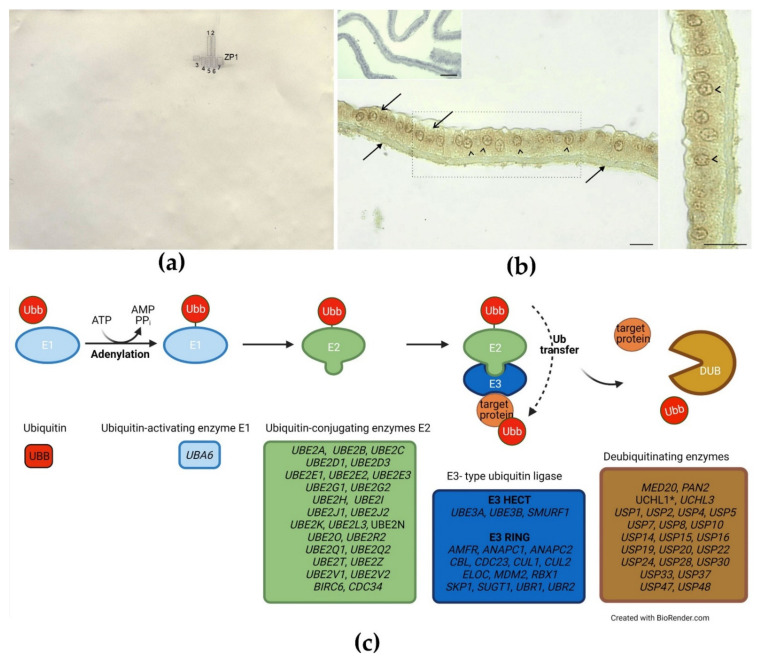
Ubiquitination of the inner perivitelline layer (IPVL) in mature oocyte: (**a**) Cross-reactivity between anti-ubiquitin antibodies and IPVL proteins after 2DE were analyzed by Western blot, and detected spots were identified as ZP1 proteins (the results of spot identification are presented in the Supplementary Materials, Table S4); (**b**) Immunohistochemical detection of ubiquitin in the IPVL and the granulosa cell (GC) layer. Counterstaining was performed with Mayer’s haematoxylin. Scale bars = 10 µm. Frame indicates the location of the higher magnification view. Weak and moderate signals for ubiquitin are localized to the IPVL (arrows) and cytoplasm of GCs (open arrows). Note the strong linear staining pattern in the perinuclear cytoplasm of GCs (arrowheads). No immunopositive signal was observed for ubiquitin in the IPVL and the GC layer when the primary antibody was omitted (upper insert); (**c**) Transcripts/proteins identified in the IPVL participating in the protein ubiquitination pathway and deubiquitinating enzymes (DUB). Presented using the BioRender software. Description of gene/protein symbols are presented in the Supplementary Materials, Tables S1 and S3.

Western blot analysis shown that polyclonal anti-ubiquitin antibodies cross-reacted with ZP1, which was identified directly from nitrocellulose (NC) membrane (Figure 1a, and Supplementary Materials, Table S4). Using immunohistochemistry, positive signals of ubiquitin both in the IPVL and the GC layer were observed (Figure 1b). A weak diffuse staining pattern was observed in the IPVL (Figure 1b). In the GC layer, the staining intensity for ubiquitin was evidently stronger when compared to the IPVL. A diffuse staining pattern of weak-to-moderate intensity was observed in the cell cytoplasm. Moreover, as shown in the higher magnification view, GCs displayed a strong positive signal located in the perinuclear cytoplasm. No immunopositive ubiquitin signal was observed in the control sections, in which the primary antibody was omitted and replaced with pre-immune serum (top insert in Figure 1b).

### 2.6. Immunohistochemical Detection of Cell Junction Proteins in GCs Surrounding the IPVL

**Figure 2:**
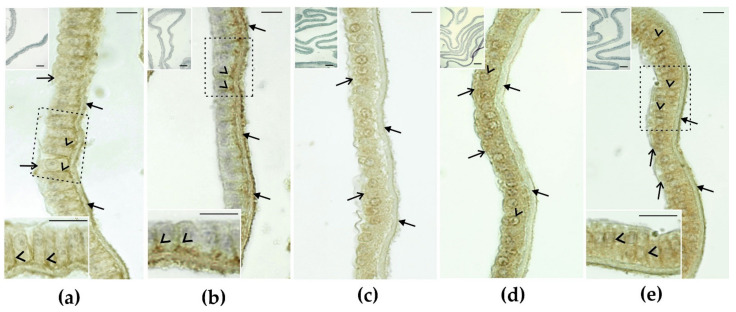
Immunohistochemical staining for cell junction proteins in the inner perivitelline layer (IPVL) in mature oocyte. Adherens junction proteins: (**a**) N-cadherin and (**b**) β-catenin; Tight junction proteins: (**c**) occludin and (**d**) tight junction protein-1; and gap junction protein: (**e**) connexin 43. All sections were counterstained with Mayer’s haematoxylin. Bars = 10 µm. Frames indicate the location of the higher magnification view. (**a**) Positive diffuse staining for *N*-cadherin in the IPVL (arrows) and the granulosa cell (GC) layer (open arrows) is visible. Note weak-to-moderate linear staining in the lateral plasma membranes between neighboring cells of the GC layer (arrowheads); (**b**) A very strong signal of β-catenin is localized to the IPVL (arrows), while that of moderate intensity is observed in the lateral plasma membranes of the GC layer (arrowheads). For details, see the higher magnification panel. (**c**) Positive staining for occludin is restricted to the apical compartment of the GC layer (open arrows), whereas no positive signal is observed in the IPVL (arrows); (**d**) The strong tight junction protein-1 signal is either dispersed at the cytoplasm of the GC layer (open arrows) or visible in the linear staining pattern of the GC perinuclear cytoplasm (arrowheads). No positive signal for the tight junction protein-1 is localized to the IPVL; (**e**) Positive staining for connexin 43 is visible in the IPVL (arrows) and the GC layer (open arrows). Connexin 43 is distributed in a linear array between granulosa cells (arrowheads). For details, see the higher magnification panel. No immunopositive staining for, *N*-cadherin, β-catenin, occludin, tight junction protein-1 and connexin 43 was observed when the primary antibodies were omitted (**a**–**e**, inserts in upper left corners of microphotographs).

As revealed by immunohistochemistry, positive signals of junctional proteins were present in the IPVL and the GCs of turkeys (Figure 2). Adherens junction protein—*N*-cadherin (CDH2) and β-catenin (CTNNB)—signals were observed in the IPVL and the GC layer (Figure 2a,b). CDH2 was localized similarly to that of CTNNB (Figure 2a); however, in contrast to CTNNB, a dispersed signal of CDH2 in the GCs cytoplasm was noted (Figure 2a, see higher magnification). A very strong signal of CTNNB was observed in the IPVL. In the GC layer, a CTNNB signal was observed along the lateral plasma membranes of adjacent GCs (Figure 2b, see higher magnification).

Tight junction protein—occludin (OCLN) and zonula occludens-1 (also known as tight junction protein-1, TJP1)—signals were observed only in cytoplasm of GCs. Weak-to-moderate immunostaining for OCLN was observed in the apical cytoplasm of the GC layer (Figure 2c). By contrast, the IPVL was immunonegative. The strong TJP1 signal was either dispersed at the cytoplasm of the GC layer or visible as the linear staining pattern in the perinuclear cell cytoplasm. No immunoreaction was found in the IPVL (Figure 2d).

The gap junction protein—connexin 43 (Cx43)—signal appeared in both compartments: the IPVL and the cytoplasm of the GC layer (Figure 2e). Closer examination of the GC layer revealed positive staining between adjacent cells, as shown at higher magnification (Figure 2e).

No signals were detected for CDH2, CTNNB, OCLN, TJP1, and Cx43, when primary antibodies were omitted and replaced by normal horse or goat serum (Figure 2a–e, inserts in upper left corner of microphotographs).

### 2.7. Validation of Selected Genes by Quantitative Real-Time Reverse Transcriptase-Polymerase Chain Reaction (qRT-PCR)

**Figure 3:**
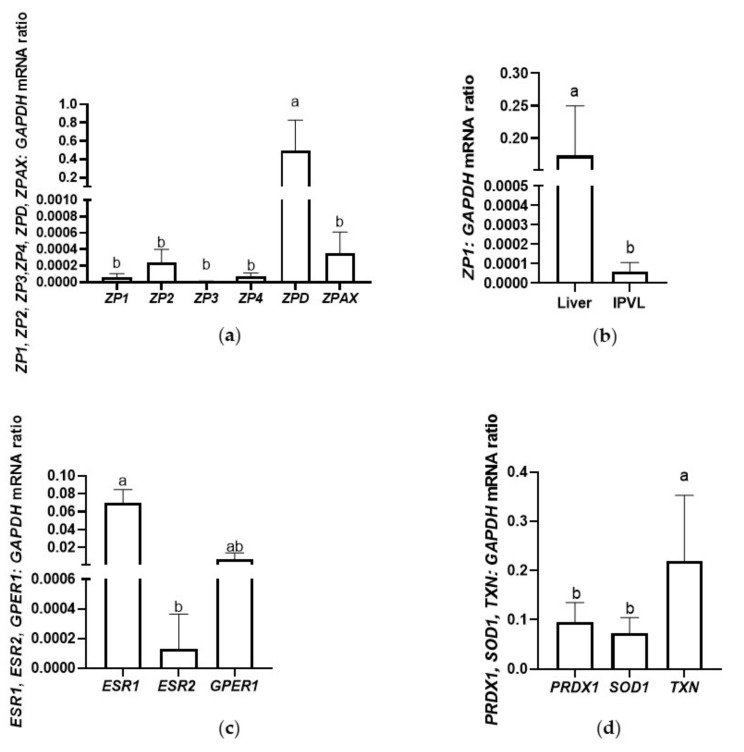
Real-time validation of selected genes identified in the inner perivitelline layer by RNA-Seq. Validation was performed using the same RNA samples as for NGS. Data are expressed as mean ± SD. (**a**) Expression of six zona pellucida glycoprotein genes (*ZP1*, *ZP2*, *ZP3*, *ZP4*, *ZPD*, and *ZPAX*); (**b**) Expression of *ZP1* in the liver and the IPVL; (**c**) Expression of three genes involved in estrogen receptor signaling (*ESR1*, *ESR2*, and *GPER1*); and (**d**) Expression of three genes involved in the NRF2-mediated oxidative stress response (*SOD1*, *PRDX1*, and *TXN*). *GAPDH*: glyceraldehyde-3-phosphate dehydrogenase. Different letters indicate statistical significance at *P* ≤ 0.05.

**Figure 4:**
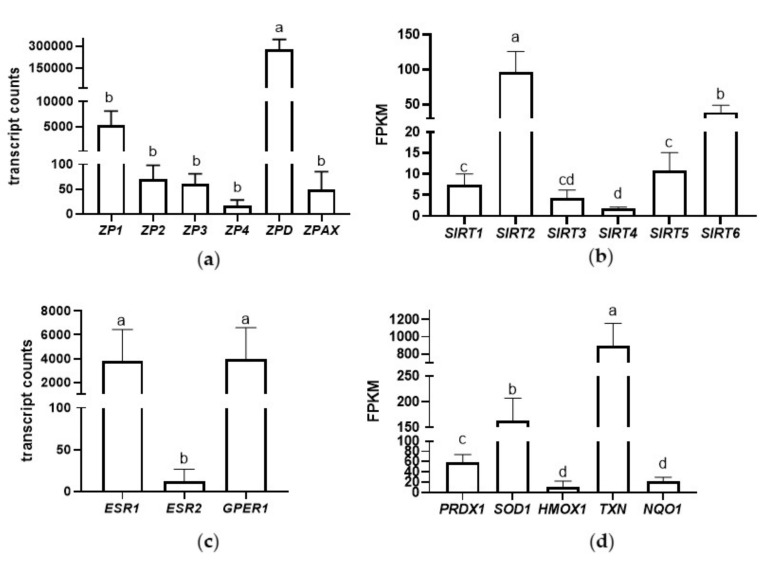
Transcripts identified in the inner perivitelline layer by RNA-Seq. Data are expressed as the mean ± SD. (**a**) Expression of six zona pellucida glycoprotein transcripts (*ZP1*, *ZP2*, *ZP3*, *ZP4*, *ZPD*, and *ZPAX*); (**b**) Expression of six sirtuin transcripts (*SIRT1–6*); (**c**) Expression of three transcripts involved in estrogen receptor signaling (*ESR1*, *ESR2*, and *GPER1*); and (**d**) Expression of five transcripts involved in the NRF2-mediated oxidative stress response (*PRDX1*, *SOD1*, *TXN*, heme oxygenase (*HMOX1*), and NAD(P)H quinone dehydrogenase 1 (*NQO1*)). Different letters indicate statistical significance at *P* ≤ 0.05.

To validate the NGS results, six zona pellucida glycoprotein genes (*ZP1*, *ZP2*, *ZP3*, *ZP4*, *ZPD*, *ZPAX*), three genes involved in estrogen receptor signaling (estrogen receptors (*ESR1*, *ESR2*) and G protein-coupled estrogen receptor 1 (*GPER1*) genes), and three genes involved in the NRF2-mediated oxidative stress response (peroxiredoxin 1 (*PRDX1*), superoxide dismutase 1 (*SOD1*), and *TXN*) were selected for qRT-PCR analysis (Figure 3). We confirmed that *ZPD* had the highest expression level of the ZP glycoproteins in the mature egg coats (Figure 3a). Among the genes involved in estrogen receptor signaling, *ESR1* was confirmed to have the highest expression level (Figure 3c). Among the three most important genes involved in the oxidative stress response, we confirmed *TXN* as the highest expressed in the mature egg coats (Figure 3d). The NGS results of ZP glycoprotein transcripts, transcripts involved in estrogen receptor signaling, and NRF2-mediated oxidative stress response are presented in Figure 4.

## 3. Discussion

This study is the first on birds to provide comprehensive information for the transcriptomic and proteomic characterization of the IPVL in mature hen oocytes. The protein ubiquitination pathway and oxidative phosphorylation were found to be the most significant pathways of turkey IPLV genome and proteome, respectively. A strong overlap between IPA canonical pathways imputed from transcriptomic and proteomic data was observed. Six subfamilies of ZP genes—*ZP1-4*, *ZPD*, and *ZPAX*—were identified and, among them, *ZPD* was characterized by the highest expression level. Phylogenetic analysis of bird ZP glycoproteins indicated that turkey ZP glycoproteins are conserved among galliforms. Immunohistochemical analysis revealed the presence of ubiquitin in the IPVL and the GC layer, where the main ubiquitinated protein was identified as ZP1. The proteins of adherens, tight, and gap junctions were localized in the mature egg coats. The expression of ZP glycoprotein genes, genes involved in estrogen receptor signaling, and those involved in the NRF2-mediated oxidative stress response were validated using qRT-PCR.

### 3.1. ZP Glycoproteins

In birds, the vitelline layer surrounding the egg is principally composed of hepatic ZP1, follicular ZP3, ZPD, and (in lesser amounts) ZP2 and ZP4 [2,33,34]. ZP1 is synthesized in the liver and is transported to the ovary via blood circulation [3,5]. In contrast, ZP3 is secreted from the GCs surrounding the preovulatory growing oocyte [4,6,7,8,35]. In quail, it has been revealed that ZP1 contributes the largest amount of glycoprotein to the IPVL of F1 follicles, and was detected in F4-sized follicles, whereas ZP3 was detected in small-sized yellow follicles with an abrupt increase during the final 48 h of follicular development [36,37]. Our proteomic analysis confirmed, for the first time, that ZP1 and ZP3 are the most dominant proteins in turkey IPVL obtained from F1-largest yellow follicles. These results are in agreement with the model of IPVL development in birds presented by Rodler et al. [10] for Japanese quail, showing the main participation of ZP1 and ZP3, as well as the minor effect of ZPD in egg coat development from F4 stage up to ovulation.

According to current knowledge, the liver is the main secretion site for ZP1, which is then secreted into the bloodstream and transported to the ovary [3,5]. So far, the expression of *ZP1* has been confirmed only in Japanese quail liver [5]. In our study, we confirmed the high expression of *ZP1* in turkey hen livers (Figure 3b). However, the results of our study challenge knowledge regarding *ZP1* gene expression: we observed, for the first time, the presence of a *ZP1* transcript/gene in a bird IPVL, suggesting that GCs (beside the secretion of other ZP glycoproteins) might be also responsible for ZP1 synthesis. In domestic mammals and humans, both the oocyte and GCs contribute to the formation of the ZP proteins [11,38,39]. It may be assumed that, similarly to mammals, mature bird follicles might also partially participate in local ZP1 secretion. In summary, the results of our study and those of Sasanami et al. [5] are not compatible, and future studies concerning the origin of ZP1 in the IPVL—particularly, the special role of its local synthesis in the IPVL—should be investigated.

Similar to the protein pattern of ZP3, an abrupt increase of *ZP3* mRNA has been observed only during the final 48 h of chicken follicular development [37]. In our study, we confirmed that ZP3 is one of the dominant proteins in the IPVL during the F1 oocyte stage. We also confirmed the presence of *ZP3* transcript/gene in turkey IPVL; however, the relative expression of *ZP3* was lower. It should be noted that a weak link between proteomic and transcriptomic data often exists [40], for several biological reasons. A weak correlation between measured RNA and proteins could be attributed to the presence of weak ribosome binding sites, regulatory proteins, codon usage bias, and the half-life difference between protein and mRNA [40]. Further proteomic and transcriptomic experiments at several time points—especially before ovulation—are necessary to establish the precise link between mRNA and protein expression for ZP3.

In our study, we found low relative expressions for both ZP2 and ZP4 transcripts/genes and a lack of *ZP2* and *ZP4* proteins in the IPVL of largest yellow follicles at F1 stage. In birds, ZP2 and ZP4 are deposited relatively early in the ooplasma membrane of the oocyte, during folliculogenesis, and seem to be attenuated in more mature follicles, as has been demonstrated through the use of immunohistochemical methods in Japanese quail [10]. Further, mRNA for these two ZP proteins was exclusively expressed in oocytes at the small white follicles stage in Japanese quail [33,34]. In the most mature follicles (at F3, F2, and F1 stages), no signal for *ZP2* and *ZP4* was demonstrated [33,34]. This explains the low relative expression of *ZP2* and *ZP4* transcripts/genes and lack of ZP2 and ZP4 proteins observed in our study, as only the largest yellow follicles at F1 stage were used for analysis.

Avian ZPD has been incorporated as the latest (after the sequential incorporation of ZP2 and ZP4, ZP3 and ZP1) into the IPVL [9], and binds loosely to the egg envelope matrix [6]. In situ hybridization studies have demonstrated the occurrence of *ZPD* mRNA in GCs, which were found to be the secretion source of avian ZPD [8,9]. Our study confirmed the presence of a pancreatic secretory granule membrane major glycoprotein GP2 transcript, corresponding to turkey ZPD, in the IPVL of turkey oocyte at F1 stage with a high relative expression. In Japanese quail, the expression level of *ZPD* has been found to increase gradually during the development of the follicle, where the largest follicle at F1 stage was characterized by the highest expression of *ZPD* [8]. In our study, the relative expression of turkey *ZPD* transcripts was the highest among other (i.e., *ZP1–ZP4* and *ZPAX*) transcripts, suggesting its importance in F1 stage oocytes. In the available literature, there is a lack of comprehensive studies describing the expression pattern of all avian ZP genes. Further investigation might reveal the complete ZP protein gene pattern during avian folliculogenesis.

In our study, we confirmed the presence of a *ZPAX* transcript at low levels in the turkey IPVL of mature eggs. So far, the *ZPAX* gene has been found in chicken [41], in which three *ZPAX1–3* genes were observed as a result of tandem duplication. In birds, the *ZPAX* gene, similarly to *ZP2* and *ZP4*, is present at a very low level in the mature egg coats [6,8,29,35]. Beside birds, at least one *ZPAX* gene has been found in the lower vertebrates, such as fish and amphibians, whereas, in most mammals, *ZPAXs* are pseudogenes [11,42]. There is no information regarding the functions of *ZPAX* genes for vertebrates. This has only been suggested, because *ZPAX* has the highest sequence identity to *ZPA* (as also observed in our phylogenetic analysis). Therefore, the ZPAX in species lacking ZPA might have a similar function to mammalian ZPA [43]. Further studies of ZPAX are necessary, in order to unravel its functions in lower vertebrates, with a particular emphasis on poultry.

### 3.2. Ubiquitination

The ubiquitin–proteasome system (UPS) serves important functions in avian fertilization [44]. So far, ZP1 ubiquitination has been confirmed only for Japanese quail egg envelopes, using detection with both anti-ubiquitin and anti-ZP1 antibodies [44]. Our results provided, for the first time, direct evidence of ZP1 ubiquitination in mature turkey egg coats, as only ZP1 was identified by mass spectrometry in spots detected after Western blot analysis with anti-ubiquitin antibodies. Ubiquitinated ZP1 proteins, bound with ZP3 to form the insoluble fiber of the IPVL, are the target protein of the sperm proteasome [2]. Sperm proteasomes have been identified, so far, in chicken and turkey spermatozoa after LC/MS and RNA-Seq analyses, respectively [45,46]. During sperm penetration of the IPVL, the sperm proteasome degrades ubiquitinated ZP1, and the fragment of ZP1, as well as intact ZP3, are released from the IPVL [2,44]. It has been suggested that ZP1 might be ubiquitinated extracellularly during transport in the bloodstream to the IPVL [2,44], and/or when it passes through the gap between the GCs. Our immunohistochemical staining confirmed the presence of ubiquitinated proteins (identified in our study as ZP1), both in the IPVL as well as in the cytoplasm of the GC (especially the perinuclear cytoplasm), indicating the possibility of local ZP1 ubiquitination by GCs, as has been described for ZP proteins during pig oocyte maturation [47,48].

Here, we report, for the first time, the presence of a ubiquitination system existing in the egg coat of mature eggs. We identified 142 transcripts and 15 proteins which may be involved in the local protein ubiquitination pathway in turkey egg coats. Among them, we identified the ubiquitin-activating enzyme E1, the ubiquitin-conjugating enzymes E2− and E3−type ubiquitin ligases (E3 HECT and target E3 protein RING) as well as deubiquitinating enzymes (DUBs), which catalyze the removal of ubiquitin and regulate ubiquitin-mediated pathways (Figure 1c). One DUB of particular interest is ubiquitin C-terminal hydrolase L1 (UCHL1), present in the plasma membrane of oocytes, which is required to block polyspermy in mammals [49]. UCHL1 is essential for proper functioning of UPS [50]. In comparison to mammals, birds exhibit physiological polyspermy during fertilization; only a polyspermic egg can develop normally and, so, the specific function of UCHL1 in birds is unknown at present. In summary, we reported a high number of transcripts involved in the particular steps of ubiquitination in turkey IPVL, suggesting a great intensity of proteolytic events in mature oocytes. According to Ichikawa et al. [2], UPS is pivotal to making holes in the IPVL, and can be a part of the molecular mechanism of the sperm–egg interaction in birds. Further studies should focus on the molecular mechanism of UPS and on indicating whether UPS is involved in polyspermy in birds.

### 3.3. Protein Synthesis

**Figure 5:**
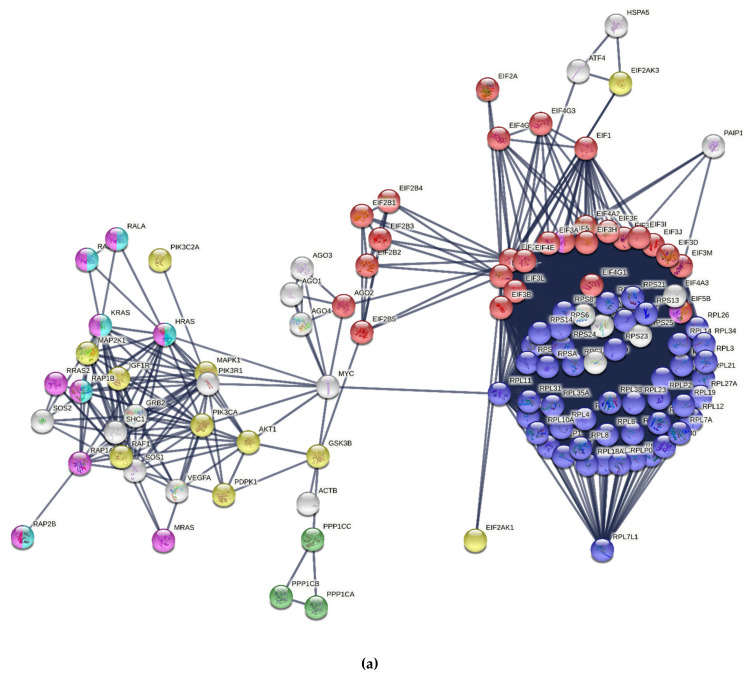

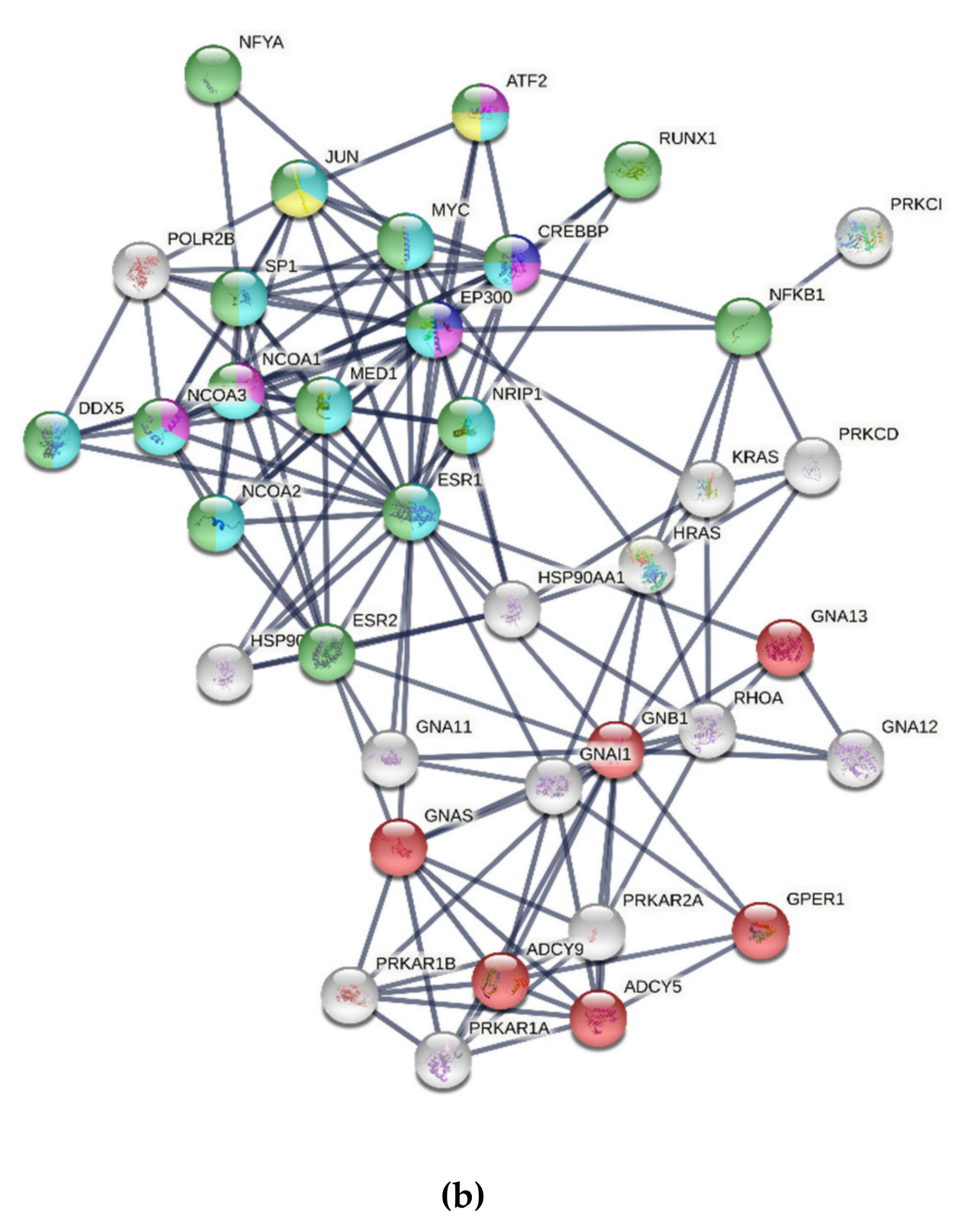
Protein–protein interaction networks for transcripts identified in the inner perivitelline layer, involved in: (**a**) eIF2 signaling, and (**b**) estrogen receptor signaling, according to an analysis by the STRING online database (version 11.0). The line indicates the strength of the data at the highest score (0.9). (**a**) Functional enrichments in eIF2 signaling, red: translation initiation factor activity, dark blue: structural constituent of ribosome, purple: GDP binding, green: protein serine/threonine phosphatase activity, light blue: GTP-binding, and yellow: kinase; (**b**) Functional enrichment in estrogen receptor signaling. Red: adenylate cyclase-activating G protein-coupled receptor signaling, dark blue: regulation of transcription from RNA polymerase II promoter, purple: histone acetyltransferase activity, light blue: DNA-binding transcription factor binding, yellow: AP-1 transcription factor, and green: transcription regulation. Description of gene/protein symbols are presented in the Supplementary Materials, Tables S1 and S3.

**Figure 6:**
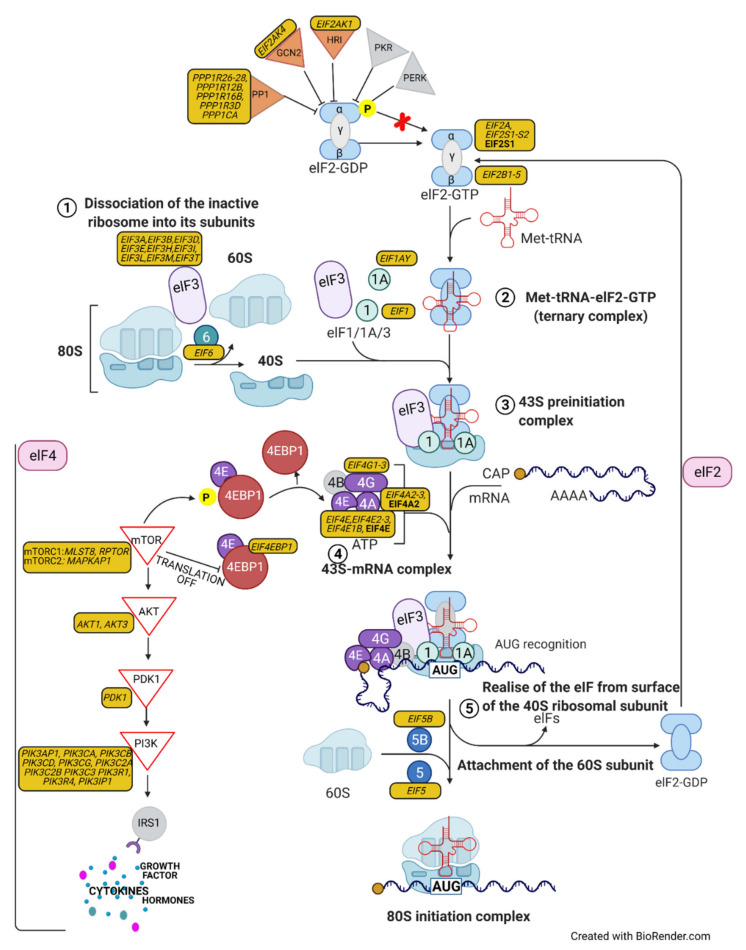
Transcripts/proteins identified in the inner perivitelline layer participating in protein synthesis by eIF2 signaling and eIF4 and p70S6K signaling. The scheme was prepared using the BioRender software. Description of gene/protein symbols are presented in the Supplementary Materials, Tables S1 and S3.

Translational control plays a fundamental role during oogenesis and early embryo development in metazoans [51]. In our study, we indicated that eIF2 signaling and the regulation of eIF4 and p70S6K signaling pathways—both involved in protein synthesis—are the most significant canonical pathways in the transcriptome, as well as in the proteome, of mature egg coats. The eIF2 signaling pathway has recently been identified in chicken follicles after cyclic recruitment, and eIF2 activation reflects the demand for enhanced protein synthesis during follicle development [28]. In bird oogenesis, primary follicles aprox. 1–2 mm in diameter rapidly incorporate the protein-rich yolk and increase in size, up to ~40 mm in diameter, until ovulation, and the area of the egg coat increases several times [1]. Our study indicated that protein synthesis seems to be a crucial process for the transformation of the egg coat during oocyte maturation in birds. Protein synthesis requires cooperation among a large number of polypeptides, including ribosomal proteins, modification enzymes, and ribosome-associated translation factors, which were identified within the eIF2 signaling pathway in our study (Figure 5a). The molecules representing the eIF2 signaling pathway are involved in several steps of initiation translation, such as: (1) dissociation of the inactive ribosome into its subunits (eiF3 and eIF6); (2) formation of the Met-tRNA-eIF2-GTP ternary complex (eIF2); (3) formation of the 43S preinitiation complex (eIF3, eIF1); (4) binding of mRNA with one molecule of ATP (eIF4); and (5) realization of the eIF from the surface of the 40S ribosomal subunit and attachment of the 60S subunit (eIF5) (see Figure 6). In the eIF2 signaling of turkey egg coats, we found transcripts of eIFα kinases participating in the regulation of initiation of translation in mammals—eIF-2-alpha kinase *GCN2* and *HRI*—which are activated by nutrient deprivation and heme deficiency, respectively [52] (Figure 6).

Initiation factors of the eIF4 group and poly(A)-binding protein (PABP) mediate the recruitment of mRNAs to ribosomes, in order to initiate translation. In our study, we identified transcripts and proteins of the eIF4 group, including: (1) eIF4A, an RNA helicase; (2) eIF4E, a cap binding protein; and (3) eIF4G, the central organizing protein that colocalizes eIF4E, eIF4A, eIF3, PABP, and RNA in the 43S initiation complex. We also identified several stimuli, including growth factors and cytokines, that are responsible for the regulation of the eIF4 complex and p70S6K by initiating a phosphorylation cascade involving the sequential activation of kinases, including phosphoinositide 3-kinase (PI3K), protein-serine/threonine kinase (PDK1), nonspecific serine/threonine protein kinase (AKT), and serine/threonine-protein kinase TOR (mTOR) [53], all of which have been identified in our study (Figure 6). The eIF4 signaling pathway has, so far, been identified in mammalian oocytes with regard to its activation [54], defects in maturation [55], and the segregation of genetic maternal during meiosis [56]. Interestingly, the latest function was connected with a key translational regulation factor—eukaryotic translation initiation factor 4E binding protein 1 (EIF4EBP1)—the transcript of which was identified in our study.

### 3.4. Sirtuin Signaling Pathway

Sirtuins (SIRT1–7) are a family of NAD^+^-dependent deacetylases that catalyze the post-translational modifications of proteins. The role of sirtuins has been studied in relation to female reproductive biology, and the expression of *SIRT1*, *SIRT2*, *SIRT3*, and *SIRT6* have been described in mammals in relation to oocyte growth and maturation [57]. In our study, we identified, for the first time, transcripts of *SIRT1–6* in turkey egg coat F1 follicles, as well as sirtuin-interacting molecules (i.e., NADH dehydrogenase and oxidoreductases), which were classified together into the sirtuin signaling pathway. Among the identified sirtuins, *SIRT2* and *SIRT6* were more strongly expressed than the others in the egg coat of mature turkey oocytes (Figure 4b). In mammals, *SIRT2* and *SIRT6* are strongly expressed in the meiotic stage of oocytes, and are involved in the protection of the oocyte against aging [58,59,60,61]. The role of SIRT2 and SIRT6 related to the protection of oocytes against aging seems to be interesting, from our point of view. Downregulation of SIRT2 and SIRT6 causes cellular fragmentation, spindle defects, elevated reactive oxygen species (ROS) accumulation, mitochondrial dysfunction, and the upregulation of autophagy [58,62]. In our study, the increased expression of *SIRT2* and *SIRT6* in the mature egg coat of oocytes just before ovulation suggests their important function in the quality control of bird oocytes before fertilization. 

### 3.5. Estrogen Receptor Signaling

The presence of receptors for progesterone, androgen, and estrogen has been established in bird follicular cells [63]. It was suggested that the abovementioned steroids have key functions in regulating the proliferation and differentiation of follicular cells, follicle maturation, and, finally, in ovulation, through receptor-mediated pathways. In our study, we found estrogen receptor signaling pathways within the most significant pathways of the coats of mature bird egg transcriptome. In vertebrates, two subtypes of the nuclear estrogen receptor (ESR1 and ESR2) encoded by separate genes, expressed in a tissue-specific manner, are thought to regulate differential gene expression [64]. In birds, nuclear estrogen receptors have been reported to be present in the nuclear fractions of GCs of the largest (F1) and second-largest (F2) preovulatory follicles [65]. We confirmed the presence of ESR and, for the first time, demonstrated the presence of two subtypes of ESR (*ESR1*, *ESR2*) in the GCs of mature eggs. Interestingly, in our study, ESR1 was found to be a dominant type of ESR in the GCs of mature egg coats. Interestingly, only the presence of *ESR2* has been, so far, reported in the theca cells of chicken follicles [66]. It was found that ESR2 is a potential transcription factor responsible for the regulation of aromatase expression. The existence of a specific pattern of ESRs in a cell-specific manner in bird follicles suggests the direct action of estrogens on granulosa by ESR1 (our study) and on theca cells by ESR2 [66]. Further studies should focus on the mechanism of action and genes that can be regulated through ESR signaling during the final stages of bird egg maturation.

The mechanism of controlling gene expression by estrogens is known to be genomic and nongenomic, depending on the manner of estrogen–receptor complexes binding to DNA (direct or indirect binding [67]). Detailed analysis of transcripts classified in estrogen receptor signaling indicated a set of estrogen-dependent gene expression transcripts in the turkey egg coat of mature eggs, indicating both genomic and nongenomic mechanisms for the regulation of gene expression. Binding of ESR to DNA promotes the assembly of higher-order transcriptional complexes, containing methyltransferases and histone acetyltransferases [68], which were identified, in our study, in an estrogen-dependent gene expression pathway (Figure 5b). These systems promote transcription by establishing active chromatin marks, and by recruiting general transcription factors and RNA polymerase II. On the other hand, important mediators of indirect genomic signaling, such as stimulating protein-1 (*SP-1*), activator protein-1 (*AP-1*), nuclear factor-κB (*NFKB1*), activating transcription factors (*ATF2*, *JUN*), and nuclear transcription factor-Y (*NFYA*) were also identified in our study. Gene induction by estrogen through the mentioned SP-1 and AP-1 mechanisms have, so far, been well-described in mammals [69,70]. Our study indicated the diversity of mechanisms of ESR transcriptional regulation in GCs of mature egg coats. Further studies should focus on establishing the genes induced by estrogens through genomic and nongenomic mechanisms, and their function in preparing eggs for ovulation.

Interestingly, we also observed the presence of a membrane estrogen receptor, *GPER1*, which, together with ESRs, may be involved in estrogen signaling in mature egg coats. In birds, GPER signaling has been described in the GCs of chicken ovarian follicles before and after cyclic recruitment [28], and its role in GC differentiation and promoting follicle survival has been reported [71]. Estrogen binding to GPER activates a stimulatory G protein, resulting in the stimulation of adenylyl cyclase activity and cAMP levels, which are involved in maintaining the estrogen-induced meiotic arrest of oocytes before fertilization in vertebrates [72]. Meiotic arrest of fish oocytes by estrogen-induced GPER signaling involves the activation of Src family kinases, matrix metalloproteinase (MMP), receptor protein-tyrosine kinase (EGFR), and mitogen-activated protein kinase (MAPK3/1) [73]. The G protein—precisely, subunits α and β (*GNA11-13*, *GNAI1*, *GNAS*, and *GNB1*)—responsible for the activation of adenylyl cyclase, as well as transcripts of Src family kinases, MMPs, and MAPK kinases, were identified in our study, which might support the GPER signaling in bird eggs before ovulation/fertilization. Metaphase arrest in meiotic I or II before fertilization is a common and unique feature of oogenesis in many animal species [74]. However, in birds, it has only been suggested that the meiotic arrest of female germ cells is gradual after hatching [75], and there exists no clear, direct evidence of meiotic arrest during oogenesis. Our results indicated the presence of proteins involved in meiotic arrest, thus strongly supporting this suggestion. Further studies should focus on understanding the role of estrogen in controlling the onset of bird oocyte maturation, including the identification of the receptor through which it acts and the signal transduction pathways that are responsive for receptor activation.

### 3.6. Mitochondrial Oxidative Phosphorylation

**Figure 7:**
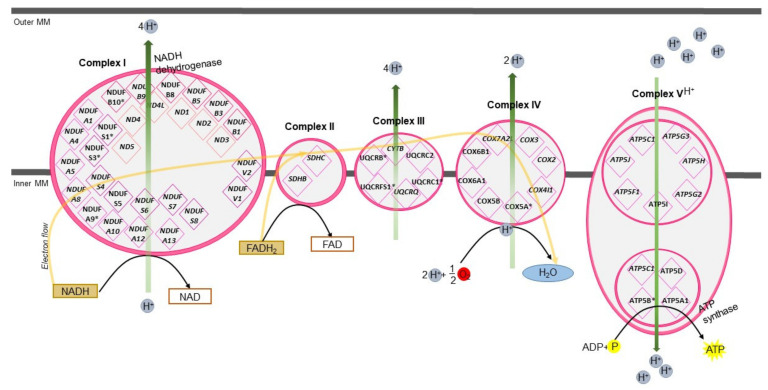
Transcripts/proteins identified in the inner perivitelline layer which participate in the mitochondrial oxidative phosphorylation complexes I–V. Description of gene/protein symbols are presented in the Supplementary Materials, Tables S1 and S3.

In our study, we found that oxidative phosphorylation (OxPhos) and mitochondrial dysfunction were the most significant pathways in the proteome of egg coats at F1 stage in turkey oocytes. Using proteomic and transcriptomic data, we found molecules that participate in the mitochondrial OxPhos complexes I–V in turkey egg coats (Figure 7). In mammals, the increased number of mitochondria and their correct relocation (possibly related to an appropriate distribution of energy within the ooplasm) seems to be critical during oocyte growth, further affecting its developmental competence [76,77]. Bioenergetic deficits that decrease net cytoplasmic ATP levels might compromise the progression of oocyte maturation and the development of preimplantation embryos [78].

In view of this knowledge, it seems surprising that the oxidative phosphorylation pathway occurred as the top canonical pathway in the egg coat mature follicles in turkeys. This phenomenon can be explained by the recent study of Van Blerkom et al. [79], which indicates that cumulus and coronal cells surrounding the egg in mammals may be a significant exogenous source of ATP for the oocyte in general, and for the subplasmalemmal cytoplasm in particular. It has been suggested that cumulus and coronal cells provide ATP to the oocyte through numerous elongated cellular extensions passing through the zona pellucida in transzonal processes, which occur along the entire surface of the oolemma [79,80]. In birds, the mature follicle before ovulation is surrounded by the protein complex IPVL and three cell layers, from which the GC layer closely surrounds the ovum and plays key roles in regulating follicle secretion [1]. It seems possible that, similar to mammals, bird oocyte ATP could be also supplemented by an exogenous source—likely by GCs—which can take place before ovulation as, in birds, ovulated eggs are surrounded only by the IPVL.

### 3.7. The Cellular Response against Oxidative Stress

In mammals, oxidative stress initializes the postovulatory aging process of oocytes through intracellular ROS accumulation and the peroxidation of lipids located in the plasma membrane [81,82]. It should be underlined that bird egg cytoplasm contains an extremely large amount of egg yolk, composed of an abundant amount of lipids, making bird eggs susceptible to lipid peroxidation [1]. Our results indicated that several redox-sensitive signaling pathways involved in the cellular response against oxidative stress through the regulation of transcription factors—including nuclear factor kappa light chain enhancer of activated B cells (NF-kB), MAPK, PI3K/AKT, and NRF2—exist in the egg coat of mature turkey oocytes. Among them, NRF2-mediated oxidative stress response was found to be the most significant pathway, both in the egg coat proteome and transcriptome. In birds, GCs closely surround the ovum before ovulation, and our transcriptomic data indicated the presence of ROS-scavenging transcript-coding proteins, such as *SOD1*, *PRDX1*, *HMOX1*, *TXN*, and *NQO1*, which may be produced in response to ROS under the influence of NRF2. Interestingly, the most strongly expressed transcripts, including *TXN*, *SOD1*, and *PRDX1*, are known to be induced in response to nitric oxide, superoxide, and hydrogen peroxide, respectively. Many studies have demonstrated the role of ROS in the ovulatory process of mammals, suggesting a role of nitric oxide in the inhibition of oocyte meiotic maturation and the initiation of aging changes in the oocyte by superoxide and hydrogen peroxide [83]. The present study provides evidence that GCs of mature eggs in birds can be a source of cellular antioxidant molecules, which may be released in response to oxidative stress in order to protect the mature egg just before ovulation. In other words, it is likely that GCs protect mature eggs against damage caused by oxidative stress. Further research should focus on the action of identified molecules under stress conditions in bird oocytes.

### 3.8. Cell-Cell Junction—Intercellular Interactions of GCs

In our study, we identified the coexistence of tight, adherens, and gap junctions in the egg coat of mature bird follicles for the first time. Among them, the adherens junction and its remodeling were found to be the most significant pathways in both the egg coat transcriptome and proteome. It should be underlined that, in contrast to mammals, where the follicle is released from the ovary together with cumulus cells (cell–cell communication still exists after ovulation), avian ovulated eggs are surrounded only with the IPVL, without surrounding GCs. Therefore, the intercellular interactions in bird oogenesis are important before ovulation, as well as during follicle development and final maturation.

In this paper, we demonstrated the presence of transcripts of the major adherens junction (AJ) proteins in turkey mature egg coats, in particular, cadherins and nectins. Cadherins belong to the class of calcium-dependent transmembrane proteins, which link to the actin cytoskeleton through the intracellular proteins α-catenin and β-catenin (CTNNA, CTNNB [84]). Nectins are calcium-independent transmembrane proteins that bind to the actin cytoskeleton using the cytoplasmic protein afadin (AFDN) [85]. Interestingly, in our study, we identified the abovementioned cell adhesion molecules, as well as actin cytoskeleton proteins, suggesting the important function of AJ for final follicle maturation in birds. Similar to our results, in mammals, CDH2 has been confirmed to be localized in GCs [86,87,88], and CTNNB at the cell membrane junctions between GCs, as well as at the oocyte cell membrane within secondary follicles [89]. So far, in mammals, CDH2 and CTNNB have been indicated to be involved in the follicular function at the late pre-antral and antral stages of follicle development, and cooperation with WNT signaling during follicle formation and maintenance, respectively [14,89]. In birds, the adhesion molecules of ovarian follicles have been correlated with high egg-laying rates in chicken hens [23]. Further studies should focus on the role of identified AJ proteins in GC adhesion, as well as the involvement of AJ proteins in signaling events occurring in mature bird follicles.

In avians, a major tight junction (TJ) protein—OCLN—has been identified during folliculogenesis [90]. Similar to our results, pronounced OCLN staining was observed in the apical region of the GC layer in chicken hen oocytes. It has been shown that OCLN was downregulated during follicular development in vivo, in a developmental stage-specific manner [90]. This explains the low staining intensity observed in the GC layer of mature eggs in our study. Besides occludin, we reported the identification of other important TJ transcripts, such as claudins (*CLDN1*, *CLDN2*) and junctional adhesion molecules (*JAM3*), as well as scaffolding proteins, such as tight junction proteins (*TJP1*, *TJP3*) and *AFDN*, which bind TJs to the cytoskeleton [91,92]. In our study, TJP1 and OCLN were both localized along the apical region of GCs; however, higher intensity was observed for TJP1 proteins, suggesting the important function of TJ proteins in the interaction with actin cytoskeletons of GCs in mature egg coats. Generally, TJ functioning is linked with the rapid and high-capacity transport of macromolecules into the oocyte through a paracellular pathway [88]. In birds, it has been suggested that TJ may be involved in yolk transport. Such high-capacity transport of yolk components may represent a crucial prerequisite for rapid oocyte growth once follicles have entered the follicular hierarchy [90].

Sharing of cytoplasmic contents between cumulus cells and the oocyte is possible due to the gap junction (GJ), which, in mammals, is required for normal follicle development [93]. In birds, it has been indicated that, at the germinal disc region, the cell membranes of GCs and oolemma form GJ [94]. GJs are composed of connexin (Cx) proteins, which form transmembrane channels for small molecules [95,96]. Interestingly, the absence of Cx43 caused GCs to grow in the early preantral stage in mammals [97]. In our study, we identified transcripts of the key gap junction protein, Cx43 (*GJA1*), with high expression, as well as the presence of Cx43 in turkey mature egg coats. Similar to mammals, in turkeys, Cx43 is localized on the surfaces of zona pellucidae [98]. It has been suggested that Cx43 is transported by transzonal projection from cumulus cells during porcine oocyte maturation [98]. This may also be possible for turkey IPVL, as Cx43 was also identified in the cytoplasm of the GC layer surrounding the IPVL. Interestingly, we also observed positive staining between adjacent GCs, suggesting a role of GJ in communication between GCs, as has been suggested for oocyte maturation in mammals [97].

Analysis of the identified transcripts and proteins revealed the presence of molecules important for GJ functioning. We identified transcripts of kinases that play roles in the phosphorylation and acute gating of gap junction channels, such as cGMP-dependent protein kinase (*PRKG1*), protein kinase cAMP-dependent type (*PRKAR1B*), protein kinase C alpha type (*PRKCA*), and a group of mitogen-activated protein kinases (MAPK1, *MAP3K1–5,7–9,13,14,20,21*). In mammals, GJ communication allows for the transit of small (1 kDa) molecules between the oocyte and cumulus cell cytoplasm, such as glucose metabolites (pyruvate), which are important for energy production in the oocyte mitochondria, as well as ATP, cAMP, and cyclic GMP (cGMP), which are necessary to maintain oocyte arrest at prophase I of meiosis [99]. It seems possible that, similar to cumulus cells surrounding mammalian oocytes, the GCs can communicate with maturing eggs through the gap junction in birds. It was suggested above that bird oocyte ATP could be also supplemented by an exogenous source, likely by GCs. Our results open new areas of further studies, which should focus on the role of gap junction proteins in granulosa growth and the regulation of egg development in birds.

## 4. Materials and Methods

### 4.1. Birds, Housing, and Tissue Collection

Laying breed hens aged 38 weeks (Hybrid Converter, Hendrix Genetics Ltd., Kitchener, ON, Canada) were housed individually, under standard husbandry conditions, at the farm belonging to the Department of Poultry Science of the University of Warmia and Mazury in Olsztyn, Poland. Water and feed were provided ad libitum. Hens were exposed to a 15L:9D photoperiod. Tissue samples were collected from liver and preovulatory follicles at the F1 stage (Supplementary Materials, Figure S3a). Ovarian follicles were halved and washed carefully in phosphate buffer saline (PBS, Sigma-Aldrich, St. Louis, MO, USA), in order to remove the yolk. Next, the IPVL was gently separated from the follicle wall, using forceps [16]. For further analysis, we focused on the germinal disc region, surrounded by GCs which are closely adhered to the IPVL and cannot be separated [13]. The IPVL fragments were carefully dissected around the germinal disc (Supplementary Materials, Figure S3b) and washed with PBS. The tissues to be analyzed by RNA-Seq, quantitative reverse transcription polymerase chain reaction (RT-PCR), and two-dimensional electrophoresis (2DE) were immediately frozen in liquid nitrogen. For the immunohistochemical study, IPVL fragments were immediately fixed in Bouin’s fluid (Sigma-Aldrich).

All experiments were performed according to the Polish Animal Welfare Act. No ethics approval was required for these experiments.

### 4.2. Tissue Morphology

For histological analysis of the IPVL, haematoxylin-eosin (H-E) staining was performed, as described previously by Bilińska et al. [100]. Fifty cross-sections were investigated, with regard to the quality of the IPVL with the GC layer. The IPVL sections were examined under a Leica DMR microscope (Leica Microsystems, Wetzlar, Germany). Both the IPVL and the GC layer showed typical morphology, without any loss of cells (Supplementary Materials, Figure S3c).

### 4.3. RNA Isolation and the Evaluation of RNA Integrity

The IPVL (*n* = 8) and liver (*n* = 6) were mechanically disrupted. Then, total RNA was extracted using TRIzol**^®^** Reagent (Invitrogen by Thermo Fisher Scientific Baltics UAB, Vilnius, Lithuania), as described previously by Słowińska et al. [16]. Genomic DNA was removed from RNA samples using a DNase I, Amplification Grade kit (Invitrogen by Thermo Fisher Scientific, Carlsband, CA, USA). The evaluation of RNA quantity and quality was carried out according to the protocol of Słowińska et al. [46]. First, the purity and concentration of isolated RNA were determined using a NanoDrop 1000 spectrophotometer (Thermo Fisher Scientific, Waltham, MA, USA). Then, using automated microfluidic electrophoresis on an Agilent Bioanalyzer 2100 (Agilent Technologies, Santa Clara, CA, USA), the RNA integrity was evaluated. The RNA integrity numbers (RIN) of isolated RNA samples were measured, and samples with an RIN above 8.4 (*n* = 6) were used for further analysis.

### 4.4. Library Preparation and Sequencing Procedures

The TruSeq Stranded mRNA Sample Preparation protocol (Illumina, Inc., San Diego, CA, USA) was used for preparation of six RNA libraries, according to the manufacturer’s procedure. The RNA library construction assumed the capture of poly-A tails of mRNA by magnetic beads. The attached mRNA fragments were cut using divalent cations under increased temperatures. The short pieces of RNA were joined to first-strand cDNA using SuperScript II reverse transcriptase (Invitrogen, Waltham, MA, USA). DNA polymerase I and RNase H participated in second-strand cDNA synthesis. The constructed paired-end reads were strand-specific. The genetic material was then enriched by a PCR procedure and the amplified cDNA constituted the final RNA-Seq library. The libraries were tested by Quantification Protocol Guide (KAPA Library Quantification kits for Illumina Sequencing platforms) and the TapeStation D1000 ScreenTape (Agilent Technologies, Waldbronn, Germany). Next, the indexed RNA-Seq libraries were sequenced using the NovaSeq 6000 platform (Illumina). The paired-end 2 × 100 bp reads obtained for IPVL sequencing were submitted to the European Nucleotide Archive database of EMBL-EBI (https://www.ebi.ac.uk/ena), under accession no. PRJEB46621 (available 25 August 2021).

### 4.5. Quality Control and Mapping Process 

The paired-end raw reads were evaluated using the MultiQC software (v1.10) [101]. Low-quality reads (PHRED score < 20) and/or those containing Illumina adaptor sequences were eliminated using the Trimmomatic software (v0.38). Additionally, all sequences were trimmed to a sequence length of 90 bp, due to the highest sequencing error on both ends of reads. Trimmed reads were mapped to the soft masking reference turkey genome (*Meleagris gallopavo*.Turkey_2.01.dna_sm.toplevel.fa), with ENSEMBL annotation (v99) using the STAR software (v2.7) [102]. The mapping results (BAM files) and ENSEMBL gene description (GTF files) were merged to enrich annotations using the StringTie tool (v1.3.3) [103]. The count and FPKM values were obtained using the python script prepDE.py (StringTie module) and the ballgown software [104], respectively. Big data storage and memory-consuming processes were performed in the Regional IT Center of the University of Warmia and Mazury in Olsztyn, applying a 64-core CPU and 180 GB RAM server.

### 4.6. 2DE and Protein Identification by MALDI TOF/TOF

Three individual samples of the IPVL were used for 2DE. The individual IPVL samples frozen in liquid nitrogen were flooded with a 200 µL extraction buffer containing 7 M urea, 2 M thiourea, 4% CHAPS (*wt*/*vol*), 40 mM dithiothreitol (DTT), 2% pharmalyte 3-10 nonlinear (NL), and 1% of a protease inhibitor cocktail (P8340, Sigma-Aldrich). Then, sample sonication was performed for 30 s using a VC-13 PB (Sonics & Materials, Inc., Newtown, CT, USA), set at 35% relative output. The IPVL extracts were centrifuged at 12,800 × *g* for 20 min at 4 °C. Then, 25 µL of supernatant (in quadruplicate) was immediately precipitated, using a 2-D Clean-up Kit (GE Healthcare, Uppsala, Sweden). The pellet was resuspended in a rehydration buffer consisting of 7 M urea, 2 M thiourea, and 2% CHAPS (*wt*/*vol*). Bradford protein assay [105] with a Coomassie Plus Kit (Thermo Scientific, Waltham, MA, USA) was used for the measurement of the protein concentration. Bovine serum albumin was used as a standard. The samples containing 500 µg of protein were then supplemented with a rehydration solution, to a final concentration of 7 M urea, 2 M thiourea, 4% CHAPS, 2% pharmalyte 3-10 nonlinear, 40 mM DTT, and a trace amount of bromophenol to a final volume of 450 µL, then loaded onto immobiline DryStrip gels (length 24 cm, pH 3–10 NL, GE Healthcare) with overnight passive rehydration. Isoelectric focusing (IEF) was performed according to the protocol described by Słowińska et al. [106], using an Ettan IPGphor apparatus (GE Healthcare) at 20 °C with current limited to 50 µA/strip and the following voltage program: Step 1 at 500 V for 3.5 h, step 2 at 1000 V for 1 h in gradient, step 3 at 8000 V for 3.0 h in gradient, and step 4 at 8000 V for 5.5 h (for a total focusing time of 60,800 Vh). Before SDS-PAGE, the IPG strips were equilibrated in 6 M urea, 75 mM Tris-HCl (pH 8.8), 2% SDS, 30% glycerol, and 1% DTT (*w*/*v*) for 15 min. Then, for the next 15 min, the strips were equilibrated in the same solution, but with 2.5% iodoacetamide (*w*/*v*) instead of DTT and trace bromophenol blue. After equilibration, two-dimensional electrophoresis was performed using precast 12.5% SDS polyacrylamide gels (DALT Gel; GE Healthcare). The gels were run at standard conditions, as described previously by Słowińska et al. [106] (i.e., 1 W per gel for the first 1 h, then 17 W per gel for approximately 4.5 h, until the dye front reached the bottom of the gel). The gels were fixed in 10% acetic acid and 40% ethanol, and the protein spots were visualized after staining in Colloidal Coomassie Blue G-250 (Sigma) working solution (8% ammonium sulfate, 0.8% phosphoric acid, 0.008% Coomassie Blue G-250, and 20% methanol). All gels were scanned with an Image Scanner III (GE Healthcare), and the relative volume (%Vol) was analyzed using an Image Master 2D platinum (v7.05, GE Healthcare) for spot quantification. All visualized protein spots were picked manually and were subjected to in-gel trypsin digestion, according to the procedure described by Słowińska et al. [16]. The digested samples were spotted using the dried droplet method on an MTP 384 target plate ground steel (Bruker Daltonics, Bremen, Germany). Mass spectra were acquired using the operating conditions previously described in detail by Słowińska et al. [107], in the range of 700–3500 *m*/*z* using a MALDI-TOF autoflex speed TOF/TOF mass spectrometer equipped with a Smartbeam II laser (355 nm; Bruker Daltonics). The database search criteria were as follows: Enzyme, trypsin; fixed modification, carbamidomethylation (C); variable modifications; oxidation (M) peptide mass tolerance of 100 ppm; fragment mass tolerance of 0.7 Da; and one missed cleavage allowed. The search results were filtered using a significant threshold of *p* < 0.05 and a MASCOT ion score cutoff of ≥30.

### 4.7. Functional Analysis

The ingenuity pathway analysis (IPA; IngenuityR Pathway Analysis, IPAR, Qiagen, Redwood City, CA, USA) software was used to investigate the canonical and functional pathways of the IPVL transcriptome and proteome of hens. Fisher’s exact test and Benjamini–Hochberg corrections were performed to calculate the significance (*p* < 0.05) of canonical and functional pathways [108]. The possible interactions between transcripts involved in the protein ubiquitination pathway and estrogen receptor signaling were established by STRING (Search Tool for Retrieval of Interacting Genes, [109]), with a highest confidence score cut-off of 0.9. The ubiquitination of proteins and protein synthesis by eIF2 signaling and eIF4 and p70S6K signaling were constructed using the BioRender software (BioRender, San Francisco, CA, USA).

### 4.8. Phylogenetic Analysis

Phylogenetic analyses of the ZP gene family were performed on 276 sequences of protein-coding genes (CDS) shared by 52 species belonging to bird genera, as well as four outgroups represented by one species of mammals, reptiles, amphibians, and fish. The appropriate CDS sequences were downloaded from the ENSEMBL database (v103; https://www.ensembl.org/, accessed on 10 March 2021). The chosen sequences were aligned using MAFFT (v7) [110]. Multiple sequence alignments were trimmed using the trimal software (v1.4.1) [111]. The Bayesian inference (BI) method was applied to reconstruct phylogenetic relations within the ZP gene family, using the MrBayes software (v3.2.6) [112]. The best substitution model was selected, according to Mega (v7) [113], and the model GTR + G was fitted. A BI partitioning analysis was performed, assigning a majority rule consensus tree with 1 × 10^6^ generations, using the Markov Chain Monte Carlo (MCMC) method. Tree sampling frequency was equal to 10,000 generations. The first 2000 trees were discarded as burn-in, with a random starting tree.

### 4.9. Immunohistochemistry

The IPVL was fixed in Bouin’s solution (saturated picric acid, formaldehyde, and glacial acetic acid at a proportion of 15:5:1) for 24 h, dehydrated in a growing gradient of ethanol, and inserted in paraffin. Then, all sections (5 µm) were deparaffinized, rehydrated through decreasing alcoholic solutions, and rinsed in water [16].

In turn, slides were dipped in 10 mM citrate buffer (pH 6.0), then in Tris-EDTA buffer (10 mM Tris with 1mM EDTA and 0.05% Tween 20; pH 9.0), and heated for approximately 5 min in a microwave oven (750 W) for antigen retrieval. The complete procedure was related to one described previously [114]. To neutralize endogenous peroxidase activity, incubation was carried out in hydrogen peroxide solution (0.3%, *v*/*v*) in Tris-buffered saline (TBS; 0.05 M Tris-HCl, 0.15 M NaCl, pH 7.6) for 10 min, while nonspecific binding sites were blocked with 10% (*v*/*v*) goat or horse serum for 20 min at ambient temperature. Following overnight incubation with primary antibodies at 4 °C (Supplementary Materials, Table S5), biotinylated goat anti-rabbit or horse anti-mouse secondary antibody IgG (Vector Laboratories, Burlingame, CA, USA) at 1:400 dilution was applied for 1 h at ambient temperature. An avidin-biotinylated horseradish peroxidase complex (ABC/HRP; 1:100; VECTASTAIN Elite ABC Reagent, Vector Laboratories) was used to amplify the target antigen signal, followed by 0.05% 3,30-diaminobenzidine tetrachloride (DAB; Sigma-Aldrich) in TBS containing 0.01% H_2_O_2_ and 0.07% imidazole. After each stage of the above procedure, the sections were carefully rinsed with TBS. Then, the sections were stained with Mayer’s haematoxylin, dehydrated, and fixed using a DPX mounting medium (Sigma-Aldrich).

All slides were treated with the same conditions, such that the staining intensity was comparable between various sections [115]. Negative controls contained sections that were incubated with irrelevant IgG, instead of primary antibodies. All immunohistochemical analyses were repeated at least three times. Sections were then analyzed under a Leica DMR microscope (Leica Microsystems, Wetzlar, Germany).

### 4.10. Identification of Ubiquitinated Proteins in the IPVL 

Aliquots of the IPVL extracts (100 µg) in a final volume of 125 µL of rehydration buffer (7 M urea, 2 M thiourea, 4% CHAPS *wt*/*vol*, 40 mM DTT, and 2% pharmalyte 3-10 NL), were applied to 7 cm IPG strip (pH range 3 to 10 NL; GE Healthcare). Then, IEF and two-dimensional electrophoresis were conducted as described in our previous study [115]. After 2DE, hen IPVL proteins were transferred to a nitrocellulose (NC) membrane (0.2 µm, Sigma-Aldrich) in a Mini Trans-Biol Cell (Bio-Rad, Hercules, CA, USA), according to Słowińska et al. [116]. The NC membranes were incubated overnight at 4 °C with anti-ubiquitin antibodies (U5379, Sigma-Aldrich) diluted with TBS-T (0.05 M Tris-HCl, 0.15 M NaCl, and 0.1% Tween 20; pH 7.6) at a ratio of 1:100. Blots were scanned with a VersaDoc MP 4000 system (Bio-Rad). The proteins of interest were excised manually, according to the Western signal, and prepared for mass spectrometry identification according to the protocol developed by Luque-Garcia et al. [117]. Antibodies were removed by three washes of the protein spots with 20 mM sodium bicarbonate buffer (pH 7.4), followed by washing with 100 mM glycine (pH 2.4). On-membrane digestion and sample preparation for mass spectrometry were performed according to our previous study [16].

### 4.11. Quantitative Real-Time Reverse Transcriptase PCR

Total RNA was extracted from the IPVL (the same as used in RNA-Seq; *n* = 6) and liver (*n* = 6) fragments using TRIzol^®^ Reagent (Invitrogen by Thermo Fisher Scientific Baltics UAB, Vilnius, Lithuania). Then, a DNase I, Amplification Grade kit (Invitrogen by Thermo Fisher Scientific, Carlsbad, CA, USA) was used for genomic DNA removal from RNA samples. The purity and concentration of isolated RNA were determined using a NanoDrop 1000 spectrophotometer (Thermo Fisher Scientific, Waltham, MA, USA). cDNA was synthesized using a High-Capacity cDNA Reverse Transcription Kit (Applied Biosystems by Thermo Fisher Scientific Baltics UAB), according to the manufacturer’s specifications. Expression of specific mRNA was quantified with Custom TaqMan Gene Expression Assays (Applied Biosystems by Thermo Fisher Scientific, Pleasanton, CA, USA). Primer–probe sets for glyceraldehyde-3-phosphate dehydrogenase (*GAPDH*) were designed based on predicted sequences obtained from the National Center for Biotechnology Information (NCBI). The sequences for ZP genes (*ZP1-4*, *ZPD*, *ZPAX*), estrogen receptor genes (estrogen receptors (*ESR1*, *ESR2*) and G protein-coupled estrogen receptor 1 (*GPER1*)), and antioxidant enzymes (peroxiredoxin 1 (*PRDX1*), superoxide dismutase 1 (*SOD1*), and thioredoxin (*TXN*)) were downloaded from the ENSEMBL database (https://www.ensembl.org). The Primer3Plus online tool (https://www.bioinformatics.nl/cgi-bin/primer3plus/primer3plus.cgi) was used for primer–probe set design. Gene names and primer–probe set information are presented in the Supplementary Materials, Table S6. Reactions were performed using an ABI ViiA™7 sequence detection system (Applied Biosystems by Life Technologies, Singapore) with the following conditions: 10 min at 95 °C, 45 cycles of 15 s at 95 °C, and 1 min at 60 °C. All results were normalized to *GAPDH* expression, as endogenous control, using the PCR Miner algorithm [118].

#### Statistical Analysis

One-way analysis of variance (ANOVA), followed by Tukey’s multiple comparison test, was performed for the analysis of NGS and RT-PCR results, involving: six zona pellucida glycoprotein transcripts/genes (*ZP1*, *ZP2*, *ZP3*, *ZP4*, *ZPD,* and *ZPAX*), six sirtuin transcripts (*SIRT1-6*), three transcripts/genes involved in estrogen receptor signaling (*ESR1*, *ESR2*, and *GPER1*), and five transcripts/three genes involved in NRF2-mediated oxidative stress response (*PRDX1*, *SOD1*, *TXN*, *HMOX1*, and *NQO1*). A Student’s *t*-test was used for comparison of the expression level of the ZP1 gene between the liver and the IPVL. The data are shown as means ± standard deviation (SD). All analyses were performed using GraphPad statistical software (GraphPad PRISM v 8.4.1, GraphPad Software Inc. San Diego, CA, USA).

## 5. Conclusions

In summary, our results extended the knowledge regarding gene expression in F1 follicles. Six subfamilies of ZP genes (*ZP1–4*, *ZPD*, and *ZPAX*) were identified for the first time in turkey hen oocytes/GCs at the F1 developmental stage. The GCs were found to be a rich source of *ZPD* in mature eggs. The main expression site for *ZP1* was found in the liver; however, GCs may also partially participate in local ZP1 secretion. Phylogenetic analysis indicated that turkey ZP glycoproteins are conserved among galliforms. A ubiquitination system was identified in mature oocytes, and ZP1 was found to be the main ubiquitinated protein in the IPVL. Detailed analysis of transcripts classified in estrogen receptor signaling indicated the presence of *ESR1* and *ESR2,* as well as a set of estrogen-dependent genes involved in both genomic and nongenomic mechanisms for the regulation of gene expression by estrogen. Oxidative phosphorylation was found to be a possible source of ATP in the mature bird oocyte, and the NRF2-mediated oxidative stress response signaling pathway could be involved in the response against oxidative stress. Oocyte–granulosa cell communication by tight, adherens, and gap junctions seems to be essential for the final step of oocyte maturation.

## Figures and Tables

**Table 1:** Overall Statistics of Sequencing and Mapping Results of Six RNA-Seq Libraries Extracted from the Inner Perivitelline Layer.

IPVL Samples	O1	O2	O3	O4	O5	O6
row reads	1.03 × 10^8^	1.18 × 10^8^	1.05 × 10^8^	1.16 × 10^8^	1.2 × 10^8^	1.01 × 10^8^
trimmed reads	1.02 × 10^8^	1.16 × 10^8^	1.03 × 10^8^	1.14 × 10^8^	1.19 × 10^8^	0.99 × 10^8^
mapped	82307430	93758986	87048822	96081072	99511690	83720988
uniquely mapped	80362334	91783236	84612194	93744112	97406022	81650560
mapped to coding regions (%)	41.90	40.75	43.94	44.59	43.72	43.38
mapped to UTR (%)	7.18	7.20	5.07	5.22	5.38	5.47
mapped to introns (%)	5.30	5.14	7.13	7.61	7.70	7.51
mapped to intergenic regions (%)	45.62	46.90	43.85	42.58	43.20	43.64
multi-mapped	1,940,566	1,969,616	2,431,930	2,333,492	2,102,302	2,065,832
too many loci	4530	6134	4698	3468	3366	4596

The names of samples are similar to names in the EMBL-EBI databases, where O1-O6 refer to the IPVL1–IPVL6 samples, respectively.

**Table 2:** Results from Ingenuity Pathway Analysis of Turkey Inner Perivitelline Layer Transcripts and Proteins.

*p* Value	No Molecules	Functional Analysis	*p* Value	No Molecules
Trancriptome		**Ingeniuty Canonical Pathways**	Proteome	
1.05 × 10^28^	142	*Protein Ubiquitination Pathway*	9.18 × 10^8^	15
1.35 × 10^26^	121	*EIF2 Signaling*	1.43 × 10^5^	11
7.81 × 10^20^	86	*Regulation of eIF4 and p70S6K Signaling*	1.66 × 10^4^	8
6.05 × 10^18^	128	*Sirtuin Signaling Pathway*	6.00 × 10^11^	11
8.65 × 10^17^	137	*Estrogen Receptor Signaling*	1.57 × 10^3^	10
9.21 × 10^17^	86	*Mitochondrial Dysfunction*	1.89 × 10^18^	22
4.68 × 10^15^	89	*NRF2-mediated Oxidative Stress Response*	6.02 × 10^12^	17
1.32 × 10^14^	95	*mTOR Signaling*	4.83 × 10^3^	7
9.55 × 10^11^	54	Oxidative Phosphorylation	1.20 × 10^18^	19
8.33 × 10^6^	31	Remodeling of Epithelial Adherens Junctions	9.18 × 10^11^	10
1.80 × 10^5^	22	BAG2 Signaling Pathway	3.05 × 10^10^	9
		Gluconeogenesis I	4.50 × 10^9^	7
4.38 × 10^7^	79	Actin Cytoskeleton Signaling	3.62 × 10^8^	14
		**Molecular and Cellular Function**		
7.31 × 10^10^–3.36 × 10^70^	760	*Protein Synthesis*	2.91 × 10^5^–1.47 × 10^16^	68
1.86 × 10^14^–2.18 × 10^68^	376	*RNA Post-Transcriptional Modification*		
9.96 × 10^10^–1.18 × 10^53^	1728	*Cell Death and Survival*	5.64 × 10^5^–4.34 × 10^24^	127
1.32 × 10^18^–1.68 × 10^40^	380	*Protein Degradation*		
8.39 × 10^10^–4.77 × 10^34^	1048	*Molecular Transport*		
		Post-Transcriptional Modification	5.05 × 10^5^–2.93 × 10^22^	43
		Protein Folding	5.05 × 10^5^–4.02 × 10^21^	22
		Cellular Compromise	1.71 × 10^6^–2.20 × 10^15^	46
		**Physiological System Development** **and Function**		
1.15 × 10^12^–6.47 × 10^59^	1242	*Organismal Survival*	9.44 × 10^10^–9.44 × 10^10^	76
1.81 × 10^10^–3.72 × 10^24^	1012	*Embryonic Development*	4.43 × 10^7^–8.27 × 10^9^	23
3.60 × 10^10^–3.72 × 10^24^	1413	*Organismal Development*	3.55 × 10^5^–8.27 × 10^9^	55
1.86 × 10^11^–3.72 × 10^24^	371	*Tissue Morphology*		
5.78 × 10^10^–1.06 × 10^19^	542	*Connective Tissue Development and Function*		
		Tissue Development	1.13 × 10^5^–6.47 × 10^59^	52
		Organ Development	2.73 × 10^6^–8.27 × 10^9^	30
Score		**Top Networks**	Score	
28	35	*Molecular Transport, RNA Post-Transcriptional Modification, RNA Trafficking*		
28	35	*Protein Synthesis. RNA Damage and Repair*		
28	35	*Post-Translational Modification, Protein Degradation, Protein Synthesis*		
		Cellular Assembly and Organization, RNA Post-Transcriptional Modification	57	29
		Cell Death and Survival, Drug Metabolism. Small Molecule Biochemistry	46	25
		Gene Expression, Post-Translational Modification, Protein Folding	39	22
		Cell Death and Survival, Gene Expression Protein Synthesis	32	19

*IPA*-obtained results from transcriptome data are presented in italics, IPA-obtained results from proteome data are shaded in gray.

## Data Availability

The data obtained for IPVL sequencing were submitted to the European Nucleotide Archive database of EMBL-EBI (https://www.ebi.ac.uk/ena), under accession no. PRJEB46621. Proteomic raw data are available upon request from the corresponding author.

## Supplementary Materials

The following are available online at www.mdpi.com/1422-0067/22/19/10589/s1.
