# Supplementary material for: Transcriptome and Proteome Analysis Revealed Key Pathways Regulating Final Stage of Oocyte Maturation of the Turkey (Meleagris gallopavo)
Source: Int J Mol Sci. 2021 Sep 30;22(19):10589. doi: 10.3390/ijms221910589 (PMC8508634; doi:10.3390/ijms221910589)
Supplement: Supplementary file 1 [file ijms-22-10589-s001.zip › Table S3.pdf]

Table S3. Detailed information on the identification of proteins in turkey inner perivitelline layer by MALDI TOF/TOF analysis.

						Precursor mass			
**Spot**	**Protein name**	**Gene symbol**	**Sequence coverage (%)**	**Mascot score**	**Calculated pI/ Nominal mass (Mr)**	**observed**	**theoretical**	**Peptide score**	**Peptide sequence**
1	PREDICTED: calreticulin [Meleagris gallopavo] XP_010726340.1	CALR	54	583	43173/4.29	1147.6730	1146.6550	60	K.KVHVIFNYK.G
						1019.5789	1018.5600	53	K.VHVIFNYK.G
						1833.8756	1832.8469	63	K.VESGSLEDDWDFLPPK.K
						1961.9638	1960.9418	69	K.VESGSLEDDWDFLPPKK.I
						1586.7377	1585.7049	124	K.FAEEFGNETWGATK.E
	calreticulin, partial [Gallus gallus] AAS49610.1	CALR	56	583	47079/4.41	1147.6730	1146.6550	60	K.KVHVIFNYK.G
						1019.5789	1018.5600	53	K.VHVIFNYK.G
						1833.8756	1832.8469	63	K.VESGSLEDDWDFLPPK.K
						1961.9638	1960.9418	69	K.VESGSLEDDWDFLPPKK.I
2	PREDICTED: calreticulin [Meleagris gallopavo] AAS49610.1	CALR	40	345	43173/4.29	1019.5847	1018.5600	53	K.VHVIFNYK.G
						1586.7376	1585.7049	123	K.FAEEFGNETWGATK.E
						1320.6176	1319.5775	61	K.MKEQQDEEQR.Q
	calreticulin, partial [Gallus gallus] XP_010726340.1	CALR	45	458	47079/4.41	1072.5228	1071.4873	43	K.FYGDAEKDK.G
						1019.5847	1018.5600	53	K.VHVIFNYK.G
						1586.7376	1585.7049	123	K.FAEEFGNETWGATK.E
						1320.6176	1319.5775	61	K.MKEQQDEEQR.Q
3	calumenin [Numida meleagris] XP_021239488.1	CALU	12	169	36952/4.47	954.4915	953.4607	34	R.EQFVEFR.D
						1561.7619	1560.7168	90	R.HLVYESDQDKDGR.L
4	reticulocalbin-3-like, partial [Phasianus colchicus] XP_031467421.1	RCN3	16	278	26759/4.61	2994.3479	2993.3217	199	R.DLPGVPHDDTHGFSYDHDAFLGPEEAR.A
						1332.6459	1331.6470	68	R.AFQQLQPEESR.R
5	PREDICTED: protein disulfide-isomerase, partial [Meleagris gallopavo] XP_010719740.1	P4HB	42	466	45917/4.54	1175.6090	1174.5870	44	R.EADDIVSWLK.K
						1819.9068	1818.8901	35	K.ILFIFIDSDHSDNQR.I
						966.5626	965.5586	51	R.ILEFFGLK.K
						1341.6053	1340.5884	57	K.NFEEVAFDENK.N
						942.5228	941.5334	43	K.IHSFPTLK.F
						838.4044	837.4133	56	K.FFPAGSGR.N

6	PREDICTED: protein disulfide-isomerase, partial [Meleagris gallopavo] XP_010719740.1	P4HB	33	326	45917/4.54	1341.5709	1340.5884	66	K.NFEEVAFDENK.N
						942.5283	941.5334	42	K.IHSFPTLK.F
						838.4150	837.4133	56	K.FFPAGSGR.N
						1079.4965	1078.5043	45	R.NVIDYNGER.T
7	PREDICTED: complement component 1 Q subcomponent-binding protein, mitochondrial, partial [Meleagris gallopavo] XP_010720207.1	C1QBP	12	224	25785/4.52	1626.8451	1625.7798	103	K.VSGGWELEVHGTEAR.L
						1509.7407	1508.6783	110	R.EVSFQPTGESDWK.D
8	protein SET, partial [Cathartes aura] KFP47368.1	SET	46	471	16705/4.80	2195.0988	2194.0138	130	K.EQQEAIEHIDEVQNEIDR.L
						1273.7225	1272.7152	38	R.LNEQASEEILK.V
						1208.6626	1207.6554	80	R.VEVTEFEDIK.S
						1840.8678	1839.8606	81	R.IDFYFDENPYFENK.V
9	PREDICTED: complement component 1 Q subcomponent-binding protein, mitochondrial-like [Meleagris gallopavo] gi|733916121|ref|XP_01072020	C1QBP	12	224	25785/4.52	1626.8451	1625.7798	102	K.VSGGWELEVHGTEAR.L
						1509.7407	1508.6783	109	R.EVSFQPTGESDWK.D
10	PREDICTED: vimentin [Meleagris gallopavo] XP_010711008.2	VIM	29	385	46056/4.78	1115.5651	1114.5618	39	K.VELQELNDR.F
						1254.5603	1253.5598	66	R.LGDLYEEEMR.E
						1093.5216	1092.5200	55	K.FADLSEAANR.N
						1836.7613	1835.7922	114	R.DGQVINETSQHHDDLE
11	PREDICTED: vimentin [Meleagris gallopavo] XP_010711008.2	VIM	42	434	46056/4.78	1254.5552	1253.5598	83	R.LGDLYEEEMR.E
						1093.5192	1092.5200	59	K.FADLSEAANR.N
						1121.5748	1120.5764	30	R.EYQDLLNVK.M
						1836.7516	1835.7922	118	R.DGQVINETSQHHDDLE
12	PREDICTED: vimentin [Meleagris gallopavo] XP_010711008.2	VIM	40	384	46056/4.78	1254.5916	1253.5598	144	R.LGDLYEEEMR.E
						1309.6295	1308.5986	44	K.NLQEAEEWYK.S
						1093.5502	1092.5200	30	K.FADLSEAANR.N
						1836.8162	1835.7922	102	R.DGQVINETSQHHDDLE
13	PREDICTED: vimentin [Meleagris gallopavo] XP_010711008.2	VIM	25	353	46056/4.78	1254.5567	1253.5598	70	R.LGDLYEEEMR.E
						1093.5269	1092.5200	64	K.FADLSEAANR.N
						1836.7529	1835.7922	111	R.DGQVINETSQHHDDLE

14	PREDICTED: endoplasmin [Meleagris gallopavo] XP_003202424.1	HSP90B1	21	316	91683/4.81	1200.6555		46	K.FAFQAEVNR.M
15	Endoplasmin, partial [Phaethon lepturus] KFQ80494.1	HSP90B1	40	613	90377/4.82	1081.5562	1080.5352	66	K.FAFQAEVNR.M
						1200.6790	1199.6662	74	K.SILFVPNSAPR.G
						1595.7066	1594.7160	50	R.VFITDDFHDMMPK.Y
						973.4434	972.4341	54	K.YNDTFWK.E
						2355.0329	2354.0783	139	R.LTQSPCALVASQYGWSGNMER.I
16	Endoplasmin, partial [Struthio camelus australis] KFV83935.1	HSP90B1	34	456	90381/4.82	1081.5503	1080.5352	73	K.FAFQAEVNR.M
						1200.6796	1199.6662	73	K.SILFVPNSAPR.G
						2355.0346	2354.0783	121	R.LTQSPCALVASQYGWSGNMER.I
						876.4616	875.4501	46	K.TFEINPR.H
17	PREDICTED: heat shock protein HSP 90-alpha [Meleagris gallopavo] XP_010710229.1	HSP90AA1	27	922	84466/5.01	2255.9136	2254.9516	171	K.HNDDEQYAWESSAGGSFTVR.L
						1311.5643	1310.5626	85	K.EDQTEYLEER.R
						1217.6381	1216.6353	46	K.HSQFIGYPIR.L
						1833.7533	1832.7741	137	R.NPDDITNEEYGEFYK.S
						1513.7741	1512.7784	44	R.GVVDSEDLPLNISR.E
						948.4419	947.4389	51	K.FYEQFSK.N
						1168.5603	1167.5632	73	K.LGIHEDSQNR.K
						[illegible]	[illegible]	[illegible]	[illegible]
18	vimentin [Numida meleagris] XP_021243478.1	VIM	51	578	53271/5.13	1587.7411	1586.7900	38	R.TNEKVELQELNDR.F
						1115.5342	1114.5618	31	K.VELQELNDR.F
						1254.5314	1253.5598	93	R.LGDLYEEEMR.E
						1093.4910	1092.5200	59	K.FADLSEAANR.N
19	vimentin [Numida meleagris] XP_021243478.1	VIM	58	628	53271/5.13	1364.6965	1363.7208	34	R.YSLGSALRPSSAR.Y
						1587.7556	1586.7900	92	R.TNEKVELQELNDR.F
						1115.5385	1114.5618	48	K.VELQELNDR.F
						1254.5383	1253.5598	76	R.LGDLYEEEMR.E
						1093.4986	1092.5200	58	K.FADLSEAANR.N
						1836.7283	1835.7922	101	R.DGQVINETSQHHDDLE
20	transitional endoplasmic reticulum ATPase [Gallus gallus] NP_001038129.1	VCP	35	501	89953/5.14	1172.6220	1171.6601	45	R.GILLYGPPGTGK.T
						1329.6434	1328.6837	64	R.WALSQSNPSALR.E
						1578.7187	1577.7871	67	K.AIANECQANFISIK.G
						1190.4935	1189.5298	41	R.RDHFEEAMR.F
						1034.3897	1033.4287	60	R.DHFEEAMR.F

						1629.7299	1628.7981	52	R.KYEMFAQTLQQSR.G
	PREDICTED: transitional endoplasmic reticulum ATPase [Python bivittatus] XP_007442861.1	VCP	37	515	85157/5.17	1172.6220	1171.6601	45	R.GILLYGPPGTGK.T
						1329.6434	1328.6837	64	R.WALSQSNPSALR.E
						1578.7187	1577.7871	67	K.AIANECQANFISIK.G
						1190.4935	1189.5298	41	R.RDHFEEAMR.F
						1034.3897	1033.4287	60	R.DHFEEAMR.F
						1629.7299	1628.7981	52	R.KYEMFAQTLQQSR.G
21	PREDICTED: LOW QUALITY PROTEIN: hypoxia up-regulated protein 1-like [Meleagris gallopavo] gi|326933411|ref|XP_00321279	HYOU1	32	527	107246/5.11	1531.7943	1530.7790	53	K.QIDNPQVALYQSR.F
						1859.9298	1858.9174	54	K.DSGTQPQLQIQGIGFDR.T
						1492.7890	1491.7722	66	R.DAAVFPIQVEFTR.E
						1628.8045	1627.7954	90	K.QQSPPPDQAEAVHPK.E
22	PREDICTED: hypoxia up-regulated protein 1 [Meleagris gallopavo] XP_010722064.1	HYOU1	16	356	109121/5.12	1531.7307	1530.7790	83	K.QIDNPQVALYQSR.F
						1370.6421	1369.6878	59	R.FPEHELVKDEK.R
						1085.4970	1084.5302	32	K.LFNDQHPSK.D
						1628.7242	1627.7954	108	K.QQSPPPDQAEAVHPK.E
23	PREDICTED: hypoxia up-regulated protein 1 [Meleagris gallopavo] XP_010722064.1	HYOU1	21	495	109121/5.12	1531.7410	1530.7790	107	K.QIDNPQVALYQSR.F
						1370.6635	1369.6878	69	R.FPEHELVKDEK.R
						1693.7950	1692.8471	34	K.DAVITVPAYFNQAER.R
						1085.5105	1084.5302	55	K.LFNDQHPSK.D
						1628.7458	1627.7954	106	K.QQSPPPDQAEAVHPK.E
24	PREDICTED: hypoxia up-regulated protein 1 [Meleagris gallopavo] XP_010722064.1	HYOU1	23	451	109121/5.12	1531.7516	1530.7790	101	K.QIDNPQVALYQSR.F
						1331.6533	1330.6768	60	R.GLAEEFAEQPIK.D
						1693.8053	1692.8471	44	K.DAVITVPAYFNQAER.R
						1085.5132	1084.5302	36	K.LFNDQHPSK.D
						1628.7497	1627.7954	108	K.QQSPPPDQAEAVHPK.E
25	PREDICTED: hypoxia up-regulated protein 1 [Meleagris gallopavo] XP_010722064.1	HYOU1	11	373	109121/5.12	1531.8112	1530.7790	99	K.QIDNPQVALYQSR.F
						1859.9470	1858.9174	122	K.DSGTQPQLQIQGIGFDR.T
						1492.8067	1491.7722	91	R.DAAVFPIQVEFTR.E
26	PREDICTED: LOW QUALITY PROTEIN: hypoxia up-regulated protein 1-like [Meleagris gallopavo] gi|326933411|ref|XP_00321279	HYOU1	30	582	107246/ 5.11	1238.6357	1237.6376	52	R.LFGDSALGMSIK.T
						1531.7653	1530.7790	93	K.QIDNPQVALYQSR.F
						1859.8932	1858.9174	120	K.DSGTQPQLQIQGIGFDR.T
						1492.7605	1491.7722	89	R.DAAVFPIQVEFTR.E
						1628.7630	1627.7954	45	K.QQSPPPDQAEAVHPK.E

27	desmin [Meleagris gallopavo] XP_010711942.1	DES	53	431	53537/5.29	1268.6471	1267.5754	60	R.VAEMYEEELR.E
						1455.7825	1454.6790	132	R.FAGEAGGYQDTIAR.L
28	MICOS complex subunit MIC60 [Gallus gallus] NP_001006462.1	IMMT	38	558	79543/5.72	1222.6286	1221.5877	30	K.VEFEQNLSEK.L
						1824.8825	1823.8649	150	K.GIEQAVESHAVAEEEAR.K
						1353.7201	1352.6949	66	R.RVAHDWLTEAR.M
						1197.6241	1196.5938	80	R.VAHDWLTEAR.M
29	MICOS complex subunit MIC60 [Gallus gallus] NP_001006462.1	IMMT	43	531	79543/5.72	1824.8677	1823.8649	125	K.GIEQAVESHAVAEEEAR.K
						1023.5151	1022.5032	38	R.GVYSEEALR.A
						1353.7055	1352.6949	32	R.RVAHDWLTEAR.M
						1197.6094	1196.5938	58	R.VAHDWLTEAR.M
30	PREDICTED: alpha-actinin-1 isoform X5 [Pseudopodoces humilis] XP_005522612.1	ACTN1	34	513	107832/5.36	1392.6186	1391.6405	49	K.TFTAWCNSHLR.K
						1174.5835	1173.5965	33	K.EGLLLWCQR.K
						2051.8834	2050.9517	52	K.VLAVNQENEQLMEDYEK.L
						1028.5289	1027.5451	42	R.TIPWLENR.A
						1753.7790	1752.8179	78	K.KHEAFESDLAAHQDR.V
						1338.5901	1337.6034	45	K.GISQEQMNEFR.A
						993.4305	992.4464	42	R.ASFNHFDR.K
31	calmodulin, partial [Mauremys japonica] BAB32439.1	CALM1	34	351	15349/4.05	1352.6412	1351.5925	95	K.MKDTDSEEEIR.E
						1596.7490	1595.7063	69	K.DTDSEEEIREAFR.V
						1755.8943	1754.8475	159	R.VFDKDGDGYISAAELR.H
32	PREDICTED: LOW QUALITY PROTEIN: thioredoxin [Meleagris gallopavo] XP_003213740.2	TXN	46	269	12800/5.12	1522.7425	1521.7472	74	K.MIKPFFHSLCDK.F
						1538.7347	1537.7421	80	K.MIKPFFHSLCDK.F + Oxidation (M)
						1221.5351	1220.5358	62	K.CMPTFQFYK.N
						1237.5367	1236.5308	38	K.CMPTFQFYK.N + Oxidation (M)
						1107.5580	1106.5720	41	K.KVQEESGANK.F
33	PREDICTED: hypothetical protein LOC100549033, partial [Meleagris gallopavo] gi|326934995|ref|XP_003213567.1|		37	316	15326/6.14	1660.7876	1659.7709	90	K.QQSVLGPEMEQMQR.S
						1692.7819	1691.7607	37	K.QQSVLGPEMEQMQR.S
						970.6026	969.5495	50	R.SNPLLGLEK.R
						1785.9153	1784.8846	136	K.GGEGIAAPIGPSSYPWAR.N
34	Coactosin-like, partial [Chlamydotis macqueenii] KFP43723.1	COTL1	39	353	16079/5.33	864.4536	863.4501	50	R.EAYNLVR.D
						1424.6692	1423.6620	106	R.DDATEVNWVTFK.Y
						934.4994	933.4920	49	K.EVVQNFAK.E
						1979.9245	1978.9524	107	K.EFVISDHKELDEDYIK.N

35	PREDICTED: eukaryotic translation initiation factor 5A-1 [Coturnix japonica] XP_015706238.1	EIF5A	40	211	17122/5.09	2160.9605<br>2176.9536<br>2032.8610<br>2048.8572	2159.9980<br>2175.9929<br>2031.9030<br>2047.8979	95<br>77<br>74<br>55	K.KYEDICPSTHNMDVPNIK.R<br>K.KYEDICPSTHNMDVPNIK.R + Oxidation (M)<br>K.YEDICPSTHNMDVPNIK.R
36	PREDICTED: protein canopy homolog 2 [Coturnix japonica] XP_015742545.1	CNPY2	34	354	22071/5.51	1203.5096<br>939.4707<br>1730.8475	1202.4921<br>938.4644<br>1729.8635	74<br>58<br>129	R.SQDLHCGACR.A<br>K.TIQMGSFR.I<br>R.INPDGSQSVVEVPYAR.S
37	PREDICTED: glutaredoxin-related protein 5, mitochondrial [Meleagris gallopavo] XP_003206758.3	GLRX5	21	150	23138/8.87	988.4961<br>1278.6601	987.4774<br>1277.6364	56<br>45	R.LHGVEDYR.A<br>R.AHDVLQDPDLR.Q
38	40S ribosomal protein SA, partial [Podiceps cristatus] KFZ67637.1	RPSA	31	189	22782/4.93	1203.6649<br>1698.8602	1202.6408<br>1697.8526	74<br>46	K.FAAATGATPIAGR.F<br>R.FTPGTFTNQIQAAFR.E
39	PREDICTED: 40S ribosomal protein SA-like [Meleagris gallopavo] gi|326922143|ref|XP_003207311.1|	RPSA	21	339	33115/4.80	1740.9610<br>1203.6696<br>1698.8627<br>1306.6543	1739.9417<br>1202.6408<br>1697.8526<br>1305.6387	76<br>106<br>47<br>45	R.AIVAIENPADVSVISSR.N<br>K.FAAATGATPIAGR.F<br>R.FTPGTFTNQIQAAFR.E<br>R.YVDIAIPCNNK.G
40	PREDICTED: nucleophosmin, partial [Meleagris gallopavo] XP_019475889.1	NPM1	25	388	33459/4.90	1583.6787<br>2126.9364<br>1199.6420	1582.7223<br>2126.0168<br>1198.6611	111<br>171<br>76	K.VDDEENEHQLSLR.T<br>K.DELHVVEAEALDYEGNPVK.V<br>K.VIQALWQWR.Q
41	PREDICTED: nucleophosmin, partial [Meleagris gallopavo] XP_019475889.1	NPM1	39	407	33459/4.90	1583.7290<br>2126.9931<br>1199.6818	1582.7223<br>2126.0168<br>1198.6611	111<br>171<br>77	K.VDDEENEHQLSLR.T<br>K.DELHVVEAEALDYEGNPVK.V<br>K.VIQALWQWR.Q
42	PREDICTED: tropomyosin alpha-3 chain-like isoform 3 [Meleagris gallopavo] gi|326915866|ref|XP_003204233.1|	TPM3	26	210	32856/4.68	1243.6280<br>1284.7284	1242.6456<br>1283.7449	85<br>34	R.IQLVEEELDR.A<br>R.KLVIIEGDLER.T

	PREDICTED: tropomyosin beta chain isoform X2 [Melopsittacus undulatus] XP_005152074.1	TPM2	37	391	31938/4.63	1399.8071<br>1243.7059<br>1727.9488<br>940.5026<br>1284.8030	1398.7467<br>1242.6456<br>1726.8849<br>939.4410<br>1283.7449	60<br>85<br>39<br>40<br>54	R.RIQLVEEELDR.A<br>R.IQLVEEELDR.A<br>R.IQLVEEELDRAQER.L<br>K.HIAEEADR.K<br>R.KLVVLEGELER.S
43	PREDICTED: tropomyosin alpha-1 chain isoform X4 [Gallus gallus] XP_015134265.1	TPM1	42	349	32848/4.65	1243.6599<br>940.4431<br>894.4595<br>1284.7557	1242.6456<br>939.4410<br>893.4606<br>1283.7449	94<br>50<br>36<br>61	R.IQLVEEELDR.A<br>K.HIAEEADR.K<br>R.KYEEVAR.K<br>R.KLVIIEGDLER.A
	PREDICTED: tropomyosin alpha-3 chain-like isoform 3 [Meleagris gallopavo] gi|326915866|ref|XP_003204233.1|	TPM3	29	307	32856/4.68	1243.6599<br>940.4431<br>894.4595<br>1284.7557	1242.6456<br>939.4410<br>893.4606<br>1283.7449	94<br>50<br>36<br>59	R.IQLVEEELDR.A<br>K.HIAEEADR.K<br>R.KYEEVAR.K<br>R.KLVIIEGDLER.T
44	cytoskeletal tropomyosin [Coturnix coturnix] CAA38180.1		21	183	28762/4.66	1243.6628<br>940.4520	1242.6456<br>939.4410	51<br>32	R.IQLVEEELDR.A<br>K.HIAEEADR.K
45	PREDICTED: LOW QUALITY PROTEIN: keratin, type I cytoskeletal 19-like [Meleagris gallopavo] XP_003213149.2	KRT19	37	273	45752/4.87	1222.6458<br>1090.4977<br>1379.7229	1221.6353<br>1089.4879<br>1378.7204	44<br>68<br>57	K.TKFETEQALR.M<br>K.DAEAWFHSK.T<br>K.TRLEQEIATYR.Q
46	PREDICTED: LOW QUALITY PROTEIN: keratin, type I cytoskeletal 19-like [Meleagris gallopavo] XP_003213149.2	KRT19	15	151	45752/4.87	1222.6118<br>1090.4645<br>1379.6908	1221.6353<br>1089.4879<br>1378.7204	42<br>49<br>37	K.TKFETEQALR.M<br>K.DAEAWFHSK.T<br>K.TRLEQEIATYR.Q

47	PREDICTED: tubulin beta chain [Hippocampus comes] XP_019751599.1	TUBB	61	654	50070/4.77	1822.9276	1821.9156	122	R.EIVHIQAGQCGNQIGAK.F
						1958.9722	1957.9745	61	K.GHYTEGAELVDSVLDVVR.K
						1271.7323	1270.7220	152	R.KLAVNMVPFPR.L
						1287.7254	1286.7169	117	R.KLAVNMVPFPR.L + Oxidation (M)
						1659.8803	1658.8879	77	R.ALTVPELTQQVFDAK.N
						1696.8412	1695.8257	89	K.NSSYFVEWIPNNVK.T
	PREDICTED: tubulin beta-7 chain-like [Meleagris gallopavo] gi|326930217|ref|XP_003211247.1|	N/A	41	469	43207/4.87	1958.9722	1957.9745	61	K.GHYTEGAELVDSVLDVVR.K
						1271.7323	1270.7220	52	R.KLAVNMVPFPR.L
						1659.8803	1658.8879	77	R.ALTVPELTQQVFDAK.N
						1696.8412	1695.8257	89	K.NSSYFVEWIPNNVK.T
						1229.6079	1228.5910	76	R.ISEQFTAMFR.R
48	PREDICTED: tubulin beta-4A chain [Alligator sinensis] XP_006023414.1	TUBB4A	64	732	50110/4.82	1958.9579	1957.9745	173	K.GHYTEGAELVDSVLDVVR.K
						1077.5323	1076.5250	133	K.IREEYPDR.I
						1130.5943	1129.5880	85	R.FPGQLNADLR.K
						1258.6842	1257.6830	113	R.FPGQLNADLRK.L
						1271.7265	1270.7220	114	R.KLAVNMVPFPR.L
						1143.6293	1142.6270	56	K.LAVNMVPFPR.L
						1696.8201	1695.8257	91	K.NSSYFVEWIPNNVK.T
	PREDICTED: tubulin beta-2 chain-like [Meleagris gallopavo] gi|326917066|ref|XP_003204825.1|	TUBB2A	15	385	53458/4.84	1958.9579	1957.9745	173	K.GHYTEGAELVDSVLDVVR.K
						1077.5323	1076.5250	33	K.IREEYPDR.I
						1130.5943	1129.5880	85	R.FPGQLNADLR.K
						1271.7265	1270.7220	44	R.KLAVNMVPFPR.L
						1143.6293	1142.6270	56	K.LAVNMVPFPR.L
						1696.8201	1695.8257	90	K.NSSYFVEWIPNNVK.T
49	PREDICTED: ATP synthase subunit beta, mitochondrial [Falco cherrug] XP_014132675.1	ATP5F1B	49	886	53445/5.16	1406.6968	1405.6739	131	K.AHGGYSVFAGVGER.T
						2060.9907	2059.9884	139	R.EGNDLYHEMIESGVINLK.D
						1601.8302	1600.8031	115	K.VALVYGQMNEPPGAR.A
						2266.0927	2265.0770	198	R.IPSAVGYQPTLATDMGTMQER.I
50	PREDICTED: protein disulfide-isomerase A6 [Meleagris gallopavo] XP_010706217.1	PDIA6	34	798	49043/5.02	1957.0020	1955.9814	162	K.VGAVDADKHQSLGGQYGVR.G
						1201.6287	1200.6000	89	K.HQSLGGQYGVR.G
						1416.7846	1415.7620	46	R.TSEAIVDAALSALR.S
						1528.7791	1527.7569	126	K.NLEPEWAAAATEVK.E
						1559.8358	1558.8137	73	K.LAAVDATVNQMLASR.Y
						1695.8054	1694.7900	80	K.IFQKGEDPVDYDGGR.T

51	PREDICTED: LOW QUALITY PROTEIN: keratin, type I cyto-skeletal 18 [Meleagris gal-lopavo] XP_019467106.1	KRT18	26	641	48440/5.13	1832.9250<br>1135.5379<br>964.5141<br>1632.8422<br>1002.5077	1831.8813<br>1134.4975<br>963.5025<br>1631.7751<br>1001.4778	175<br>53<br>43<br>174<br>58	R.LTPHSSAASVYAGAGGSGSR.I<br>K.ETMQDLNER.L<br>R.YEAELAIR.T<br>R.SQNAGLEGSLAETEAR.Y<br>R.GEEELAQAR.A
52	PREDICTED: tubulin alpha-1B chain-like [Meleagris gallopavo] gi|326922906|ref|XP_00320768 3.1|	TUBA1B	20	560	50804/4.94	2007.9859<br>1410.8576<br>1757.0521<br>1249.6157<br>1865.0047<br>2330.0965	2006.8858<br>1409.7667<br>1755.9559<br>1248.5453<br>1863.8971<br>2329.0110	160<br>103<br>93<br>58<br>112<br>172	K.TIGGGDDSFNTFFSETGAGK.H<br>R.QLFHPEQLITGK.E<br>R.IHFPLATYAPVISAEK.A<br>K.YMACCLLYR.G<br>R.AVCMLSNTTAIAEAWAR.L<br>R.AFVHWYVGEGMEEGEFSEAR.E
	PREDICTED: tubulin alpha-1D chain-like [Miniopterus na-talensis] XP_016052367.1		57	840	53715/4.89	2007.9859<br>1410.8576<br>1757.0521<br>1249.6157<br>1865.0047<br>2330.0965	2006.8858<br>1409.7667<br>1755.9559<br>1248.5453<br>1863.8971<br>2329.0110	167<br>103<br>93<br>58<br>112<br>172	K.TIGGGDDSFNTFFSETGAGK.H<br>R.QLFHPEQLITGK.E<br>R.IHFPLATYAPVISAEK.A<br>K.YMACCLLYR.G<br>R.AVCMLSNTTAIAEAWAR.L<br>R.AFVHWYVGEGMEEGEFSEAR.E
53	PREDICTED: 78 kDa glucose-regulated protein [Egretta gar-zetta] XP_009645866.1	N/A	42	1300	68092/5.20	1566.7884<br>1430.6924<br>2016.0622<br>1887.9717<br>1815.9912<br>1512.7532<br>1313.6220<br>1588.8658<br>1460.7634<br>1934.0134<br>1974.9084	1565.7726<br>1429.6838<br>2015.0589<br>1886.9639<br>1814.9890<br>1511.7442<br>1312.6122<br>1587.8468<br>1459.7518<br>1933.0058<br>1973.9007	117<br>102<br>65<br>152<br>125<br>115<br>55<br>101<br>98<br>134<br>122	R.ITPSYVAFTPEGER.L<br>R.TWNDPSVQQDIK.Y<br>K.KVTHAVVTVPAYFNDAQR.Q<br>K.VTHAVVTVPAYFNDAQR.Q<br>R.IINEPTAAAIAYGLDKR.E<br>R.AKFEELNMDLFR.S<br>K.FEELNMDLFR.S<br>K.KSDIDEIVLVGGSTR.I<br>K.SDIDEIVLVGGSTR.I<br>K.DNHLLGTFDLTGIPPAPR.G<br>K.IEWLESHQDADIEDFK.A
54	tubulin alpha [Gallus gallus] prf||0703290B		39	708	46259/5.00	2007.8760<br>1410.7798<br>1756.9556<br>1249.5555<br>1864.9045<br>2330.0114	2006.8858<br>1409.7667<br>1755.9559<br>1248.5453<br>1863.8971<br>2329.0110	136<br>94<br>96<br>40<br>124<br>146	-.TIGGGDDSFNTFFSETGAGK.H<br>R.QLFHPEQLITGK.E<br>R.IHFPLATYAPVISAEK.A<br>K.YMACCLLYR.G<br>R.AVCMLSNTTAIAEAWAR.L<br>R.AFVHWYVGEGMEEGEFSEAR.E

55	PREDICTED: actin, cytoplasmic 1 [Coturnix japonica] XP_015732105.1	ACTB	46	347	42161/5.37	976.4454<br>1198.7059<br>1960.9000<br>1515.7607<br>3183.6010<br>1132.5335	975.4410<br>1197.6982<br>1959.9036<br>1514.7419<br>3182.6071<br>1131.5197	36<br>49<br>45<br>82<br>42<br>53	K.AGFAGDDAPR.A<br>R.AVFPSIVGRPR.H<br>K.YPIEHGIITNWDDMEK.I<br>K.IWHHTFYNELR.V<br>R.TTGIVMDSGDGVTHTVPIYEGYAL-PHAILR.L<br>R.GYSFTTTAER.E
56	Actin, alpha cardiac muscle 1, partial [Calypte anna] KFP01962.1	ACTC1	51	405	41169/5.56	976.4410<br>1130.5521<br>2356.1376	975.4410<br>1129.5404<br>2355.1529	60<br>81<br>139	K.AGFAGDDAPR.A<br>R.GYSFVTTAER.E<br>R.KDLYANNVLSGGTTMYPGIADR.M
57	PREDICTED: actin, cytoplasmic 2 [Coturnix japonica] XP_015735544.1	ACTG1	64	574	42121/5.31	976.4427<br>1198.7074<br>1960.9028<br>1515.7631<br>1132.5348	975.4410<br>1197.6982<br>1959.9036<br>1514.7419<br>1131.5197	64<br>50<br>50<br>82<br>59	K.AGFAGDDAPR.A<br>R.AVFPSIVGRPR.H<br>K.YPIEHGIITNWDDMEK.I<br>K.IWHHTFYNELR.V<br>R.GYSFTTTAER.E
58	PREDICTED: actin, cytoplasmic type 5 [Aptenodytes forsteri] XP_009277128.1		57	343	42098/5.30	976.4772<br>1198.7380<br>1171.6128<br>3183.6753	975.4410<br>1197.6982<br>1170.5638<br>3182.6071	47<br>44<br>77<br>76	K.AGFAGDDAPR.A<br>R.AVFPSIVGRPR.H<br>R.HQGVMVGMGQK.D<br>R.TTGIVMDSGDGVTHTVPIYEGYAL-PHAILR.L
59	PREDICTED: zona pellucida sperm-binding protein 1 [Meleagris gallopavo] XP_003206201.2	ZP1	17	543	98702/7.62	1803.6548<br>1419.7207<br>2523.2612<br>1073.5734<br>1358.7767<br>2349.2207	1802.6658<br>1418.7154<br>2522.3129<br>1072.5665<br>1357.7718<br>2348.2641	119<br>102<br>114<br>49<br>44<br>96	R.DACLQAGCCFDDTDR.A<br>R.GLSAQPYNLDSVR.L<br>R.LVYENQLISTIDVQPGPHGSVTR.D<br>R.DSVYILHAR.C<br>K.VLRDPIYVEVR.L<br>R.TQLVPVGPATLQLPFPSHYQR.F
60	PREDICTED: zona pellucida sperm-binding protein 3 [Meleagris gallopavo] XP_010715515.1		15	210	48706/5.58	1590.7381<br>1666.8834	1589.6859<br>1665.8297	80<br>54	R.GDPSAWSYGAEAHSR.A<br>R.TNPAVIPIECHYPR.R
61	PREDICTED: eukaryotic initiation factor 4A-II-like [Meleagris gallopavo] gi|326926084|ref|XP_003209235.1|	EIF4A2	30	415	45743/5.50	1827.9069<br>1198.6655<br>1544.8275<br>1515.7334	1826.9315<br>1197.6717<br>1543.8471<br>1514.7769	136<br>38<br>107<br>60	R.GIYAYGFEKPSAIQQR.A<br>K.ETQALVLAPTR.E<br>K.LQAEAPHIVVGTPGR.V<br>R.GFKDQIYEIFQK.L

62	PREDICTED: 60 kDa heat shock protein, mitochondrial [Meleagris gallopavo] XP_003207490.1	HSPD1	35	865	61108/5/79	1344.7049	1343.7085	106	R.TVIIEQSWGSPK.V
						1373.7002	1372.7027	102	R.GYISPYFINTAK.G
						1601.7371	1600.7443	89	K.CEFQDAYVLISEK.K
						2049.1316	2048.1378	146	K.KISSVQSIVPALEIANSHR.K
						1921.0389	1920.0428	173	K.ISSVQSIVPALEIANSHR.K
63	PREDICTED: heat shock cognate 71 kDa protein isoform X1 [Charadrius vociferus] XP_009889079.1	HSPA8	38	840	71607/5.33	1623.8614	1622.8318	88	K.HWPFTVVNDAGRPK.V
						1982.0141	1980.9905	153	K.TVTNAVVTVPAYFNDSQR.Q
						1691.7412	1690.7183	110	K.STAGDTHLGGEDFDNR.M
						1253.6323	1252.6088	79	R.FEELNADLFR.G
						2774.3340	2773.3195	47	K.QTQTFTTYSDNQPGVLIQVYEGER.A
						1303.6139	1302.5914	76	K.NSLESYAFNMK.A
						1745.8179	1744.8016	103	K.NQTAEKEEFEHQQK.E
64	PREDICTED: heat shock cognate 71 kDa protein isoform X3 [Pterocles gutturalis] XP_010083114.1	HSPA8	29	860	70258/5.37	1623.8497	1622.8318	79	K.HWPFTVVNDAGRPK.V
						1982.0121	1980.9905	160	K.TVTNAVVTVPAYFNDSQR.Q
						1691.7434	1690.7183	132	K.STAGDTHLGGEDFDNR.M
						1253.6291	1252.6088	77	R.FEELNADLFR.G
						2774.3451	2773.3195	129	K.QTQTFTTYSDNQPGVLIQVYEGER.A
						1303.6068	1302.5914	64	K.NSLESYAFNMK.A
						1745.8161	1744.8016	95	K.NQTAEKEEFEHQQK.E
65	PREDICTED: serum albumin [Meleagris gallopavo] XP_010707950.1	ALB	34	635	71740/5.73	1612.7396	1611.7351	76	K.ADPERNECFLSFK.V
						1096.5498	1095.5383	55	K.QQYSCGILK.K
						2330.0052	2329.0393	164	K.TDNPAECYANAQDQLNQHIK.E
						2647.1059	2646.1299	92	R.RQETTPINDNVSHCCSDSYAHR.R
66	PREDICTED: stress-70 protein, mitochondrial-like [Meleagris gallopavo] gi|326928269|ref|XP_003210303.1|	HSPA9	16	215	79550/7.13	1450.7145	1449.7100	66	R.TTPSVVAFTADGER.L
						1694.8309	1693.8424	39	K.NAVITVPAYFNDSQR.Q
						1242.6855	1241.6728	45	K.DAGQISGLNVLR.V
67	PREDICTED: serum albumin [Meleagris gallopavo] XP_010707950.1	ALB	40	278	71740/5.73	1612.7591	1611.7351	65	K.ADPERNECFLSFK.V
						1816.8623	1815.8461	98	K.TNCDLLSAHGEPDFLK.S
68	PREDICTED: stress-70 protein, mitochondrial [Meleagris gallopavo] XP_010717168.1	HSPA9	31	643	66135/5.56	974.4921	974.4921	32	K.VLENSEGAR.T
						1591.7674	1590.7791	87	R.QAVTNPHNTFYATK.R
						1694.8512	1693.8424	119	K.NAVITVPAYFNDSQR.Q
						1242.6856	1241.6728	76	K.DAGQISGLNVLR.V
						1645.8601	1644.8723	95	R.VINEPTAAALAYGLDK.S
						1290.6856	1289.6728	99	K.VQQTVQDLFGR.A

69	PREDICTED: serum albumin [Meleagris gallopavo] XP_010707950.1	ALB	30	321	71740/5.73	1612.7798	1611.7351	68	K.ADPERNECFLSFK.V
						2647.1834	2646.1299	123	R.RQETTPINDNVSHCCSDSYAHR.R
						1032.5198	1031.4706	37	K.LCSAPAEER.E
70	PREDICTED: T-complex protein 1 subunit epsilon [Meleagris gallopavo] XP_010706812.1	CCT5	25	596	56922/5.44	1093.5435	1092.5200	95	R.IADGYEQAAR.I
						1697.8668	1696.8607	96	K.GVIVDKDFSHPQMPK.E
						1610.8984	1609.8902	72	K.IAILTCPFEPPKPK.T
71	PREDICTED: T-complex protein 1 subunit theta isoform X1 [Meleagris gallopavo] XP_010720799.1	CCT5	23	345	60696/5.52	1323.6958	1322.6255	108	K.HYSGLEEAVYR.N
						1150.6424	1149.5818	57	K.FAEAFEAIPR.A
72	PREDICTED: hydroxymethylglutaryl-CoA synthase, cytoplasmic [Meleagris gallopavo] XP_010723686.1	HMGCS2	21	363	50003/5.51	1571.7706	1570.7450	101	M.PGSLPVNTESCWPK.D
						1184.5570	1183.5292	89	R.NSLSYDCIGR.L
						910.4869	909.4821	42	K.IHAQWQK.E
						1529.7231	1528.7045	72	K.LEDTYFDRDVEK.A
73	PREDICTED: keratin, type II cytoskeletal 8 isoform X2 [Meleagris gallopavo] XP_010726169.1	KRT8	39	210	51572/5.31	1197.6367	1196.6190	35	R.GLFEGYVGSLR.R
						1331.6429	1330.6265	44	K.NKYEEEINHR.T
						1079.5286	1078.5043	32	K.AQYEDIANR.S
74	PREDICTED: zona pellucida sperm-binding protein 3 [Meleagris gallopavo] XP_010715515.1	ZP3	31	535	48706/5.58	1590.7208	1589.6859	126	R.GDPSAWSYGAEAHSR.A
						1666.8683	1665.8297	82	R.TNPAVIPIECHYPR.R
						1366.6807	1365.6525	76	K.VTPADQGPDPQNK.A
						1144.5991	1143.5673	81	R.NTWVPVEGSR.D
						2111.8467	2110.8177	114	R.DVCSCCETGNCEPPALSR.R
75	PREDICTED: zona pellucida sperm-binding protein 1 [Meleagris gallopavo] XP_003206201.2	ZP1	9	297	98702/7.62	1803.6895	1802.6658	135	R.DACLQAGCCFDDTDR.A
						1419.7414	1418.7154	112	R.GLSAQPYNLDSVR.L
						1358.7921	1357.7718	36	K.VLRDPIYVEVR.L
76	PREDICTED: farnesyl pyrophosphate synthase [Meleagris gallopavo] XP_010721796.1	FDPS	37	319	29312/6.36	1209.5656	1208.5673	101	K.VDLSQFSEER.Y
						1448.6983	1447.7064	77	K.CSWLVVQCLQR.V
						1324.6795	1323.6857	56	K.VKELYETVGMR.A

77	PREDICTED: LOW QUALITY PROTEIN: 14-3-3 protein epsilon-like [Meleagris gallopavo] gi|326931332|ref|XP_003211786.1|	YWHAE	32	460	26884/4.74	1447.7488	1446.7024	68	K.VAGMDVELTVEER.N
						2180.9738	2179.9328	132	R.DNLTLWTSDMQGDGEEQNK.E
						2196.9763	2195.9277	35	R.DNLTLWTSDMQGDGEEQNK.E
						1819.9711	1818.9298	110	K.AASDIAMTELPPTHPIR.L
						2180.9738	2179.9328	132	R.DNLTLWTSDMQGDGEEQNK.E
78	PREDICTED: 14-3-3 protein epsilon isoform X2 [Haliaeetus albicilla] gi|700339038|ref|XP_009914457.1|	YWHAE	29	274	32321/5.57	1256.5656	1255.5833	77	R.YLAEFATGNDR.K
						1384.6531	1383.6783	40	R.YLAEFATGNDRK.E
						1819.8661	1818.9298	91	K.AASDIAMTELPPTHPIR.L
79	PREDICTED: clathrin light chain B-like [Meleagris gallopavo] gi|326928468|ref|XP_003210400.1|	CLTB	28	213	23097/4.60	947.4233	946.4508	33	K.VTEQEWR.E
						1074.5015	1073.5141	45	K.DLEEWNLR.Q
80	PREDICTED: 14-3-3 protein theta-like [Meleagris gallopavo] gi|326916470|ref|XP_003204530.1|	YWHAQ	26	234	28050/4.68	1532.7214	1531.7114	110	K.AVTEQGAELSNEER.N
						1108.5619	1107.5495	39	K.EMQPTHPIR.L
81	PREDICTED: 14-3-3 protein theta-like [Meleagris gallopavo] gi|326916470|ref|XP_003204530.1|	YWHAQ	27	364	28050/4.68	1532.7394	1531.7114	149	K.AVTEQGAELSNEER.N
						1268.5875	1267.5503	38	R.YLAEVACGDDR.K
						1396.6754	1395.6452	66	R.YLAEVACGDDRK.Q
						1108.5854	1107.5495	50	K.EMQPTHPIR.L
82	PREDICTED: 14-3-3 protein theta-like [Meleagris gallopavo] gi|326916470|ref|XP_003204530.1|	YWHAQ	38	318	28050/4.68	816.4643	815.4137	43	K.LAEQAER.Y
						1532.7880	1531.7114	125	K.AVTEQGAELSNEER.N
						1268.6322	1267.5503	43	R.YLAEVACGDDR.K
						1108.6241	1107.5495	43	K.EMQPTHPIR.L
83	PREDICTED: 14-3-3 protein theta-like [Meleagris gallopavo] gi|326916470|ref|XP_00320453	YWHAQ	38	275	28050/4.78	816.4562	815.4137	30	K.LAEQAER.Y
						1532.7544	1531.7114	89	K.AVTEQGAELSNEER.N
						1108.5901	1107.5495	49	K.EMQPTHPIR.L
84	PREDICTED: zona pellucida sperm-binding protein 3 [Meleagris gallopavo] XP_010715515.1	ZP3	32	720	48706/5.58	1590.6807	1589.6859	119	R.GDPSAWSYGAEAHSR.A
						2456.2481	2455.2754	196	R.AVAGSHPVAVQCQEAQLVVTVHR.D
						1869.9903	1868.9996	151	R.TLINYDPSPASNPVIIR.T
						1666.8262	1665.8297	112	R.TNPAVIPIECHYPR.R

85	PREDICTED: zona pellucida	ZP3	30	483	48706/5.58	1590.7032	1589.6859	123	R.GDPSAWSYGAEAHSR.A
	sperm-binding protein 3 [Mele-					2456.2798	2455.2754	164	R.AVAGSHPVAVQCQEAQLVVTVHR.D
	agris gallopavo]					1870.0130	1868.9996	144	R.TLINYDPSPASNPVIIR.T
86	PREDICTED: zona pellucida	ZP3	29	691	48706/5.58	1590.6763	1589.6859	122	R.GDPSAWSYGAEAHSR.A
	sperm-binding protein 3 [Mele-					2456.2301	2455.2754	176	R.AVAGSHPVAVQCQEAQLVVTVHR.D
	agris gallopavo]					1869.9761	1868.9996	124	R.TLINYDPSPASNPVIIR.T
	XP_010715515.1					1666.8211	1665.8297	100	R.TNPAVIPIECHYPR.R
87	PREDICTED: zona pellucida	ZP3	27	696	48706/5.58	1590.7051	1589.6859	119	R.GDPSAWSYGAEAHSR.A
	sperm-binding protein 3 [Mele-					2456.2587	2455.2754	180	R.AVAGSHPVAVQCQEAQLVVTVHR.D
	agris gallopavo]					1870.0090	1868.9996	157	R.TLINYDPSPASNPVIIR.T
	XP_010715515.1					1666.8458	1665.8297	87	R.TNPAVIPIECHYPR.R
88	PREDICTED: zona pellucida	ZP3	30	867	48706/5.58	1590.7199	1589.6859	126	R.GDPSAWSYGAEAHSR.A
	sperm-binding protein 3 [Mele-					2456.3110	2455.2754	174	R.AVAGSHPVAVQCQEAQLVVTVHR.D
	agris gallopavo]					1870.0443	1868.9996	162	R.TLINYDPSPASNPVIIR.T
	XP_010715515.1					1666.8778	1665.8297	96	R.TNPAVIPIECHYPR.R
						1144.5944	1143.5673	88	R.NTWVPVEGSR.D
89	PREDICTED: zona pellucida	ZP3	29	697	48706/5.58	1590.6458	1589.6859	125	R.GDPSAWSYGAEAHSR.A
	sperm-binding protein 3 [Mele-					2456.1683	2455.2754	165	R.AVAGSHPVAVQCQEAQLVVTVHR.D
	agris gallopavo]					1869.9340	1868.9996	140	R.TLINYDPSPASNPVIIR.T
	XP_010715515.1					1666.7799	1665.8297	84	R.TNPAVIPIECHYPR.R
						1144.5331	1143.5673	68	R.NTWVPVEGSR.D
90	PREDICTED: zona pellucida	ZP3	32	871	48706/5.58	1590.6929	1589.6859	116	R.GDPSAWSYGAEAHSR.A
	sperm-binding protein 3 [Mele-					2456.2394	2455.2754	151	R.AVAGSHPVAVQCQEAQLVVTVHR.D
	agris gallopavo]					1869.9852	1868.9996	139	R.TLINYDPSPASNPVIIR.T
	XP_010715515.1					1666.8323	1665.8297	102	R.TNPAVIPIECHYPR.R
						1366.6584	1365.6525	60	K.VTPADQGPDPQNK.A
						1144.5724	1143.5673	88	R.NTWVPVEGSR.D
						2111.7869	2110.8177	147	R.DVCSCCETGNCEPPALSR.R
91	PREDICTED: zona pellucida	ZP3	32	549	48706/5.58	1590.6458	1589.6859	128	R.GDPSAWSYGAEAHSR.A
	sperm-binding protein 3 [Mele-					2456.1683	2455.2754	46	R.AVAGSHPVAVQCQEAQLVVTVHR.D
	agris gallopavo]					1869.9340	1868.9996	118	R.TLINYDPSPASNPVIIR.T
	XP_010715515.1					1666.7799	1665.8297	105	R.TNPAVIPIECHYPR.R
						1144.5331	1143.5673	86	R.NTWVPVEGSR.D
92	PREDICTED: zona pellucida	ZP3	24	364	48706/5.58	1590.6780	1589.6859	122	R.GDPSAWSYGAEAHSR.A
	sperm-binding protein 3 [Mele-					1869.9797	1868.9996	114	R.TLINYDPSPASNPVIIR.T
	agris gallopavo]					1144.5672	1143.5673	73	R.NTWVPVEGSR.D
	XP_010715515.1								

93	PREDICTED: f-actin-capping protein subunit alpha-1-like [Meleagris gallopavo] gi|326933790|ref|XP_003212982.1|	CAPZA1	37	343	33194/5.78	1197.6918	1196.6877	52	R.LLLNNDNLLR.E
						2124.9237	2123.9735	125	R.EGAAHAFAQYNMDQFTPVK.I
						2028.9669	2028.0065	93	K.IQVHYYEDGNVQLVSHK.D
	PREDICTED: F-actin-capping protein subunit alpha-1, partial [Pelecanus crispus] XP_009491497.1	CAPZA1	43	357	31586/5.93	1197.6918	1196.6877	52	R.LLLNNDNLLR.E
						2124.9237	2123.9735	125	R.EGAAHAFAQYNMDQFTPVK.I
						2028.9669	2028.0065	93	K.IQVHYYEDGNVQLVSHK.D
94	PREDICTED: putative methyl-transferase DDB_G0268948 [Meleagris gallopavo] XP_003207431.2		15	156	30549/5.69	2552.2238	2551.1795	74	K.APSCTQLAVDVGCGSGQGTAFLAER.F
						1002.5503	1001.5182	41	R.EAWDQLLK.Y
						1178.6545	1177.6132	30	R.DTLLEFWVR.Y
95	PREDICTED: annexin A5 [Meleagris gallopavo] XP_010708237.1	ANXA5	26	342	36079/5.57	1268.6019	1267.6197	67	R.GTVTAFSPFDAR.A
						942.4865	941.5083	68	R.IFDAHALR.H
						1759.7232	1758.7770	124	K.QVYMQEYEANLEDK.I
96	PREDICTED: F-actin-capping protein subunit beta isoform X2 [Manacus vitellinus] XP_017941793.1	CAPZB	52	474	33678/6.97	1108.6147	1107.6400	31	R.RLPPQQIEK.N
						1518.6384	1517.6820	85	K.YDPPLEDGAMPSAR.L
						1534.6314	1533.6769	32	K.YDPPLEDGAMPSAR.L + Oxidation (M)
						1696.7649	1695.8216	110	R.KLEVEANNAFDQYR.D
						1568.6816	1567.7267	84	K.LEVEANNAFDQYR.D
						1171.5579	1170.5921	59	R.STLNEIYFGK.T
	PREDICTED: f-actin-capping protein subunit beta isoforms 1 and 2-like [Meleagris gallopavo] gi|326932494|ref|XP_003212351.1|	CAPZB	40	449	33119/5.15	1108.6147	1107.6400	31	R.RLPPQQIEK.N
						1518.6384	1517.6820	85	K.YDPPLEDGAMPSAR.L
						1534.6314	1533.6769	32	K.YDPPLEDGAMPSAR.L Oxidation (M)
						1696.7649	1695.8216	110	R.KLEVEANNAFDQYR.D
						1568.6816	1567.7267	84	K.LEVEANNAFDQYR.D
						1171.5579	1170.5921	59	R.STLNEIYFGK.T
97	Prohibitin, partial [Acanthisitta chloris] KFP76871.1	PHB	52	478	26459/5.40	1185.6310	1184.6513	67	K.DLQNVNITLR.I
						1410.8251	1409.8507	34	R.ILFRPVTAQLPR.I
						1444.6263	1443.6518	97	R.IFTSIGEDYDER.V
						1149.5598	1148.5826	52	R.FDAGELITQR.E
						1998.0092	1997.0793	30	K.AAELIANSLATAGDGLIELR.K
						1606.8055	1605.8362	116	R.KLEAAEDIAYQLSR.S

	PREDICTED: prohibitin-like [Meleagris gallopavo] gi|326934047|ref|XP_00321310 8.1|	PHB	37	230	14952/9.65	1185.6310<br>1410.8251<br>1444.6263	1184.6513<br>1409.8507<br>1443.6518	67<br>34<br>96	K.DLQNVNITLR.I<br>R.ILFRPVTAQLPR.I<br>R.IFTSIGEDYDER.V
98	PREDICTED: NADH dehydro-genase [ubiquinone] iron-sulfur protein 3, mitochondrial [Falco peregrinus] XP_005241954.1	NDUFS3	32	272	23430/5.55	1343.7633<br>909.4406<br>1551.8001<br>924.4597<br>1502.8294	1342.7092<br>908.4140<br>1550.7518<br>923.4348<br>1501.7776	42<br>38<br>44<br>37<br>39	K.SLADLTAVDVPSR.Q<br>K.AANWYER.E<br>R.ILTDYGFEGHPFR.K<br>R.YDDEVKR.V<br>R.VVAEPVELSQEFR.K
99	PREDICTED: peroxiredoxin-4-like [Meleagris gallopavo] gi|326913542|ref|XP_00320309 6.1	PRDX4	26	446	35453/6.34	1355.7286<br>1665.7867<br>1076.6234<br>1243.6537<br>1212.6341	1354.7146<br>1664.7794<br>1075.6026<br>1242.6391<br>1211.6186	79<br>131<br>65<br>50<br>76	K.FTHLAWINTPR.K<br>K.DYGVYLEDQGHTLR.G<br>R.GLFIIDDKR.I<br>R.QITMNDLPVGR.S<br>R.LVQAFQYTDK.H
100	PREDICTED: platelet-activating factor acetylhydrolase IB subu-nit beta-like [Meleagris gal-lopavo] gi|326933420|ref|XP_00321280 2.1|	PAFAH1B2	16	280	25636/5.66	2377.9359<br>2393.9198<br>958.4212	2376.9989<br>2392.9938<br>957.4239	183<br>142<br>43	MSHGDSNPAAVPHAAEDTQGDDR.W<br>MSHGDSNPAAVPHAAEDTQGDDR.W<br>+ Oxidation (M)<br>R.WMSQHNR.F
101	PREDICTED: ubiquitin car-boxyl-terminal hydrolase iso-zyme L1, partial [Meleagris gal-lopavo] XP_010708612.1	UCHL1	37	315	25069/6.35	1649.8193<br>1521.7279<br>929.4612	1648.7944<br>1520.6994<br>928.4477	70<br>109<br>67	K.KFLDETADLSPEER.A<br>K.FLDETADLSPEER.A<br>R.FSAVAFCK.S
102	PREDICTED: protein disulfide-isomerase A3 [Meleagris gal-lopavo] XP_010716010.1	PDIA3	44	660	51211/5.84	1557.7893<br>1752.8397<br>1488.6925<br>1359.6441<br>1664.7379<br>1268.6576	1556.7988<br>1751.8479<br>1487.6715<br>1358.6507<br>1663.7512<br>1267.6601	116<br>108<br>68<br>66<br>93<br>77	K.FIGDKDASVVGFFR.D<br>K.YEEDGEGVVLFRPSR.L<br>K.GDKYVMQEEFSR.D<br>R.FLQDYFDGNLK.K<br>K.MDATANDVPSPYEVR.G<br>R.GFPTIYFAPAGK.K
103	PREDICTED: protein disulfide-isomerase A3 [Meleagris gal-lopavo] XP_010716010.1	PDIA3	31	638	51211/5.84	1698.7863<br>1282.5359<br>1557.8183<br>1752.8691<br>1488.7173<br>1664.7689	1697.7645<br>1281.5109<br>1556.7988<br>1751.8479<br>1487.6715<br>1663.7512	64<br>58<br>76<br>98<br>44<br>106	K.IFRDGEESGTYDGPR.T<br>R.DGEESGTYDGPR.T<br>K.FIGDKDASVVGFFR.D<br>K.YEEDGEGVVLFRPSR.L<br>K.GDKYVMQEEFSR.D<br>K.MDATANDVPSPYEVR.G

104	PREDICTED: cytosolic non-specific dipeptidase-like [Meleagris gallopavo] XP_010706997.1	CNDP2	17	532	53388/5.63	1422.6607	1421.6827	63	K.YIDEHQDLYVK.R
						1189.6027	1188.6139	65	K.TNQEFPVNIK.F
						1221.6273	1220.6336	38	K.NKPCITYGLR.G
						1161.5759	1160.5826	37	K.QFAELQSPNK.F
						1233.6357	1232.6401	45	K.TVFGVEPDLTR.E
						2065.9189	2064.9898	140	K.NVMLLPVGAADDGAHSONEK.L
105	PREDICTED: heterogeneous nuclear ribonucleoprotein K, partial [Acanthisitta chloris] XP_009068800.1	HNRNPK	34	390	34642/8.26	1780.8027	1779.7911	120	R.TDYNASVSVPDSSGPER.I
						1533.7912	1532.7875	57	K.IIPTLEEYQHYK.G
						1098.4614	1097.4448	68	K.GSDFDCELR.L
						1194.7097	1193.6921	80	R.NLPLPPPPPPR.G
106	actin-related protein 3 [Gallus gallus] NP_989638.1	ACTR3	37	634	47847/5.62	1540.7045	1539.7062	113	R.LPACVVDCGTGYTK.L
						1499.6989	1498.6875	31	R.HGIVEDWDLMER.F
						1782.9085	1781.9159	83	R.EREVGIPPEQSLETAK.A
						1228.5877	1227.5772	61	K.EFTIDVGYER.F
						1281.6609	1280.6547	81	K.NIVLSGGSTMFR.D
						1466.6856	1465.6871	73	K.KDYEEIGPSICR.H
						1338.5978	1337.5921	62	K.DYEEIGPSICR.H
	PREDICTED: actin-related protein 3-like [Meleagris gallopavo] gi|326923061|ref|XP_003207760.1|	ACTR3	35	509	46303/5.62	1499.6989	1498.6875	31	R.HGIVEDWDLMER.F
						1782.9085	1781.9159	83	R.EREVGIPPEQSLETAK.A
						1228.5877	1227.5772	61	K.EFTIDVGYER.F
						1281.6609	1280.6547	81	K.NIVLSGGSTMFR.D
						1466.6856	1465.6871	73	K.KDYEEIGPSICR.H
						1338.5978	1337.5921	62	K.DYEEIGPSICR.H
107	PREDICTED: thioredoxin domain-containing protein 5 isoform X2 [Meleagris gallopavo] XP_010706678.1	TXNDC5	42	402	37118/5.61	1856.8105	1855.8081	96	K.VDCTTDTPLCSEFGVR.G
						1266.6648	1265.6517	65	K.THIAEGNHFIK.F
						2023.8494	2022.8524	130	K.VDCTQHYEVCSENQVR.G
108	PREDICTED: serine/threonine-protein phosphatase 2A catalytic subunit alpha isoform-like [Meleagris gallopavo] gi|326918444|ref|XP_003205498.1|	PPP2CA	32	258	37012/5.24	829.4252	828.4242	31	R.GGWGISPR.G
						1915.8324	1914.8254	67	R.AHQLVMEGYNWCHDR.N
						1340.6748	1339.6561	60	K.YSFLQFDPAPR.R

	PREDICTED: serine/threonine-protein phosphatase 2A catalytic subunit beta isoform [Melopsittacus undulatus] XP_005143054.1	PPP2CA	38	311	36081/5.22	1732.7922	1731.7886	41	K.ELDQWIEQLNECR.Q
						829.4252	828.4242	31	R.GGWGISPR.G
						1915.8324	1914.8254	67	R.AHQLVMEGYNWCHDR.N
						1340.6748	1339.6561	60	K.YSFLQFDPAPR.R
109	PREDICTED: eukaryotic translation initiation factor 2 subunit 1 [Meleagris gallopavo] gi|326920582|ref|XP_003206548.1|	EIF2S1	26	281	36441/5.07	981.4739	980.4604	33	R.TAWVFDDK.Y
						1244.6172	1243.5986	51	K.RPGYGAYDAFK.H
						1214.6193	1213.6013	44	R.YVMTTTTLER.T
110	PREDICTED: pyruvate dehydrogenase E1 component subunit beta, mitochondrial-like [Meleagris gallopavo] gi|326928041|ref|XP_003210193.1|	PDHA1	31	404	47133/ 5.91	1915.8977	1914.8919	83	R.DALNQALDEELERDER.V
						1781.9119	1780.9004	53	K.TCYMSAGAIPVPIVFR.G
						1317.6719	1316.6394	58	K.EGVECEVINLR.T
						1753.8593	1752.8505	67	R.IMEGSAFNYLDAPAVR.
						1248.6525	1247.6220	46	R.VTGADVPMPYAK.I
111	PREDICTED: pyruvate dehydrogenase E1 component subunit beta, mitochondrial-like [Meleagris gallopavo] gi|326928041|ref|XP_003210193.1|	PDHB	10	148	47133/5.91	1317.7020	1316.6394	34	K.EGVECEVINLR.T
						1248.6708	1247.6220	46	R.VTGADVPMPYAK.I
112	PREDICTED: alpha-soluble NSF attachment protein [Struthio camelus australis] XP_009673006.1	NAPA	40	423	36108/5.49	1239.5837	1238.5601	42	R.IEEACDIYAR.A
						1577.7360	1576.7270	115	K.HDAATNFVDAGNAFK.K
						2546.1021	2545.1357	76	K.AIAHYEQAADYYKGEESNSSANK.C
						1492.6555	1491.6340	69	K.YEEMFPAFTDSR.E
113	PREDICTED: LOW QUALITY PROTEIN: heat shock-related 70 kDa protein 2 [Meleagris gallopavo] XP_003206814.3	HSPA2	37	742	70083/5.74	1487.6948	1486.6940	82	R.TTPSYVAFTDTER.L
						1663.7902	1662.8035	67	K.NQVAMNPTNTIFDAK.R
						1921.9464	1920.9694	105	K.VQNAVITVPAYFNDSQR.Q
						1691.7060	1690.7183	104	K.STAGDTHLGGEDFDNR.M
						1435.7096	1434.7078	63	R.MVNHFVEEFKR.K
						1253.6154	1252.6088	54	R.FEELNADLFR.G
						1456.8104	1455.8046	71	K.GQIQEIVLVGGSTR.I
						1535.6690	1534.6722	72	R.NQMAEKEEYEHK.Q

114	PREDICTED: annexin A6 isoform X1 [Meleagris gallopavo] XP_003210442.2	ANXA6	33	381	75619/5.63	1073.4706	1072.4825	37	R.STAEYFAER.L
						2001.8907	2000.9552	116	R.GTVQPVGNFNDDGDAQVLR.K
						910.4597	909.4960	45	R.VFQEFIK.M
						1322.5928	1321.6197	50	K.MTNHDVEHAIR.K
						890.4658	889.5022	43	R.DAFVAIVR.S
115	PREDICTED: annexin A6 isoform X1 [Meleagris gallopavo] XP_003210442.2	ANXA6	60	1060	75619/5.63	1707.9080	1706.8839	32	K.GFGSDKDAILDLITSR.S
						1501.7857	1500.7572	101	R.TNQEIHDLVAAYK.D
						1819.8328	1818.8007	90	R.EEDDVVSEDLVEQDAK.D
						2359.0553	2358.0223	181	K.LCGGDDDAAGEFFPEAAQVAYR.M
						2001.9875	2000.9552	143	R.GTVQPVGNFNDDGDAQVLR.K
						2105.0279	2103.9973	164	R.NNQEIAAINEAYQQAYHK.S
						1322.6578	1321.6197	89	K.MTNHDVEHAIR.K
116	PREDICTED: zona pellucida sperm-binding protein 3 [Meleagris gallopavo] XP_010715515.1	ZP3	29	640	48706/5.58	1590.7128	1589.6859	122	R.GDPSAWSYGAEAHSR.A
						2456.2908	2455.2754	170	R.AVAGSHPVAVQCQEAQLVVTVHR.D
						1870.0197	1868.9996	122	R.TLINYDPSPASNPVIIR.T
						1666.8562	1665.8297	85	R.TNPAVIPIECHYPR.R
						1144.5835	1143.5673	74	R.NTWVPVEGSR.D
117	PREDICTED: zona pellucida sperm-binding protein 3 [Meleagris gallopavo] XP_010715515.1	ZP3	29	719	48706/5.58	1590.6943	1589.6859	119	R.GDPSAWSYGAEAHSR.A
						2456.2383	2455.2754	151	R.AVAGSHPVAVQCQEAQLVVTVHR.D
						1869.9937	1868.9996	155	R.TLINYDPSPASNPVIIR.T
						1666.8326	1665.8297	96	R.TNPAVIPIECHYPR.R
						1366.6564	1365.6525	62	K.VTPADQGPDPQNK.A
						1144.5743	1143.5673	82	R.NTWVPVEGSR.D
118	PREDICTED: LOW QUALITY PROTEIN: T-complex protein 1 subunit beta [Merops nubicus] XP_008941120.1	CCT2	17	324	52604/5.88	1419.8279	1418.8146	46	K.KIHPQTIIAGWR.A
						1291.7354	1290.7197	43	K.IHPQTIIAGWR.A
						1458.7157	1457.6899	50	K.AAVDHGDDEVKFR.E
						1130.5626	1129.5451	59	K.HGINCFINR.Q
119	PREDICTED: keratin, type II cytoskeletal cochleal [Meleagris gallopavo] XP_003206045.1	KRT2	40	840	53651/5.97	1112.6140	1111.6350	77	R.SVAVPGVSQIR.V
						1629.7290	1628.7794	161	K.AGGFGSSSLYNLGSANK.R
						1785.8255	1784.8805	58	K.AGGFGSSSLYNLGSANKR.I
						1017.4995	1016.5291	48	K.WSLLQDQK.T
						1079.4788	1078.5043	75	K.AQYEDIANR.S
						1441.6326	1440.6633	60	R.SRAEAESWYQSK.F
						1198.5033	1197.5302	73	R.AEAESWYQSK.F
						1153.5269	1152.5485	71	R.EYQELMNVK.L

120	ATPase inhibitor, mitochondrial, partial [Numida meleagris] XP_021231384.	ATP5IF1	25	248	17283/10.34	1310.5590	1309.5799	48	R.HHEEEIDHHK.K
						1438.6444	1437.6749	88	R.HHEEEIDHHKK.E
121	PREDICTED: 10 kDa heat shock protein, mitochondrial isoform X1 [Meleagris gallopavo] XP_010711708.1	HSPE1	67	508	11476/9.02	1035.5941	1034.5913	62	R.KFLPLFDR.V
						1327.7669	1326.7620	128	K.VLQATVVAVGSGAR.G
						1265.6734	1264.6776	65	R.GKDGEIHPVSVK.V
						1076.5968	1075.5914	59	K.VLLPEYGGTK.I
						1573.8150	1572.8188	117	K.IVLEDKDYYLFR.D
122	PREDICTED: cytochrome c oxidase subunit 6B1 [Meleagris gallopavo] XP_021231384.1	COX6B1	44	210	10545/7.66	1004.4824	1003.4835	34	R.FPNQNQTR.N
						1552.6597	1551.6677	49	R.NCWQNYLDFHR.C
						1487.6294	1486.6299	72	K.GADATPCQWYYR.V
123	ubiquitin-40S ribosomal protein S27a [Meleagris gallopavo] gi|1776497000|ref|XP_031408525.1|	UBB	14	269	23902/9.81	1523.8079	1522.7740	117	K.IQDKEGIPPDQQR.L
						1039.5416	1038.5094	68	K.EGIPPDQQR.L
						1081.5771	1080.5451	40	R.TLSDYNIQK.E
124	40S ribosomal protein S12 [Gallus gallus] NP_001264840.1	RPS12	42	338	15967/8.82	2134.9738	2133.9646	64	R.QAHLCVLASNCDEPMYVK.L
						1751.0071	1749.9447	59	K.LVEALCAEHQINLIK.V
						1061.5640	1060.5376	53	K.LGEWVGLCK.I
						1042.5229	1041.5019	57	K.DVIEEYFK.C
	PREDICTED: 40S ribosomal protein S12-like [Meleagris gallopavo] gi|326915903|ref|XP_003204251.1|	RPS12	42	307	15967/8.82	2135.0292	2133.9646	64	R.QAHLCVLASNCDEPMYVK.L
						1751.0071	1749.9447	59	K.LVEALCAEHQINLIK.V
						1061.6047	1060.5376	53	K.LGEWVGLCK.I
						1042.5688	1041.5019	57	K.DVIEEYFK.C
125	PREDICTED: peptidyl-prolyl cis-trans isomerase B, partial [Meleagris gallopavo] XP_019474804.1	PPIB	47	540	20003/9.12	1378.7023	1377.7140	108	K.TVENFVALATGEK.G
						1626.8173	1625.8236	89	R.VIKDFMIQGGDFTR.G
						1286.5744	1285.5761	105	K.DFMIQGGDFTR.G
						1587.7383	1586.7365	61	K.SIYGDRFPDENFK.L
						1490.6622	1489.6772	83	K.HYGPGWVSMANAGK.D + Oxidation (M)

126	PREDICTED: peptidyl-prolyl cis-trans isomerase B, partial [Meleagris gallopavo] XP_019474804.1	PPIB	46	341	20003/9.12	1286.5933<br>1587.7573<br>1490.6900<br>816.4066<br>1017.5419	1285.5761<br>1586.7365<br>1489.6772<br>815.4178<br>1016.5325	76<br>60<br>62<br>30<br>41	K.DFMIQGGDFTR.G<br>K.SIYGDRFPDENFK.L<br>K.HYGPGWVSMANAGK.D + Oxidation (M)<br>K.TPWLDGK.H<br>K.VLEGMDVVR.K
127	transgelin [Gallus gallus] NP_990825.1	TAGLN	45	197	22357/8.85	1182.5395<br>1337.6504<br>1353.6403	1181.5200<br>1336.6306<br>1352.6255	60<br>51<br>30	K.KYDDELEDR.L<br>K.GASQAGMSYGRPR.Q<br>K.GASQAGMSYGRPR.Q + Oxidation (M)
128	PREDICTED: ATP synthase subunit d, mitochondrial isoform X2 [Pterocles gutturalis] gi|704524989|ref|XP_010084405.1|	ATP5F1D	23	195	15653/9.27	1149.5589<br>1249.6256	1148.5614<br>1248.6350	89<br>79	K.AIDWAAFAER.V<br>R.IAEYEQQLQK.L
129	PREDICTED: LOW QUALITY PROTEIN: cold-inducible RNA-binding protein-like [Meleagris gallopavo] gi|326918654|ref|XP_003205603.1|	CIRBP	39	170	18564/ 9.51	2345.1712<br>1559.7395	2344.1587<br>1558.7304	48<br>72	K.LFVGGLSFDTNEQSLEQVFSK.Y<br>R.GFGFVTFENIDDAK.D
130	PREDICTED: mesencephalic astrocyte-derived neurotrophic factor, partial [Meleagris gallopavo] XP_019475495.1	MANF	40	366	22425/8.82	1533.7774<br>1362.6761<br>1206.5718	1532.7181<br>1361.6398<br>1205.5387	111<br>35<br>95	R.LCYYIGATSDAATK.I<br>R.RILDDWGEACK.G<br>R.ILDDWGEACK.G
131	PREDICTED: LOW QUALITY PROTEIN: cytochrome b-c1 complex subunit 7 [Meleagris gallopavo] XP_010707455.2	UQCRB	29	272	13507/9.24	1233.5727<br>1148.5507<br>1686.8036	1232.5614<br>1147.5509<br>1685.8188	71<br>48<br>89	K.WYYNAAGFNK.Y<br>R.LPEDLYNER.M<br>K.YEEDKPYLEPYLK.E
132	PREDICTED: cytochrome c oxidase subunit 6A1, mitochondrial [Meleagris gallopavo] XP_010718787.1	COX6A1	62	306	9749/9.10	1547.8450<br>1083.5952<br>1246.6460<br>2169.9646	1546.8117<br>1082.5621<br>1245.6142<br>2168.9763	37<br>35<br>76<br>98	1546.8117<br>K.HERPEFIR.Y<br>R.TKPFPWGDGNK.T<br>K.TLFHNPHTNALPTGYEDEN.-
133	PREDICTED: nucleoside diphosphate kinase-like [Meleagris gallopavo]	NME4	51	370	17399/7.79	1344.7445<br>1191.6435<br>1785.8706	1343.7561<br>1190.6448<br>1784.9091	103<br>33<br>106	R.TFIAIKPDGVQR.G<br>K.DRPFYPGLVK.Y<br>R.VMLGETNPADSKPGTIR.G

	gi|326931005|ref|XP_003211627.1|					1051.4852	1050.4917	56	R.GDFCIQVGR.N
134	PREDICTED: peptidyl-prolyl cis-trans isomerase A-like [Meleagris gallopavo] gi|326936164|ref|XP_003214127.1|	PPIA	34	203	11077/8.53	1096.5744	1095.5600	33	K.FADENFILK.H
						1491.7308	1490.7221	68	R.VKEGMNVVEAMER.C
						1264.5789	1263.5587	62	K.EGMNVVEAMER.C
135	PREDICTED: peptidyl-prolyl cis-trans isomerase A-like [Meleagris gallopavo] NP_001159798.1	PPIA	31	245	18084/8.29	1598.7906	1597.7381	36	R.IIPGFMCQGGDFTR.H
						1614.7838	1613.7331	45	R.IIPGFMCQGGDFTR.H + Oxidation (M)
						1096.6206	1095.5600	35	K.FADENFILK.H
						1491.7765	1490.7221	52	R.VKEGMNVVEAMER.C
						1264.6192	1263.5587	54	K.EGMNVVEAMER.C
136	peptidyl-prolyl cis-trans isomerase A [Gallus gallus] NP_001159798.1	PPIA	31	245	18084/8.29	1598.7906	1597.7381	36	R.IIPGFMCQGGDFTR.H
						1614.7838	1613.7331	45	R.IIPGFMCQGGDFTR.H + Oxidation (M)
						1096.6206	1095.5600	35	K.FADENFILK.H
						1491.7765	1490.7221	52	R.VKEGMNVVEAMER.C
						1264.6192	1263.5587	54	K.EGMNVVEAMER.C
137	glyceraldehyde-3-phosphate dehydrogenase [Meleagris gallopavo] NP_001290108.1	GAPDHS	26	509	35908/8.86	1091.4751	1090.4832	63	K.YDSTHGHFK.G
						1819.9060	1818.8968	134	K.IVSNASCTTNCLAPLAK.V
						1556.8118	1555.8029	120	R.VPTPNVSVVDLTCR.L
						1749.8044	1748.7794	118	K.LVSWYDNEFGYSNR.V
138	glyceraldehyde-3-phosphate dehydrogenase [Meleagris gallopavo] NP_001290108.1	GAPDHS	26	490	35908/8.86	1091.5107	1090.4832	60	K.YDSTHGHFK.G
						1819.9492	1818.8968	130	K.IVSNASCTTNCLAPLAK.V
						1556.8372	1555.8029	117	R.VPTPNVSVVDLTCR.L
						1749.8301	1748.7794	122	K.LVSWYDNEFGYSNR.V
139	PREDICTED: malate dehydrogenase, mitochondrial [Meleagris gallopavo] XP_003211747.1	MDH2	47	891	37435/8.83	1487.7626	1486.7490	96	K.GFLGPEQLPECLK.G
						1338.7213	1337.7126	82	K.GCDVVVIPAGVPR.K
						956.4521	955.4624	66	K.HGVYNPNR.I
						1120.6382	1119.6401	81	R.VSVPVIGGHAGK.T
						1370.7808	1369.7639	77	K.TIIPLISQCTPK.V
						1218.5957	1217.5928	70	K.VDFPQDQLEK.L
						1454.7062	1453.6983	101	K.AGAGSATLSMAYAGAR.F
						1195.5778	1194.5703	93	K.EGVIECSFVR.S
						1897.9205	1896.9357	94	R.SEETESPYFSTPLLLGK.N

140	PREDICTED: voltage-dependent anion-selective channel protein 2 [Corvus brachyrhynchos] XP_008641066.1	VDAC2	49	430	30274/8.62	840.4536	839.4541	67	K.GYGFGLVK.L
						1344.6676	1343.6398	106	K.WAEYGLTFTEK.W
						1428.7195	1427.6933	100	K.LTFDTTFSPNTGK.K
						1556.8182	1555.7882	78	K.LTFDTTFSPNTGKK.S
141	PREDICTED: calcyclin-binding protein, partial [Phaethon lepturus] XP_010280426.1	CACYBP	42	238	24936/8.55	1516.7872	1515.7681	52	K.LPAENVQVNFTER.S
						1192.5773	1191.5594	76	K.WECLTQVEK.E
142	glutathione S-transferase alpha class A1.2 [Meleagris gallopavo] NP_001290083.1	GSTA1	31	213	25269/8.75	1578.8188	1577.8314	85	K.VLKDHGHDFLVGNK.L
						1238.5854	1237.5840	94	K.DHGHDFLVGNK.L
143	glutathione S-transferase alpha class A2 [Meleagris gallopavo] gi|356640670|gb|AET31409.1|	GSTA2	19	242	25540/8.45	945.4854	944.4644	45	R.YFPVYEK.A
						1557.8204	1556.7947	91	K.ALKDHGQDYLVGNK.L
						932.5098	931.4876	35	K.FLQPGSQR.K
144	PREDICTED: epididymal secretory protein E1, partial [Meleagris gallopavo] XP_010710061.1		41	413	16360/7.11	812.3488	811.3535	40	R.FVDCGSK.D
						1557.7386	1556.7253	123	K.DGSIQEVNVSPCPR.Q
						1030.5449	1029.5277	61	K.SGIQCPIQK.G
						1068.5172	1067.5036	81	K.GHSYSYLNK.L
145	PREDICTED: phosphatidylethanolamine-binding protein 1 [Meleagris gallopavo] XP_003211138.2	PEBP1	35	316	20406/9.44	1560.8262	1559.8195	71	K.LYTLVLTDPDAPSR.K
						1457.6999	1456.6922	47	R.EWHHFLVTNMK.G + Oxidation (M)
						1324.6959	1323.6863	68	R.YVWLVYEQPK.Q
						1414.7128	1413.7034	79	K.QLACNEPILSNR.S
146	PREDICTED: peroxiredoxin-6, partial [Meleagris gallopavo] XP_010714011.1	PRDX6	40	486	23895/9.32	1395.6542	1394.6500	95	R.DFTPVCTTELGR.A
						1462.6742	1461.6736	57	K.DINAYNGDQPVEK.L
						1149.6625	1148.6594	78	R.VVFIFGPDKK.L
						1191.6726	1190.6659	106	K.LSILYPATTGR.N
						1842.8988	1841.9193	78	K.RGDSVMVVPTLPDEEAK.K
147	phosphoglycerate mutase 1 [Numida meleagris] XP_021255837.1	PGAM1	38	598	29044/7.07	1312.6460	1311.5956	92	R.HGESAWNLENR.F
						2070.9656	2069.8901	153	R.FCGWYDADLSPAGQQEAR.R
						1073.6047	1072.5665	84	R.HYGALTGLNK.A
						1882.9053	1881.8666	107	R.YADLTEDQLPTCESLK.D
						1150.7049	1149.6618	89	R.VLIAAHGNSLR.G

148	triosephosphate isomerase [Numida meleagris] XP_021251610.1	TPI1	48	517	26846/6.71	1082.6038	1081.5709	77	R.KFFVGGNWK.M
						954.4951	953.4760	58	K.FFVGGNWK.M
						1523.8200	1522.7892	117	K.DIGAAWVILGHSER.R
						1614.8480	1613.8162	67	R.RHVFGESDELIGQK.V
						1257.6439	1256.6109	85	K.SHVSDAVAQSTR.I
149	endoplasmic reticulum resident protein 29 precursor [Gallus gallus] NP_001263253.1	ERP29	16	206	28288/7.63	1247.5823	1246.5506	71	K.FDTQYPYGEK.Q
						1703.8916	1702.8526	99	K.ILEQGEEFAANEVVR.I
150	glutathione S-transferase alpha class A1.3 [Meleagris gallopavo] gi|290874550|gb|ADD65347.1|	GSTA1.3	31	236	25399/8.75	1578.8188	1577.8314	85	K.VLKDHGHDFLVGNK.L
						1238.5854	1237.5840	93	K.DHGHDFLVGNK.L
151	PREDICTED: eukaryotic translation initiation factor 4H isoform X1 [Cuculus canorus] gi|696957384|ref|XP_009560101.1|	EIF4H	14	235	37988/10.00	1393.7183	1392.6885	71	K.EALTYDGALLGDR.S
						1669.7198	1668.7049	51	R.GSSMDFREPTEEER.A
	eukaryotic translation initiation factor 4H isoform X2 [Numida meleagris] XP_021271760.1	EIF4H	28	244	25479/8.60	901.4569	900.4090	57	R.AYSSFGGGR.G
						1393.7565	1392.6885	71	K.EALTYDGALLGDR.S
						1669.7677	1668.7049	52	R.GSSMDFREPTEEER.A
152	PREDICTED: endoplasmic reticulum resident protein 29-like [Meleagris gallopavo] gi|326929719|ref|XP_003211004.1|	ERP29	12	210	28179/8.25	1247.5513	1246.5506	53	K.FDTQYPYGEK.Q
						1703.8447	1702.8526	109	K.ILEQGEEFAANEVVR.I
153	PREDICTED: ferritin heavy chain [Meleagris gallopavo] XP_019471620.1	FTH1	43	209	19793/5.83	1152.6129	1151.6047	73	K.QLGDHVTNLR.K
						1236.5543	1235.5532	71	K.YGMAEYLFDK.H
						1252.5498	1251.5482	79	K.YGMAEYLFDK.H + Oxidation (M)

154	PREDICTED: thioredoxin-dependent peroxide reductase, mitochondrial-like [Meleagris gallopavo] gi|733898322|ref|XP_010713240.1|	PRDX3	46	355	25810/7.60	1472.8323<br>1285.7672<br>1220.6970<br>833.4425	1471.8035<br>1284.7442<br>1219.6673<br>832.4290	108<br>48<br>83<br>33	R.DYGVLLEGPGIALR.G<br>R.GLFIIDPNGVIK.H<br>K.HLSINDLPVGR.S<br>R.SVEETLR.L
155	cytochrome c oxidase subunit 5B, mitochondrial [Numida meleagris] XP_021230630.1	COX5B	25	187	14197/8.67	1483.8247<br>955.5746	1482.7678<br>954.5287	80<br>46	K.EDPNLVPSITDKR.I<br>K.LIPHELPH.-
156	PREDICTED: alpha-enolase isoform X1 [Meleagris gallopavo] XP_010721061.1	ENO1	41	531	47651/6.30	1804.9245<br>1143.6063<br>1960.9014<br>1425.7181	1803.9366<br>1142.6084<br>1959.9174<br>1424.7187	117<br>67<br>171<br>102	R.AAVPSGASTGIYEALELR.D<br>R.IGAEVYHNLK.N<br>K.DATNVGDEGGFAPNILENK.E<br>R.YISPDQLADLYK.G
157	Alpha-centractin, partial [Leptosomus discolor] KFQ10545.1	ACTR1A	40	361	41540/6.85	1073.5051<br>965.3310<br>1086.5146<br>1840.8150	1072.5375<br>964.3709<br>1085.5546<br>1839.9003	47<br>43<br>45<br>125	R.YPMEHGIVK.D<br>K.DWNDMER.I<br>R.IWQYVYSK.D<br>K.AQYYLPDGSTIEIGSAR.F
158	PREDICTED: fructose-bisphosphate aldolase C-like [Meleagris gallopavo] gi|326931372|ref|XP_003211805.1|	ALDOC	30	291	39735/6.20	1671.8536<br>1173.6367<br>2231.9554	1670.8336<br>1172.6302<br>2230.9403	37<br>38<br>170	R.LNQIGVENTEENRR.L<br>R.ALQASALSAWR.G<br>K.YEGSGDDSGAAGQSLYVANHAY.-
159	calponin-3 [Gallus gallus] NP_001341600.1	CNN3	26	538	36758/6.14	1107.5852<br>1403.6668<br>1373.6185<br>1216.6416<br>939.4725<br>955.4714<br>1560.6480	1106.5608<br>1402.7238<br>1372.5864<br>1215.6030<br>938.4644<br>954.4593<br>1559.6165	89<br>68<br>78<br>90<br>44<br>38<br>93	K.GPSYGLSAEVK.N<br>K.AGQSVIGLQMGTNK.C<br>K.CASQAGMTAYGTR.R<br>K.GASQAGMLAPGTR.R<br>K.GMSVYGLGR.Q<br>K.GMSVYGLGR.Q + Oxidation (M)<br>R.DYHGQYSDQGIDY.-
	PREDICTED: calponin-3-like [Meleagris gallopavo] gi|326925093|ref|XP_003208756.1|	CNN3	24	435	34871/5.76	1403.6668<br>1373.6185<br>1216.6416<br>939.4725<br>955.4714	1402.7238<br>1372.5864<br>1215.6030<br>938.4644<br>954.4593	68<br>78<br>90<br>44<br>38	K.AGQSVIGLQMGTNK.C<br>K.CASQAGMTAYGTR.R<br>K.GASQAGMLAPGTR.R<br>K.GMSVYGLGR.Q<br>K.GMSVYGLGR.Q Oxidation (M)

						1560.6480	1559.6165	93	R.DYHGQYSDQGIDY.-
160	PREDICTED: S-formylglutathione hydrolase [Meleagris gallopavo] XP_003203382.1	ESD	26	248	32001/6.14	915.4236<br>1386.7249<br>1323.6759	914.3956<br>1385.6939<br>1322.6507	34<br>62<br>94	K.CFEGFQK.V<br>K.LINANFPTDPER.M<br>K.WEAYDATQLVK.S
161	PREDICTED: heterogeneous nuclear ribonucleoprotein H3 isoform X1 [Meleagris gallopavo] XP_010712575.1	HNRNPH3	24	311	34664/6.26	1271.6754<br>3210.2192<br>1241.5539<br>1257.5496	1270.6194<br>3209.1820<br>1240.5143<br>1256.5092	63<br>82<br>77<br>64	R.STGEAFVQFASK.E<br>R.GGGGYDGGYGGFDDYGGYNNYGYG-SDGYDDR.M<br>R.DGMDQGYGSVGR.M<br>R.DGMDQGYGSVGR.M + Oxidation (M)
162	PREDICTED: lysosomal protective protein, partial [Meleagris gallopavo] XP_003212164.2	CTSA	11	248	52312/6.08	1073.4659<br>883.4451<br>1314.5360	1072.4614<br>882.4487<br>1313.5380	64<br>45<br>86	K.YNDYAWNK.I<br>R.LFPEYSK.N<br>K.DLQAFCCSEGK.C
163	Coactosin-like, partial [Cuculus canorus] KFO76261.1	COTL1	41	329	15280/5.29	864.4530<br>1424.6940<br>934.5056<br>1980.0404	863.4501<br>1423.6620<br>933.4920<br>1978.9524	34<br>110<br>32<br>101	R.EAYNLVR.D<br>R.DDATEVNWVTFK.Y<br>K.EVVQNFAK.E<br>K.EFVISDHKELDEDYIK.N
164	serine/arginine-rich splicing factor 1 [Gallus gallus] NP_001107213.1	SRSF1	38	333	27889/10.37	995.5247<br>1123.5207<br>1401.7123<br>1162.6393	994.4356<br>1122.4254<br>1400.6031<br>1161.5414	53<br>45<br>97<br>79	R.DAEDAVYGR.D<br>R.DGYDYDGYR.L<br>R.EAGDVCYADVFR.D<br>R.SHEGETAYIR.V
	PREDICTED: LOW QUALITY PROTEIN: serine/arginine-rich splicing factor 1 [Pelodiscus sinensis] XP_006138677.2	SRSF1	32	337	35919/9.20	995.5247<br>1123.5207<br>1401.7123<br>1162.6393	994.4356<br>1122.4254<br>1400.6031<br>1161.5414	53<br>45<br>97<br>79	R.DAEDAVYGR.D<br>R.DGYDYDGYR.L<br>R.EAGDVCYADVFR.D<br>R.SHEGETAYIR.V
165	PREDICTED: cathepsin B-like [Meleagris gallopavo] gi|326916753|ref|XP_003204669.1|	CTSB	15	300	38520/5.64	1249.6978<br>1881.0359<br>1824.9476	1248.6251<br>1879.9289<br>1823.8472	61<br>156<br>55	K.HYGITSYGVPR.S<br>K.SGVYQHVSGEQVGGHAIR.I<br>R.GEDHCGIESEIVAGVPR.T

166	PREDICTED: L-lactate dehydrogenase B chain isoform X2 [Meleagris gallopavo] XP_010714086.1	LDHB	29	327	36762/6.71	1494.7939	1493.7726	49	K.IVADKDYAVTANSK.I
						1262.6430	1261.6085	48	R.VIGSGCNLDTAR.F
						1292.7097	1291.6772	85	K.QVVESAYEVIR.L
						1148.6222	1147.5873	76	K.SADTLWSIQK.D
167	PREDICTED: annexin A2 [Meleagris gallopavo] XP_019474821.1	ANXA2	51	669	39317/8.04	2011.9157	2010.9646	99	K.AYSNFDADRDAAALEAAIK.T
						1825.8296	1824.8741	120	K.TELEKDIISDTSGDFR.K
						2041.8465	2040.8946	159	R.CEDTSVIDYELIDQDAR.E
						1560.7193	1559.7541	76	K.SYSPYDMLESIKK.E
						1576.7128	1575.7490	52	K.SYSPYDMLESIKK.E + Oxidation (M)
						1405.6661	1404.6925	86	K.SLYYFIQQDTK.G
168	malate dehydrogenase, cytoplasmic isoform MDH1 [Gallus gallus] NP_001006395.1	MDH1	24	192	36748/6.92	1026.4859	1025.4600	31	K.ENFSCLTR.L
						1176.6638	1175.6299	55	K.GDFILTVQQR.G
						1393.7413	1392.7038	73	K.FVEGLPINDFSR.E
	PREDICTED: malate dehydrogenase, cytoplasmic-like [Meleagris gallopavo] gi|326914879|ref|XP_003203750.1|	MDH1	25	182	41370/7.55	1026.4859	1025.4600	31	K.ENFSCLTR.L
						1176.6638	1175.6299	55	K.GDFILTVQQR.G
						1393.7413	1392.7038	73	K.FVEGLPINDFSR.E
169	PREDICTED: heterogeneous nuclear ribonucleoprotein H3 isoform X1 [Meleagris gallopavo] XP_010712575.1	HNRNPH3	43	598	36676/6.60	1271.6135	1270.6194	66	R.STGEAFVQFASK.E
						3210.1109	3209.1820	153	R.GGGGYDGGYGGFDDYGGYNNYGYG-SDGYDDR.M
						1052.5391	1051.5411	51	R.VHIDIGADGR.A
						1241.5117	1240.5143	89	R.DGMDQGYGSVGR.M
						1918.7451	1917.7813	164	R.GSGNSGGYYGQGNMGGGGWR.G
170	PREDICTED: T-complex protein 1 subunit zeta [Meleagris gallopavo] XP_003211780.2	CCT6A	23	579	57064/6.70	1498.7705	1497.7576	112	K.QADLYISEGLHPR.I
						1028.6016	1027.5913	71	K.ALEVLEQVK.V
						937.5314	936.5141	51	R.GLVLDHGAR.H
						1148.5613	1147.5550	82	K.TEVSAGFFYK.S
						918.5356	917.5334	49	K.GFIVINQK.G
171	PREDICTED: LOW QUALITY PROTEIN: elongation factor 1-gamma, partial [Serinus canaria] gi|683941070|ref|XP_009098822.1|	EEF1G	10	169	40955/8.98	821.4833	820.4443	37	R.TFLVGER.V
						1237.7044	1236.6503	70	K.SPFVLDEFKR.K

172	succinyl-CoA:3-ketoacid coenzyme A transferase 1, mitochondrial [Gallus gallus] NP_001006578.2	OXCT1	21	583	45556/5.65	1617.6706	1616.7141	114	R.MVSSYVGENAEFER.Q
						1633.6936	1632.7090	68	R.MVSSYVGENAEFER.Q + Oxidation (M)
						2583.2373	2582.3017	201	R.AGGAGIPAFYTSTGYGTLVQEGGAPIK.Y
						1492.6876	1491.7317	99	K.YNSDGTIAIASQPR.E
173	PREDICTED: aldehyde dehydrogenase, mitochondrial-like [Meleagris gallopavo] gi|326929711|ref|XP_003211000.1|	ALDH2	5	266	95672/6.37	1572.7348	1571.7732	80	K.IFINNEWHDAVSK.K
						1132.5570	1131.5825	77	R.AAFQLGSPWR.R
						1604.6972	1603.7341	85	K.TIPLDGDFFCYTR.H
						1804.7865	1803.8315	111	R.TYVQEDIYNEFVER.S
174	PREDICTED: aldehyde dehydrogenase, mitochondrial-like [Meleagris gallopavo] gi|326929711|ref|XP_00321100	ALDH2	28	538	95672/6.37	1572.7734	1571.7732	92	K.IFINNEWHDAVSK.K
						1132.5931	1131.5825	85	R.AAFQLGSPWR.R
						1604.7408	1603.7341	88	K.TIPLDGDFFCYTR.H
						1804.8334	1803.8315	124	R.TYVQEDIYNEFVER.S
	PREDICTED: aldehyde dehydrogenase, mitochondrial [Par XP_015499536.1us major] XP_015499536.1	ALDH2	28	527	57382/7.51	1572.7734	1571.7732	92	K.IFINNEWHDAVSK.K
						1132.5931	1131.5825	85	K.AAFQLGSPWR.R
						1604.7408	1603.7341	88	K.TIPLDGDFFCYTR.H
						1804.8334	1803.8315	124	R.TYVQEDIYNEFVER.S
						1534.7033	1533.6947	79	K.TEQGPQVDEEQFK.K
175	PREDICTED: ATP synthase subunit alpha, mitochondrial isoform X1 [Melopsittacus undulatus] XP_005145448.1	ATP5F1A	40	525	60155/9.07	1391.7018	1390.6940	31	K.TGTAEVSSILEER.I
						1624.8881	1623.8832	53	R.TGAIVDVPVGEELLGR.V
						1026.5928	1025.5869	49	K.AVDSLVPIGR.G
						1287.6900	1286.6870	80	K.HALIIYDDLSK.Q
						1553.7338	1552.7310	114	R.EAYPGDVFYLHSR.L
						892.4862	891.4814	31	K.LELAQYR.E
176	PREDICTED: ATP synthase subunit alpha, mitochondrial [Meleagris gallopavo] XP_010723474.1	ATP5F1A	27	397	55650/8.54	1391.7209	1390.6940	32	K.TGTAEVSSILEER.I
						1624.9008	1623.8832	64	R.TGAIVDVPVGEELLGR.V
						1287.7139	1286.6870	82	K.HALIIYDDLSK.Q
						1553.7599	1552.7310	73	R.EAYPGDVFYLHSR.L
177	PREDICTED: cytochrome b-c1 complex subunit 2, mitochondrial-like [Meleagris gallopavo] XP_010718246.1	UQCRC2	14	203	48939/6.86	1553.7677	1552.7344	73	K.TALANPLYCPDYR.I
						1217.6901	1216.6564	68	K.QVAEQFLNIR.S

178	PREDICTED: citrate synthase, mitochondrial, partial [Meleagris gallopavo] XP_010725496.2	CS	21	258	54330/7.00	1322.7110	1321.6415	47	R.DFIWNTLNSGR.V
						1167.7326	1166.6560	78	R.VVPGYGHAVLR.K
						1204.6928	1203.6288	35	K.YYTVLFGVSR.A
						1127.7141	1126.6499	32	R.ALGFPLERPK.S
179	PREDICTED: moesin isoform X2 [Meleagris gallopavo] XP_010713423.1	MSN	32	967	67231/5.88	976.5171	975.5389	77	K.QLFDQVVK.T
						1217.5759	1216.5836	36	K.LNKDQWEER.I
						1104.5717	1103.5764	82	K.IGFPWSEIR.N
						1310.6766	1309.6819	77	K.KAPDFVFYAPR.L
						1182.5876	1181.5869	80	K.APDFVFYAPR.L
						1472.7635	1471.7817	44	R.RKPDTIEVQQMK.A
						1387.6750	1386.6851	34	K.AQQELEEQTRR.A
						1661.7761	1660.8090	136	K.TQEQLAAEMAELTAR.I
						943.5295	942.5498	69	R.ITQLELAR.Q
						1031.5320	1030.5407	33	K.ALSSELANAR.D
						1402.5980	1401.6129	101	K.TANDMIHAENMR.L
180	PREDICTED: radixin-like [Meleagris gallopavo] gi|326914349|ref|XP_003203488.1|	RDX	11	203	68556/5.95	1248.5628	1247.5795	59	R.IQNWHEEHR.G
						1182.5717	1181.5869	61	K.APDFVFYAPR.L
181	PREDICTED: vinculin [Meleagris gallopavo] ACT78466.1	VCL	16	527	117476/6.06	1617.7521	1616.7981	69	R.AAQMLQADPYSVPAR.D
						1305.5931	1304.6221	58	R.QQELTHQEHR.V
						1175.5842	1174.6016	32	K.MSAEINEIIR.V
						2019.9109	2018.9922	53	K.GWLRDPNAPPGDAGEQAIR.Q
						1230.5483	1229.5677	61	R.WIDNPTVDDR.G
						1151.5509	1150.5627	33	R.LANVMMGPYR.Q
						2415.0894	2414.2077	56	K.LLAVAATAPSDTPNREEVFEER.A
						944.4129	943.4399	33	K.SFLDSGYR.I
182	PREDICTED: glycogen phosphorylase, liver form [Meleagris gallopavo] XP_010710458.1	PYGL	31	560	98753/6.03	1355.6750	1354.6670	66	R.DYYFALAHTVR.D
						1150.5244	1149.5091	50	R.TQQYYYER.D
						946.4695	945.4668	44	R.APNDFNLR.D
						1442.6892	1441.6878	78	R.VLYPNDNFFEGK.E
						1580.7517	1579.7518	84	R.DFAELEPDKFQNK.T
						1204.5959	1203.5812	63	K.VFADYEAYVK.C
						919.4881	918.4923	59	K.IPPPNEPR.E

183	PREDICTED: programmed cell death 6-interacting protein-like [Meleagris gallopavo] gi|326922173|ref|XP_003207326.1|	PDCD6IP	28	263	92548/6.02	1533.7543	1532.7524	73	K.HYQFASGAFQHIK.D
						1322.6243	1321.6051	34	K.KDNDFIYHDR.V
184	PREDICTED: ezrin [Meleagris gallopavo] XP_010705613.1	EZR	37	732	74449/6.68	976.5275	975.5389	73	K.QLFDQVVK.T
						1031.5515	1030.5560	48	R.KENPLQFR.F
						1535.7543	1534.7715	101	R.IQVWHAEHSGMLK.E
						1104.5792	1103.5764	71	K.IGFPWSEIR.N
						1310.6796	1309.6819	53	K.KAPDFVFYAPR.L
						1182.5910	1181.5869	89	K.APDFVFYAPR.L
185	PREDICTED: moesin isoform X2 [Meleagris gallopavo] XP_010713423.1	MSN	25	353	67231/5.88	976.5316	975.5389	47	K.QLFDQVVK.T
						1310.6912	1309.6819	35	K.KAPDFVFYAPR.L
						1182.5958	1181.5869	67	K.APDFVFYAPR.L
186	PREDICTED: isopentenyl-diphosphate Delta-isomerase 1-like [Meleagris gallopavo] gi|326921556|ref|XP_003207023.1|	IDI2	25	247	36857/6.64	1270.5616	1269.5520	35	K.NCHLNDNIDR.G
						1416.7180	1415.7085	83	R.AFSVFLFNTENK.L
						1081.6378	1080.6219	47	K.LIAETFLFK.W
187	Chain R, Cytochrome Bc1 Complex From Chicken With Fenamidone Bound gi|285803752|pdb|3L75|R		15	166	21751/6.07	1619.8553	1618.7740	88	-.VHNDVTVPDFSAYR.R
						1264.7097	1263.6320	39	K.LRDPQHDLDR.V
	PREDICTED: cytochrome b-c1 complex subunit Rieske, mitochondrial-like [Meleagris gallopavo] XP_003209829.2	UQCRFS1	14	161	27012/6.38	1996.8699	1995.8981	37	R.REDVLDATTSSQSSSEER.K
						1840.7828	1839.7970	31	R.EDVLDATTSSQSSSEER.K
						1264.6300	1263.6320	39	K.LRDPQHDLDR.V
188	PREDICTED: phosphatidylethanolamine-binding protein 1 [Meleagris gallopavo] XP_003211138.2	PEBP1	31	167	20406/9.44	1560.8224	1559.8195	33	K.LYTLVLTDPDAPSR.K
						1324.6955	1323.6863	46	R.YVWLVYEQPK.Q
						1414.7185	1413.7034	47	K.QLACNEPILSNR.S

189	PREDICTED: LOW QUALITY PROTEIN: T-complex protein 1 subunit eta [Cuculus canorus] gi|696994246|ref|XP_009564030.1|	CCT7	27	521	47839/6.58	1533.7256	1532.7333	44	K.KTFSYAGFEMQPK.K
						1405.6366	1404.6384	64	K.TFSYAGFEMQPK.K
						1421.6300	1420.6333	84	K.TFSYAGFEMQPK.K Oxidation (M)
						1565.7864	1564.7886	93	K.LPIGDVATQYFADR.D
						985.5254	984.5240	34	R.VPEEDLKR.T
						1133.5053	1132.5012	61	R.YNFFTGCPK.A
190	PREDICTED: alpha-enolase-like [Meleagris gallopavo] gi|326932384|ref|XP_003212299.1|	ENO1	24	411	47815/6.00	1143.6071	1142.6084	69	R.IGAEVYHNLK.N
						1960.8565	1959.9174	134	K.DATNVGDEGGFAPNILENK.E
						1425.7064	1424.7187	58	R.YISPDQLADLYK.G
191	PREDICTED: glutamine synthetase-like [Meleagris gallopavo] gi|326930228|ref|XP_003211252.1|	ASNS	15	189	43078/6.03	1630.8037	1629.7470	34	K.RHDYHICVYDPR.G
						1517.7237	1516.6583	83	R.QVGQDGFGYFEDR.R
192	PREDICTED: 26S protease regulatory subunit 10B-like [Meleagris gallopavo] gi|326921318|ref|XP_003206908.1|		33	443	42228/6.43	1219.6132	1218.6067	55	K.GCLLYGPPGTGK.T
						930.4129	929.4065	36	R.EMFNYAR.D
						1433.7960	1432.7786	39	R.KIHIDLPNEQAR.L
						1273.6444	1272.6350	91	K.HGEIDYEAIVK.L
						1368.6467	1367.6326	38	R.NVCTEAGMFAIR.A
						1580.7031	1579.6977	94	R.ADHDFVVQEDFMK.A
193	PREDICTED: synaptic vesicle membrane protein VAT-1 homolog [Meleagris gallopavo] XP_003213191.2	VAT1	34	312	53230/9.67	1268.6507	1267.6633	43	R.RGGSPGPGELSVR.V
						1763.6904	1762.7178	126	K.AVCGYDLGNMDEEYK.L
						1062.5768	1061.5869	61	K.LINLYNQGK.I
194	PREDICTED: endoplasmic reticulum resident protein 29-like [Meleagris gallopavo] gi|326929719|ref|XP_003211004.1|	ERP29	14	171	25670/7.66	1247.6232	1246.5506	76	K.FDTQYPYGEK.Q
						1703.9203	1702.8526	66	K.ILEQGEEFAANEVVR.I
195	PREDICTED: serpin H1 [Meleagris gallopavo] XP_010704578.1	SERPINH1	37	553	45681/8.74	1656.7807	1655.8195	75	R.LYGPASINFADDFVK.N
						1205.5526	1204.5625	68	K.KHYNYEHSK.I
						1077.4644	1076.4675	50	K.HYNYEHSK.I
						2087.9991	2087.0535	105	K.SINEWAAQTTDGKLPEVTK.D
						1580.6409	1579.6678	72	K.TNSILFIGR.L

196	PREDICTED: serpin H1 [Meleagris gallopavo] XP_010704578.1	SERPINH1	30	602	45681/8.74	1205.5760	1204.5625	66	K.KHYNYEHSK.I
						1077.4854	1076.4675	58	K.HYNYEHSK.I
						1420.6766	1419.6630	86	K.SINEWAAQTTDGK.L
						2088.0477	2087.0535	112	K.SINEWAAQTTDGKLPEVTK.D
						1580.6851	1579.6678	103	R.TGLYNYYDDEAEK.L
						1020.5974	1019.5764	85	K.TNSILFIGR.L
197	PREDICTED: serpin H1 [Meleagris gallopavo] XP_010704578.1	SERPINH1	17	252	45681/8.74	1205.5548	1204.5625	68	K.KHYNYEHSK.I
						1580.6548	1579.6678	62	R.TGLYNYYDDEAEK.L
						1020.5685	1019.5764	71	K.TNSILFIGR.L
198	PREDICTED: serpin H1-like [Meleagris gallopavo] gi|326914666|ref|XP_003203645.1|	SERPINH1	40	209	45610/ 8.28	2013.0673	2012.0223	43	K.LSSMIFIMPNHVEPLER.V
						1020.6259	1019.5764	54	K.TNSILFIGR.L
199	PREDICTED: elongation factor 1-alpha 1-like [Meleagris gallopavo] gi|326916312|ref|XP_003204452.1|	EEF1A1	8	166	50467/9.10	1404.7183	1403.7197	71	K.YYVTIIDAPGHR.D
						975.5761	974.5437	51	0 R.LPLQDVYK.I
200	PREDICTED: gelsolin isoform X2 [Meleagris gallopavo] XP_010719339.1	GSN	13	324	82630/5.82	2017.9431	2016.9752	94	R.DKDQTEGLGEAYISGHVAK.I
						1314.6870	1313.6728	77	K.VPFDAATLHTSR.A
						1220.6473	1219.6349	89	K.TPSAAYLWVGR.G
201	PREDICTED: annexin A6-like [Meleagris gallopavo] gi|326928554|ref|XP_003210442.1|	ANXA6	60	1160	75619/5.63	1707.9080	1706.8839	35	K.GFGSDKDAILDLITSR.S
						1501.7857	1500.7572	100	R.TNQEIHDLVAAYK.D
						1819.8328	1818.8007	89	R.EEDDVVSEDLVEQDAK.D
						2359.0553	2358.0223	180	K.LCGGDDDAAGEFFPEAAQVAYR.M
						2001.9875	2000.9552	142	R.GTVQPVGNFNDDGDAQVLR.K
						2105.0279	2103.9973	163	R.NNQEIAAINEAYQQAYHK.S
						1322.6578	1321.6197	88	K.MTNHDVEHAIR.K
202	PREDICTED: 26S protease regulatory subunit 7 [Picoides pubescens]	PSMC2	39	430	49030/5.72	1497.7981	1496.7947	70	K.QTLQSEQPLQVAR.C
						1418.7597	1417.7565	49	R.EVVETPLLHPER.F
						1379.5839	1378.5750	111	R.FDDGAGGDNEVQR.T
203	PREDICTED: perilipin-2 isoform X4 [Meleagris gallopavo] XP_010723996.1	PLIN2	12	253	48357/5.75	1928.9475	1927.9680	87	K.VEGFEVEVQKPSYYVR.L
						1238.6725	1237.6131	69	K.LYQSWVEWK.K
						2140.8220	2139.8689	45	K.NTGQNDDDESHSAEHIESR.T

204	PREDICTED: zona pellucida sperm-binding protein 3 [Meleagris gallopavo]<br>XP_010715515.1	ZP3	24	225	48706/5.58	1590.6740<br>1869.9660<br>1666.8132	1589.6859<br>1868.9996<br>1665.8297	79<br>32<br>69	R.GDPSAWSYGAEAHSR.A<br>R.TLINYDPSPASNPVIIR.T<br>R.TNPAVIPIECHYPR.R
205	PREDICTED: thioredoxin domain-containing protein 5 isoform X2 [Meleagris gallopavo]<br>XP_010706678.1	TXNDC5	12	138	37118/5.61	1856.8365<br>2023.8780	1855.8081<br>2022.8524	39<br>82	K.VDCTTDTPLCSEFGVR.G<br>K.VDCTQHYEVCSENQVR.G
206	PREDICTED: cytoskeleton-associated protein 4 [Meleagris gallopavo]<br>XP_019466286.1	CKAP4	39	641	49055/4.81	1724.7799<br>1439.6541<br>1196.5514<br>1122.5197<br>1668.7540<br>1351.6424	1723.8013<br>1438.6576<br>1195.5543<br>1121.5141<br>1667.7638<br>1350.6416	111<br>50<br>60<br>52<br>102<br>126	R.ERDFTSLENTVEER.L<br>R.DFTSLENTVEER.L<br>K.DAFGELQASMK.T<br>K.WEENELFR.K<br>K.ALETFESNSEGLESR.L<br>K.FNSVEGSVDELR.S
207	PREDICTED: neutral alpha-glucosidase AB-like, partial [Gallus gallus]<br>XP_015130042.1	GANAB	13	248	41885/6.28	1830.7548<br>1846.7403<br>1723.6725<br>1043.4638	1829.8367<br>1845.8316<br>1722.7485<br>1042.4760	109<br>54<br>92<br>31	K.VMDYVHGGGEAPQTDVR.W<br>K.VMDYVHGGGEAPQTDVR.W + Oxidation (M)<br>R.WNYQDEEDVAAVER.G<br>R.YFTWDPSK.F
208	PREDICTED: phosphoglycerate kinase 1 [Meleagris gallopavo]<br>XP_010713665.1	PGK1	52	374	41935/7.14	1634.8304<br>1769.0337<br>2010.9908<br>805.4498	1633.7849<br>1767.9883<br>2009.9364<br>804.4494	119<br>58<br>52<br>46	K.LGDVYVNDAFGTAHR.A<br>K.ALESPERPFLAILGGAK.V<br>K.VLNNMQIGNSLFDEEGSK.I+ Oxidation (M)<br>K.FVEVVGR.A
209	PREDICTED: cytochrome b-c1 complex subunit 2, mitochondrial [Meleagris gallopavo]<br>XP_010718246.1	UBB	16	214	48939/6.86	1553.7513<br>2129.0269<br>1217.6727	1552.7344<br>2128.0450<br>1216.6564	59<br>56<br>49	K.TALANPLYCPDYR.I<br>K.ITSEQLHHFVQNNFTSAR.M<br>K.QVAEQFLNIR.S
210	PREDICTED: heterogeneous nuclear ribonucleoprotein A3 homolog 1-like [Meleagris gallopavo]<br>gi|326922673|ref|XP_003207572.1|	HNRNPA3	20	270	39688/8.94	1337.5723<br>1910.7006	1336.6268<br>1909.7827	30<br>181	K.WGTLTDCVVMR.D<br>R.SSGSPYGGGYGSGSGSGGYGGR.R

211	PREDICTED: heterogeneous nuclear ribonucleoprotein A3 isoform X5 [Meleagris gallopavo] XP_010711878.1	HNRNPA3	22	299	40717/9.24	1337.6319<br>1882.9258<br>1910.7686	1336.6268<br>1881.9472<br>1909.7827	38<br>54<br>187	K.WGTLTDCVVMR.D<br>K.IFVGGIKEDTEEYNLR.E<br>R.SSGSPYGGGYGSGSGSGGYGGR.R
212	PREDICTED: acyl-CoA dehydrogenase family member 9, mitochondrial isoform X3 [Meleagris gallopavo] XP_019475592.1	ACAD9	17	309	65192/5.84	1386.6462<br>1239.6031<br>1217.5805	1385.6576<br>1238.5965<br>1216.5659	91<br>63<br>35	R.DFGGVTHGKPEDK.L<br>R.KVDYGIMEER.G<br>R.AYVCSHPLDR.T
213	PREDICTED: mothers against decapentaplegic homolog 2 isoform X2 [Picoides pubescens] XP_009899996.1	SMAD2	33	541	49807/6.67	1114.5881<br>1707.7867<br>1579.7055<br>1415.8693<br>1218.5733<br>838.3685	1113.5753<br>1706.7835<br>1578.6885<br>1414.8548<br>1217.5652<br>837.3769	32<br>76<br>85<br>37<br>70<br>43	K.GLPHVIYCR.L<br>K.KDEVCVNPYHYQR.V<br>K.DEVCVNPYHYQR.V<br>R.VETPVLPPVLVPR.H<br>R.YGWHPATVCK.I<br>K.GWGAEYR.R
	PREDICTED: mothers against decapentaplegic homolog 2 isoform X2 [Pi XP_009899996.1coides pubescens]	SMAD2	33	541	49807/6.67	1114.5881<br>1707.7867<br>1579.7055<br>1415.8693<br>1218.5733	1113.5753<br>1706.7835<br>1578.6885<br>1414.8548<br>1217.5652	32<br>76<br>85<br>37<br>70	K.GLPHVIYCR.L<br>K.KDEVCVNPYHYQR.V<br>K.DEVCVNPYHYQR.V<br>R.VETPVLPPVLVPR.H<br>R.YGWHPATVCK.I
	PREDICTED: mothers against decapentaplegic homolog 3-like, partial [Meleagris gallopavo] gi|326926879|ref|XP_003209624.1|	SMAD3	15	365	46174/6.09	1707.7867<br>1579.7055<br>1415.8693<br>1218.5733<br>838.3685	1706.7835<br>1578.6885<br>1414.8548<br>1217.5652<br>837.3769	76<br>85<br>37<br>70<br>43	K.KDEVCVNPYHYQR.V<br>K.DEVCVNPYHYQR.V<br>R.VETPVLPPVLVPR.H<br>R.YGWHPATVCK.I<br>K.GWGAEYR.R
214	PREDICTED: EH domain-containing protein 3 [Meleagris gallopavo] XP_010713817.1	EHD3	24	441	60915/6.08	1236.6778<br>1332.6815<br>828.4228<br>1509.7641	1235.6411<br>1331.6503<br>827.4501<br>1508.7372	59<br>66<br>30<br>95	K.LNAFGNAFLNR.F<br>K.ADQIETQQLMR.V<br>R.DIQSLPR.N<br>R.EHQISPGDFPNLR.K
215	PREDICTED: NADH-ubiquinone oxidoreductase 75 kDa subunit, mitochondrial-like [Meleagris gallopavo] gi|326922595|ref|XP_003207534.1|	NDUFS1	14	276	80454/6.66	1594.7679<br>1627.7448<br>1064.5625	1593.7635<br>1626.7525<br>1063.5451	49<br>80<br>33	R.FASEVAGVDDLGTTGR.G<br>R.LHEDINEEWISDK.T<br>R.FEAPLFNAR.I

216	PREDICTED: lysyl-tRNA synthetase-like [Meleagris gallopavo] XP_010716411.1		20	202	68807/6.90	959.4135<br>1142.5195	958.4032<br>1141.5226	31<br>47	K.SEEEYFR.I<br>R.FFDDLCAVR.N
217	PREDICTED: 4-trimethylaminobutyraldehyde dehydrogenase [Meleagris gallopavo] XP_003208605.2	ALDH9A1	10	173	54953/6.31	2018.9116<br>966.4831	2017.9229<br>965.4719	107<br>36	R.VEPVDGGQTEDVYEPATGR.V<br>K.AAFQTWSR.M
218	PREDICTED: selenium-binding protein 1-A isoform X2 [Meleagris gallopavo] XP_010722315.1	N/A	14	147	53081/5.89	1296.7014<br>1469.7846	1295.6193<br>1468.7133	30<br>36	K.SPHYCQVIHR.L<br>R.HNVLMSTEWGAPK.V
219	PREDICTED: peptidyl-prolyl cis-trans isomerase FKBP5-like [Meleagris gallopavo] gi|326933538|ref|XP_003212859.1|	FKBP5	21	332	51097/5.82	2087.1049<br>1604.8097<br>1053.6021	2086.0178<br>1603.7300<br>1052.5291	137<br>34<br>70	M.TTDEATKPEGEVQAAALAER.G<br>K.RPGNEDEFPMIGDK.V<br>R.YGFGEAGKPK.Y
220	PREDICTED: cytochrome b-c1 complex subunit 1, mitochondrial [Meleagris gallopavo] XP_010716826.1	UQCRC1	19	265	53290/6.36	2278.0922<br>1290.6564<br>1134.5590	2277.0696<br>1289.6364<br>1133.5353	65<br>36<br>64	R.VASEESSQPTCTVGVWIGAGSR.Y<br>R.RISLEEWDSR.I<br>R.ISLEEWDSR.I
221	PREDICTED: mothers against decapentaplegic homolog 3-like [Meleagris gallopavo] gi|326926879|ref|XP_003209624.1|	SMAD3	7	177	46174/6.09	1707.7704<br>1579.6873<br>1218.5711	1706.7835<br>1578.6885<br>1217.5652	73<br>34<br>47	K.KDEVCVNPYHYQR.V<br>K.DEVCVNPYHYQR.V<br>R.YGWHPATVCK.I
222	PREDICTED: ovotransferrin [Meleagris gallopavo] NP_001290136.1	N/A	11	382	79496/6.82	1671.7730<br>1333.6768<br>2537.0185<br>1047.5357	1670.7511<br>1332.6496<br>2535.9907<br>1046.5185	124<br>36<br>86<br>41	R.NAPYSGYSGAFHCLK.D<br>R.IQWCAVGKDEK.S<br>R.TGSCNFDEYFSEGCAPGSPPDSR.L<br>K.YFGYTGALR.C
223	PREDICTED: ATP-dependent RNA helicase DDX3X isoform X2 [Meleagris gallopavo] XP_010723017.1	DDX3X	17	344	72453/6.54	1270.5468<br>1216.5661<br>2347.0084<br>1089.6051	1269.5374<br>1215.5520<br>2346.0223<br>1088.5978	59<br>91<br>52<br>36	K.QGFDSGGWSSSR.D<br>R.DKDAYSSFGAR.S<br>R.WCDKSDEDDWSKPLPPSER.L<br>R.YTRPTPVQK.H

224	PREDICTED: cytochrome b-c1 complex subunit 2, mitochondrial isoform X2 [Gallus gallus] XP_010725496.2	UQCRC2	23	253	54330/7.00	1167.7177<br>1204.6826	1166.6560<br>1203.6288	81<br>49	R.VVPGYGHAVLR.K<br>K.YYTVLFGVSR.A
225	PREDICTED: cytochrome b-c1 complex subunit 2, mitochondrial isoform X2 [Gallus gallus] XP_004945528.1	UQCRC2	17	176	48649/8.94	1559.8920<br>1217.7385	1558.8103<br>1216.6564	63<br>53	R.YETTANLGTAHLLR.L<br>K.QVAEQFLNIR.S
226	PREDICTED: apolipoprotein A-I [Meleagris gallopavo] XP_010722021.1	APOA1	30	416	30593/5.58	1741.7885<br>1285.6330<br>1351.7479<br>1253.5881<br>1057.6186<br>1120.5891	1740.7856<br>1284.6172<br>1350.7296<br>1252.5724<br>1056.5927<br>1119.5747	59<br>49<br>51<br>75<br>54<br>45	R.SFWQHDDPQTPLDR.I<br>K.LREDMAPYYK.E<br>K.IRPFLDQFSTK.W<br>K.WTEELEQYR.Q<br>K.VVEQLSNLR.E<br>K.MTPLVQEFR.E
227	PREDICTED: fatty acid-binding protein, heart [Meleagris gallopavo] XP_010721582.1	FABP3	25	216	14799/6.12	1445.6332<br>1497.6058	1444.6544<br>1496.6267	62<br>104	K.LVDTANFDEYMK.A<br>K.LGEEFDETTADDR.H
228	PREDICTED: ubiquitin-conjugating enzyme E2 N-like [Meleagris gallopavo] gi|326911690|ref|XP_003202189.1|	UBE2N	11	184	28408/8.69	985.5443<br>1203.6176	984.5029<br>1202.5891	37<br>53	K.IYHPNVDK.L<br>K.TNEAQAIETAR.A
229	PREDICTED: profilin-2 isoform X1 [Meleagris gallopavo] XP_019474798.1	PFN2	17	96	18736/8.89	1434.8053	1433.6899	83	K.SQGGEPTYNVAVGR.A
230	nucleoside diphosphate kinase [Anas platyrhynchos] XP_005016091.1	NME4	60	416	17475/7.72	1344.7863<br>1158.6344<br>1191.6735<br>1785.9400<br>1051.5253	1343.7561<br>1157.6081<br>1190.6448<br>1784.9091<br>1050.4917	100<br>62<br>35<br>68<br>54	R.TFIAIKPDGVQR.G<br>K.FVHASEDLLK.Q<br>K.DRPFYPGLVK.Y<br>R.VMLGETNPADSKPGTIR.G<br>R.GDFCIQVGR.N
231	peptidyl-prolyl cis-trans isomerase A [Gallus gallus] NP_001159798.1	PPIA	33	308	18084/8.29	1598.7896<br>1096.5949<br>1491.7596<br>1264.6012	1597.7381<br>1095.5600<br>1490.7221<br>1263.5587	75<br>72<br>34<br>40	R.IIPGFMCQGGDFTR.H<br>K.FADENFILK.H<br>R.VKEGMNVVEAMER.C<br>K.EGMNVVEAMER.C

232	PREDICTED: mesencephalic astrocyte-derived neurotrophic factor, partial [Meleagris gallopavo] XP_019475495.1	MANF	40	423	22425/8.82	2231.0080	2230.0794	66	R.FYQSLKDDNVEFTPASIEK.E
						1533.6971	1532.7181	108	R.LCYYIGATSDAATK.I
						1973.9682	1973.0404	49	K.IINEVSKPMSHHIPVEK.I + Oxidation (M)
						1206.5143	1205.5387	78	R.ILDDWGEACK.G
233	PREDICTED: NADH dehydrogenase [ubiquinone] 1 alpha subcomplex subunit 9, mitochondrial-like [Meleagris gallopavo] gi|326912578|ref|XP_003202626.1|	NDUFA9	16	144	45665/8.95	1145.6363	1144.6604	33	R.IGSQVIIPYR.C
						962.4859	961.4981	37	K.TYALAGPNR.Y
234	PREDICTED: cholesterol side-chain cleavage enzyme, mitochondrial [Meleagris gallopavo] XP_010715503.1	CYP11A1	18	482	58499/9.25	1540.7122	1539.7252	103	K.EGGFHNVHNIMASK.F
						1133.6704	1132.6717	65	R.LHPVAVTLQR.Y
						1035.5374	1034.5396	38	K.EVILQDYR.I
						1828.8663	1827.8791	106	R.DPEVFPKPEQFNPER.W
						937.4741	936.4818	68	K.GLSFGFGPR.Q
235	PREDICTED: cholesterol side-chain cleavage enzyme, mitochondrial [Meleagris gallopavo] XP_010715503.1	CYP11A1	26	606	58499/9.25	1540.7463	1539.7252	88	K.EGGFHNVHNIMASK.F
						1408.6945	1407.6572	92	R.WTADFSHELFR.F
						1133.7045	1132.6717	71	R.LHPVAVTLQR.Y
						1035.5684	1034.5396	46	K.EVILQDYR.I
						1828.9271	1827.8791	106	R.DPEVFPKPEQFNPER.W
						820.4522	819.4313	44	R.WLVMGSK.H
						937.5156	936.4818	68	K.GLSFGFGPR.Q
236	PREDICTED: trifunctional enzyme subunit beta, mitochondrial isoform X2 [Meleagris gallopavo] XP_003204612.1	HADHB	22	423	51107/9.31	919.5221	918.5287	35	R.LAAAFAISR.V
						1122.5421	1121.5353	64	R.VEQDEYALR.S
						1197.5757	1196.5714	86	R.DFVYVSQDPK.D
						1416.7688	1415.7660	99	K.DQLLLGPTYATPK.V
237	PREDICTED: zona pellucida sperm-binding protein 1 [Meleagris gallopavo] XP_003206201.2	ZP1	7	321	98702/7.62	1803.6641	1802.6658	124	R.DACLQAGCCFDDTDR.A
						1419.7187	1418.7154	82	R.GLSAQPYNLDSVR.L
						1073.5734	1072.5665	54	R.DSVYILHAR.C
						1358.7622	1357.7718	41	K.VLRDPIYVEVR.L
238	PREDICTED: dihydrolipoyl dehydrogenase, mitochondrial-like [Meleagris gallopavo]	DLD	17	402	50607/7.17	1687.9965	1686.8954	115	K.ALLNNSHLYHLAHGK.D
						1127.7330	1126.6499	44	K.ALTGGIAHLFK.Q
						929.5966	928.5131	36	K.VVHVSGFGK.I
						1567.7879	1566.7413	46	K.SEEQLKEEGVEYK.I

	gi|326911238|ref|XP_003201968.1|					1510.7732	1509.7147	77	R.VCHAHPTVSEAFR.E
239	PREDICTED: LOW QUALITY PROTEIN: ornithine aminotransferase, mitochondrial [Meleagris gallopavo] XP_003208269.1	OAT	16	331	48811/7.12	1609.7563<br>1736.8460<br>1179.5870<br>1251.6563	1608.7784<br>1735.8794<br>1178.5720<br>1250.6560	86<br>70<br>77<br>57	K.TIQGPPSSDYIFER.E<br>K.YGAHNYHPLPVALER.G<br>K.GVYVWDVEGR.K<br>K.IIFAAGNFWGR.T
240	PREDICTED: aflatoxin B1 aldehyde reductase member 2-like [Meleagris gallopavo] XP_019477922.1	AKR7A2	19	372	35769/6.80	1649.7539<br>1374.6323<br>1593.7970<br>970.4923<br>1393.6360	1648.7515<br>1373.6153<br>1592.7794<br>969.4821<br>1392.6245	77<br>70<br>74<br>43<br>54	R.LLDTAHMYAGGESER.I<br>R.FFGNDWAQAYR.D<br>K.DAYGSNAPSLTSAALR.W<br>R.WLYHHSK.L<br>K.LTAHDCPNYFR.-
241	PREDICTED: guanine nucleotide-binding protein subunit beta-2-like 1-like [Meleagris gallopavo] gi|326930102|ref|XP_003211191.1|	GNB2	44	388	35511/7.60	1192.5692<br>1264.6557<br>1366.6727<br>860.4493	1191.5520<br>1263.6459<br>1365.6751<br>859.4440	86<br>87<br>75<br>30	R.DETNYGIPQR.A<br>R.LWDLTTGTTTR.R<br>R.YWLCAATGPSIK.I<br>K.IWDLEGK.I
242	PREDICTED: heterogeneous nuclear ribonucleoproteins A2/B1-like [Meleagris gallopavo] gi|326921905|ref|XP_003207194.1|	HNRNPA2B1	38	415	38451/9.33	1087.4684<br>1879.9271<br>1050.4321<br>1695.7315<br>1377.6224	1086.4770<br>1878.9588<br>1049.4342<br>1694.7577<br>1376.6222	38<br>42<br>58<br>92<br>76	R.NYYEQWGK.L<br>K.LFVGGIKEDTEEHHLR.D<br>R.DYFEEYGK.I<br>R.GFGFVTFDDHDPVDK.I<br>R.GGGGNFGPGPGSNFR.G
243	PREDICTED: heterogeneous nuclear ribonucleoproteins A2/B1-like [Meleagris gallopavo] gi|326921905|ref|XP_003207194.1|	HNRNPA2B1	25	198	38451/9.33	1087.5138<br>997.4808<br>1377.6696	1086.4770<br>996.4414<br>1376.6222	32<br>50<br>53	R.NYYEQWGK.L<br>R.GGNFGFGDAR.G<br>R.GGGGNFGPGPGSNFR.G
	PREDICTED: heterogeneous nuclear ribonucleoproteins A2/B1-like [Meleagris gallopavo]	HNRNPA2B1	25	198	38451/9.33	1087.5138<br>997.4808<br>1377.6696	1086.4770<br>996.4414<br>1376.6222	32<br>50<br>53	R.NYYEQWGK.L<br>R.GGNFGFGDAR.G<br>R.GGGGNFGPGPGSNFR.G

	gi|326921905|ref|XP_003207194.1|								
244	PREDICTED: apolipoprotein B-100 [Meleagris gallopavo] XP_010706280.1	APOB	3	121	525494/8.32	2190.9780	2189.9515	40	K.WTSPNFSDEGTHNSHATFR.V
						1337.6792	1336.6411	45	K.DYSLWEQAGLR.D
245	PREDICTED: apolipoprotein B-100 [Meleagris gallopavo] XP_010706280.1	APOB	2	443	525494/8.32	985.5338	984.5393	67	K.IPDFQLPR.I
						1268.6758	1267.6925	99	R.IPHTVTAPTFGK.L
						1378.6264	1377.6459	45	K.IQMQSGTAYSHR.L
						2190.8903	2189.9515	71	K.WTSPNFSDEGTHNSHATFR.V
						1337.6343	1336.6411	96	K.DYSLWEQAGLR.D
						944.5108	943.5167	43	K.YIIFFNK.Y
246	PREDICTED: U1 small nuclear ribonucleoprotein 70 kDa-like [Thamnophis sirtalis] XP_013921068.1	SNRNP70	18	148	23010/7.92	1652.8363	1651.8219	45	K.HHNQPYSGIAPYIR.E
						1413.6557	1412.6360	45	R.GYAFIEYEHER.D
247	PREDICTED: zona pellucida sperm-binding protein 1 [Meleagris gallopavo] XP_003206201.2	ZP1	17	343	98702/7.62	1803.5793	1802.6658	115	R.DACLQAGCCFDDTDR.A
						1419.6667	1418.7154	100	R.GLSAQPYNLDSVR.L
						2300.9192	2300.0485	93	R.IATDESYSSYHPDTDYPLVK.V
248	zona pellucida sperm-binding protein 1 precursor [Meleagris gallopavo] NP_001290145.1	ZP1	15	332	102104/8.46	1803.6707	1802.6658	121	R.DACLQAGCCFDDTDR.A
						1419.7363	1418.7154	77	R.GLSAQPYNLDSVR.L
						1073.5827	1072.5665	57	R.DSVYILHAR.C
						1358.7868	1357.7718	49	K.VLRDPIYVEVR.L
249	zona pellucida sperm-binding protein 1 precursor [Meleagris gallopavo] NP_001290145.1	ZP1	15	328	102104/8.46	1803.6495	2189.9663	30	R.YHYDCGDFGMQLLAFPTR.G
						1803.6495	1802.6658	142	R.DACLQAGCCFDDTDR.A
						1419.7089	1418.7154	89	R.GLSAQPYNLDSVR.L
						1358.7643	1357.7718	47	K.VLRDPIYVEVR.L
250	PREDICTED: zona pellucida sperm-binding protein 1 [Meleagris gallopavo] XP_003206201.2	ZP1	20	830	98702/7.62	2190.9709	2189.9663	143	R.YHYDCGDFGMQLLAFPTR.G
						936.4786	935.4713	38	K.VLDEFGTR.F
						1803.6890	1802.6658	145	R.DACLQAGCCFDDTDR.A
						1419.7403	1418.7154	84	R.GLSAQPYNLDSVR.L
						2523.3285	2522.3129	146	R.LVYENQLISTIDVQPGPHGSVTR.D
						1073.5939	1072.5665	72	R.DSVYILHAR.C

						1358.7974	1357.7718	55	K.VLRDPIYVEVR.L
						2349.2745	2348.2641	79	R.TQLVPVGPATLQLPFPSHYQR.F
	zona pellucida sperm-binding protein 1 precursor [Meleagris gallopavo] NP_001290145.1	ZP1	15	666	102104/8.46	2190.9709	2189.9663	143	R.YHYDCGDFGMQLLAFPTR.G
						936.4786	935.4713	38	K.VLDEFGTR.F
						1803.6890	1802.6658	145	R.DACLQAGCCFDDTDR.A
						1419.7403	1418.7154	84	R.GLSAQPYNLDSVR.L
						1073.5939	1072.5665	72	R.DSVYILHAR.C
						1358.7974	1357.7718	55	K.VLRDPIYVEVR.L
251	zona pellucida sperm-binding protein 1 precursor [Meleagris gallopavo] NP_001290145.1	ZP1	15	360	102104/8.46	1488.5975	1487.5960	56	R.DENMHGHHQPDR.G + Oxidation (M)
						1803.6615	1802.6658	142	R.DACLQAGCCFDDTDR.A
						1419.7214	1418.7154	96	R.GLSAQPYNLDSVR.L
						1358.7688	1357.7718	50	K.VLRDPIYVEVR.L
252	zona pellucida sperm-binding protein 1 precursor [Meleagris gallopavo] NP_001290145.1	ZP1	15	353	102104/8.46	1488.5838	1487.5960	53	R.DENMHGHHQPDR.G + Oxidation (M)
						1803.6427	1802.6658	139	R.DACLQAGCCFDDTDR.A
						1419.7104	1418.7154	99	R.GLSAQPYNLDSVR.L
						1358.7571	1357.7718	47	K.VLRDPIYVEVR.L
253	PREDICTED: zona pellucida sperm-binding protein 1 [Meleagris gallopavo] XP_003206201.2	ZP1	10	280	98702/7.62	1803.6346	1802.6658	135	R.DACLQAGCCFDDTDR.A
						1419.7058	1418.7154	105	R.GLSAQPYNLDSVR.L
254	PREDICTED: zona pellucida sperm-binding protein 1 [Meleagris gallopavo] XP_003206201.2	ZP1	13	482	98702/7.62	1488.5858	1487.5960	45	R.DENMHGHHQPDR.G + Oxidation (M)
						1803.6367	1802.6658	127	R.DACLQAGCCFDDTDR.A
						1419.7034	1418.7154	102	R.GLSAQPYNLDSVR.L
						1073.5626	1072.5665	89	R.DSVYILHAR.C
						1358.7598	1357.7718	52	K.VLRDPIYVEVR.L
255	PREDICTED: zona pellucida sperm-binding protein 1 [Meleagris gallopavo] [illegible]	ZP1	10	331	98702/7.62	1488.6248	1487.5960	40	R.DENMHGHHQPDR.G + Oxidation (M)
						1803.6684	1802.6658	131	R.DACLQAGCCFDDTDR.A
						1419.7277	1418.7154	101	R.GLSAQPYNLDSVR.L
						[illegible]	[illegible]	[illegible]	[illegible]
256	PREDICTED: zona pellucida sperm-binding protein 1 [Meleagris gallopavo] XP_003206201.2	ZP1	10	390	98702/7.62	936.4677	935.4713	33	K.VLDEFGTR.F
						1488.5854	1487.5960	50	R.DENMHGHHQPDR.G + Oxidation (M)
						1803.6444	1802.6658	124	R.DACLQAGCCFDDTDR.A
						1419.7084	1418.7154	85	R.GLSAQPYNLDSVR.L
						1073.5670	1072.5665	50	R.DSVYILHAR.C

257	PREDICTED: zona pellucida sperm-binding protein 1 [Meleagris gallopavo]<br>[illegible]	ZP1	9	241	98702/7.62	1803.6475<br>1419.7092<br>1073.5612	1802.6658<br>1418.7154<br>1072.5665	75<br>57<br>47	R.DACLQAGCCFDDTDR.A<br>R.GLSAQPYNLDSVR.L<br>R.DSVYILHAR.C
258	PREDICTED: zona pellucida sperm-binding protein 1 [Meleagris gallopavo]	ZP1	10	237	98702/7.62	1803.7904<br>1419.8338<br>2301.1708	1802.6658<br>1418.7154<br>2300.0485	54<br>52<br>65	R.DACLQAGCCFDDTDR.A<br>R.GLSAQPYNLDSVR.L<br>R.IATDESYSSYHPDTDYPLVK.V
259	PREDICTED: LOW QUALITY PROTEIN: zona pellucida sperm-binding protein 1 [Meleagris gallopavo]<br>gi|326919872|ref|XP_00320620	ZP1	16	456	96950/8.00	1803.6418<br>1419.7069<br>2523.3058<br>1073.5716<br>1358.7634	1802.6658<br>1418.7154<br>2522.3129<br>1072.5665<br>1357.7718	110<br>91<br>60<br>65<br>39	R.DACLQAGCCFDDTDR.A<br>R.GLSAQPYNLDSVR.L<br>R.LVYENQLISTIDVQPGPHGSVTR.D<br>R.DSVYILHAR.C<br>K.VLRDPIYVEVR.L
260	PREDICTED: zona pellucida sperm-binding protein 1 [Meleagris gallopavo]<br>XP_003206201.2	ZP1	16	442	98702/7.62	1803.6802<br>1419.7311<br>2523.3105<br>1073.5837	1802.6658<br>1418.7154<br>2522.3129<br>1072.5665	118<br>87<br>68<br>36	R.DACLQAGCCFDDTDR.A<br>R.GLSAQPYNLDSVR.L<br>R.LVYENQLISTIDVQPGPHGSVTR.D<br>R.DSVYILHAR.C
261	PREDICTED: elongation factor 1-alpha 1-like [Meleagris gallopavo]<br>gi|326916312|ref|XP_00320445	EEF1A1	8	199	50467/9.10	1404.7133<br>975.5383<br>1025.6039	1403.7197<br>974.5437<br>1024.6030	85<br>71<br>30	K.YYVTIIDAPGHR.D<br>R.LPLQDVYK.I<br>K.IGGIGTVPVGR.V
262	PREDICTED: elongation factor 1-alpha 1 [Pseudopodoces humilis]<br>XP_005518198.1	EEF1A1	17	223	50468/9.10	1404.7562<br>1025.6357<br>3009.4372<br>3025.4371<br>3041.4320	1403.7197<br>1024.6030<br>3008.4082<br>3024.4031<br>3040.3980	82<br>33<br>89<br>63<br>39	K.YYVTIIDAPGHR.D<br>K.IGGIGTVPVGR.V<br>K.SGDAAIVDMIPGKPMCVESFSDYPPLGR.[illegible]<br>K.SGDAAIVDMIPGKPMCVESFSDYPPLGR.[illegible]<br>+ Oxidation (M)<br>K.SGDAAIVDMIPGKPMCVESFSDYPPLGR.[illegible]<br>+ 2 Oxidation (M)
263	PREDICTED: elongation factor 1-alpha 1-like [Meleagris gallopavo]<br>gi|326916312|ref|XP_00320445	EEF1A1	8	179	50467/9.10	1404.7753<br>975.5823<br>1025.6491	1403.7197<br>974.5437<br>1024.6030	80<br>41<br>47	K.YYVTIIDAPGHR.D<br>R.LPLQDVYK.I<br>K.IGGIGTVPVGR.V
264	PREDICTED: aspartate aminotransferase, mitochondrial [Meleagris gallopavo]<br>XP_003209725.3	GOT2	22	362	48223/9.15	1437.7524<br>918.4944<br>1134.5812<br>939.4206<br>994.4978	1436.7624<br>917.5083<br>1133.5829<br>938.4280<br>993.5066	84<br>37<br>71<br>32<br>48	R.YVTVQGISGTGSLR.I<br>R.IGANFLQR.F<br>R.DAGLQLQAYR.Y<br>K.NMGLYGER.A<br>R.AGAFTVICR.D

265	PREDICTED: aspartate aminotransferase, mitochondrial [Meleagris gallopavo]	GOT2	14	235	48223/9.15	1437.7711	1436.7624	43	R.YVTVQGISGTGSLR.I
						918.4942	917.5083	38	R.IGANFLQR.F
						1134.5806	1133.5829	60	R.DAGLQLQAYR.Y
	[illegible]					[illegible]	[illegible]	[illegible]	[illegible]
266	PREDICTED: polyadenylate-binding protein 1-like [Meleagris gallopavo]	PABPN1	24	228	72992/ 9.58	1662.7354	1661.7396	55	K.GFGFVCFSSPEEATK.A
						1542.9250	1541.9293	71	R.IVATKPLYVALAQR.K
267	PREDICTED: trifunctional enzyme subunit alpha, mitochondrial-like [Meleagris gallopavo] gi|326916634|ref|XP_003204611.1|	HADHA	23	209	83519/9.20	2063.1623	2062.1786	36	K.TILGTPEVLLGLLPGAGGTQR.L
						1226.6984	1225.6931	38	K.ALIGLYHGQVR.C
						1901.8853	1900.9023	30	K.LPAKPEVCTDEDIQMR.L
268	PREDICTED: heat shock protein beta-1 [Meleagris gallopavo] XP_003211746.1	HSPB1	32	388	21886/6.23	920.3991	919.3937	48	R.DWYHGSR.L
						1611.7372	1610.7437	85	K.HEEKQDEHGFISR.C
						1088.5177	1087.5047	43	K.QDEHGFISR.C
						1501.8282	1500.8300	60	R.KYTLPPGVEATAVR.S
						1373.7335	1372.7351	45	K.YTLPPGVEATAVR.S
269	PREDICTED: elongation factor 1-alpha 1-like [Meleagris gallopavo] gi|326916312|ref|XP_003204452.1|	EEF1A1	30	199	50467/ 9.10	1404.7298	1403.7197	43	K.YYVTIIDAPGHR.D
						975.5533	974.5437	52	R.LPLQDVYK.I
270	PREDICTED: plasminogen activator inhibitor 1 RNA-binding protein isoform X3 [Coturnix japonica] XP_015726260.1	SERBP1	20	538	46147/7.89	1670.7922	1669.7995	96	M.PGHLQEGFGCVVTNR.F
						1511.7819	1510.7892	117	R.KNPLPPFAASAGGER.R
						2007.9630	2006.9882	111	R.RPDQQQQQQGEGKPIDR.R
						1229.5790	1228.5836	79	R.KPNEGADGQWK.K
						2111.8747	2110.9126	51	K.SKSEEAHAEDSVMDHHFR.K
271	stress-induced-phosphoprotein 1, partial [Numida meleagris] XP_021238872.1	STIP1	12	92	22485/5.51	1002.5351	1001.5043	38	R.NHVLYSNR.S
						1912.9543	1911.9438	40	K.ATYEEGLKHEPGNAQLR.E
272	PREDICTED: 4-trimethylaminobutyraldehyde dehydrogenase [Meleagris gallopavo] XP_003208605.2	ALDH9A1	16	379	54953/6.31	2018.9022	2017.9229	161	R.VEPVDGGQTEDVYEPATGR.V
						966.4749	965.4719	38	K.AAFQTWSR.M
						1024.5555	1023.5502	37	K.FGLAGGVFTR.D
						1185.6102	1184.6077	81	R.AAIEYYSQLK.T

273	PREDICTED: lysosomal protective protein, partial [Meleagris gallopavo] XP_003212164.2	CTSA	17	394	52312/6.08	1509.7048	1508.7307	61	R.HFSGHLCIGPTQR.L
						1636.8027	1635.8621	85	K.EHGPFLVQPDGVTLK.Y
						1998.8730	1997.9483	85	R.YEGDYLVTHDLGNSFIR.M
						1446.6440	1445.6683	72	R.LYMQMNDQYLK.L
274	PREDICTED: cholesterol side-chain cleavage enzyme, mitochondrial [Meleagris gallopavo] XP_010715503.1	CYP11A1	21	399	58499/9.25	1408.6748	1407.6572	62	R.WTADFSHELFR.F
						1133.6946	1132.6717	56	R.LHPVAVTLQR.Y
						1035.5546	1034.5396	38	K.EVILQDYR.I
						1828.8891	1827.8791	106	R.DPEVFPKPEQFNPER.W
						937.4979	936.4818	67	K.GLSFGFGPR.Q
275	PREDICTED: cholesterol side-chain cleavage enzyme, mitochondrial [Meleagris gallopavo] XP_010715503.1	CYP11A1	31	829	58499/9.25	1540.7295	1539.7252	85	K.EGGFHNVHNIMASK.F
						1446.7926	1445.7878	77	K.LGVYESVNIISPR.D
						1221.6562	1220.6342	61	R.FSVPPWVAYR.D
						1408.6757	1407.6572	86	R.WTADFSHELFR.F
						1679.8341	1678.8137	96	R.FALESVCHVLYGER.L
						1753.8327	1752.8220	133	R.DHVHAWDAIFTQADK.C
						1133.6779	1132.6717	50	R.LHPVAVTLQR.Y
						1035.5482	1034.5396	36	K.EVILQDYR.I
						1828.9030	1827.8791	125	R.DPEVFPKPEQFNPER.W
276	PREDICTED: cholesterol side-chain cleavage enzyme, mitochondrial [Meleagris gallopavo] XP_010715503.1	CYP11A1	29	288	58499/9.25	1133.7215	1132.6717	33	R.LHPVAVTLQR.Y
						1828.9349	1827.8791	106	R.DPEVFPKPEQFNPER.W
						937.5321	936.4818	43	K.GLSFGFGPR.Q
277	PREDICTED: mitogen-activated protein kinase 1-like [Meleagris gallopavo] gi|326929501|ref|XP_003210902.1|	MAPK1	22	347	42701/6.81	1565.7161	1564.7093	71	K.ISPFEHQTYCQR.T
						1209.6529	1208.6414	71	K.YIHSANVLHR.D
						2143.9770	2142.9971	56	R.VADPDHDHTGFLTEYVATR.W
						913.4674	912.4930	40	K.NKVPWNR.L
						1107.5701	1106.5608	46	K.ELIFEETAR.F
278	PREDICTED: zona pellucida sperm-binding protein 3 [Meleagris gallopavo]	ZP3	15	236	48706/5.58	1590.6788	1589.6859	67	R.GDPSAWSYGAEAHSR.A
						1666.8212	1665.8297	42	R.TNPAVIPIECHYPR.R
						2111.7917	2110.8177	73	R.DVCSCCETGNCEPPALSR.R
279	PREDICTED: zona pellucida sperm-binding protein 3 [Meleagris gallopavo] XP_010715515.1	ZP3	23	162	48706/5.58	1590.6713	1589.6859	48	R.GDPSAWSYGAEAHSR.A
						1666.8167	1665.8297	37	R.TNPAVIPIECHYPR.R

280	PREDICTED: collagen alpha-2(VI) chain isoform X1 [Meleagris gallopavo] XP_003207399.1	COL6A2	15	354	110229/5.55	1793.8546<br>1297.6274<br>1425.6639<br>1033.5370<br>1000.4638<br>1847.7881	1792.8996<br>1296.6462<br>1424.6895<br>1032.5716<br>999.4886<br>1846.8373	76<br>35<br>60<br>32<br>36<br>58	K.LFAVAPSEDVYEQGLR.E<br>R.EIASPPHDLYR.S<br>K.DALHIDENTIER.I<br>K.NFVVNVVSR.L<br>R.IGEQNFHR.A<br>R.EKDYESLSQPSFFDR.F
281	PREDICTED: collagen alpha-2(VI) chain-like [Meleagris gallopavo] gi|326922323|ref|XP_003207399.1|	COL6A2	9	424	110229/5.55	1793.8700<br>1297.6545<br>1170.4908<br>1763.8688<br>1847.8290	1792.8996<br>1296.6462<br>1169.4811<br>1762.8890<br>1846.8373	50<br>61<br>71<br>67<br>63	K.LFAVAPSEDVYEQGLR.E<br>R.EIASPPHDLYR.S<br>K.HEAYAECYK.M<br>R.VGVVQYSHEGTFEAIK.L<br>R.EKDYESLSQPSFFDR.F
282	PREDICTED: collagen alpha-1(VI) chain [Meleagris gallopavo] XP_010711460.1	COL6A1	16	321	109009/5.63	1702.8954<br>1448.7774<br>1563.8560<br>1286.6707	1701.8475<br>1447.7307<br>1562.8053<br>1285.5939	67<br>39<br>36<br>45	K.WIAGGTHTGEALQFSK.E<br>R.FLEASKPAEDSVR.V<br>K.VLVFSDGNSQGITAR.A<br>K.TANYDVVYGER.H
283	PREDICTED: collagen alpha-1(VI) chain [Meleagris gallopavo] XP_010711460.1	COL6A1	16	164	109009/5.63	1448.8213<br>1563.9032	1447.7307<br>1562.8053	48<br>40	R.FLEASKPAEDSVR.V<br>K.VLVFSDGNSQGITAR.A
284	PREDICTED: collagen alpha-1(VI) chain [Meleagris gallopavo] XP_010711460.1	COL6A1	15	403	109009/5.63	1605.9093<br>1702.9021<br>1448.8018<br>1563.8728<br>1286.6825<br>1056.5978	1604.8423<br>1701.8475<br>1447.7307<br>1562.8053<br>1285.5939<br>1055.5400	49<br>65<br>52<br>31<br>54<br>31	K.VFSVAISPHHLDQR.L<br>K.WIAGGTHTGEALQFSK.E<br>R.FLEASKPAEDSVR.V<br>K.VLVFSDGNSQGITAR.A<br>K.TANYDVVYGER.H<br>R.GVFYQTVSR.K
285	PREDICTED: zona pellucida sperm-binding protein 3 [Meleagris gallopavo] XP_010715515.1	ZP3	30	746	48706/5.58	1590.7098<br>2456.2775<br>1870.0179<br>1666.8587<br>1366.6764<br>1144.5960	1589.6859<br>2455.2754<br>1868.9996<br>1665.8297<br>1365.6525<br>1143.5673	126<br>57<br>98<br>101<br>67<br>77	R.GDPSAWSYGAEAHSR.A<br>R.AVAGSHPVAVQCQEAQLVVTVHR.D<br>R.TLINYDPSPASNPVIIR.T<br>R.TNPAVIPIECHYPR.R<br>K.VTPADQGPDPQNK.A<br>R.NTWVPVEGSR.D

286	PREDICTED: zona pellucida sperm-binding protein 3 [Meleagris gallopavo] XP_010715515.1	ZP3	30	891	48706/5.58	1590.7150	1589.6859	126	R.GDPSAWSYGAEAHSR.A
						2456.3046	2455.2754	126	R.AVAGSHPVAVQCQEAQLVVTVHR.D
						1870.0343	1868.9996	158	R.TLINYDPSPASNPVIIR.T
						1666.8638	1665.8297	99	R.TNPAVIPIECHYPR.R
						1366.6774	1365.6525	67	K.VTPADQGPDPQNK.A
						1144.5973	1143.5673	86	R.NTWVPVEGSR.D
287	PREDICTED: zona pellucida sperm-binding protein 3 [Meleagris gallopavo] XP_010715515.1	ZP3	30	788	48706/5.58	1590.6416	1589.6859	119	R.GDPSAWSYGAEAHSR.A
						2456.1809	2455.2754	131	R.AVAGSHPVAVQCQEAQLVVTVHR.D
						1869.9426	1868.9996	125	R.TLINYDPSPASNPVIIR.T
						1666.7863	1665.8297	97	R.TNPAVIPIECHYPR.R
						1144.5355	1143.5673	91	R.NTWVPVEGSR.D
288	PREDICTED: cytochrome c oxidase subunit 5A, mitochondrial-like [Meleagris gallopavo]	COX5A	18	98	15982/5.21	1148.6516	1147.6098	34	R.RLNDFASAVR.I
						992.5543	991.5087	41	R.LNDFASAVR.I
289	PREDICTED: lactoylglutathione lyase [Meleagris gallopavo] XP 010705220.1	TPI1	39	287	20610/5.82	1298.7171	1297.6489	34	K.DFIFQQTMLR.V
						1314.7125	1313.6438	39	K.DFIFQQTMLR.V + Oxidation (M)
						1986.0230	1984.9490	84	R.KATLELTHNWGTENDEK.O
290	PREDICTED: calponin-3-like [Meleagris gallopavo] gi|326925093|ref|XP_003208756.1|	CNN3	52	292	34871/ 5.76	781.4047	780.3654	39	R.DIYDQK.H
						939.5244	938.4644	32	K.GMSVYGLGR.Q
						1560.6784	1559.6165	106	R.DYHGQYSDQGIDY.-
291	PREDICTED: zona pellucida sperm-binding protein 3 [Meleagris gallopavo] XP_010715515.1	ZP3	23	202	48706/5.58	1590.6510	1589.6859	85	R.GDPSAWSYGAEAHSR.A
						1869.9534	1868.9996	39	R.TLINYDPSPASNPVIIR.T
						1666.7733	1665.8297	48	R.TNPAVIPIECHYPR.R
292	PREDICTED: proteasome subunit alpha type-1 [Coturnix japonica] XP_015719573.1	PSMA1	22	190	29592/6.08	1778.8104	1777.8020	67	R.NQYDNDVTVWSPQGR.I
						2104.9652	2103.9782	66	R.KPALPADEPAEKAEEPMEH.- + Oxidation
293	mitochondrial translation elongation factor Tu [Gallus gallus] ARS01456.1	TUFM	21	227	47363/6.79	1838.1012	1836.9344	55	R.DRPHVNVGTIGHVDHGK.T
						1185.7235	1184.6149	45	R.AEAGDNVGALLR.G
						1172.6633	1171.5656	74	R.QPMVVEEGQR.F

294	PREDICTED: zona pellucida sperm-binding protein 3 [Meleagris gallopavo] XP_010715515.1	ZP3	28	500	48706/5.58	1590.6679<br>1869.9707<br>1666.8082<br>2111.7707	1589.6859<br>1868.9996<br>1665.8297<br>2110.8177	136<br>114<br>85<br>106	R.GDPSAWSYGAEAHSR.A<br>R.TLINYDPSPASNPVIIR.T<br>R.TNPAVIPIECHYPR.R<br>R.DVCSCCETGNCEPPALSR.R
295	PREDICTED: alcohol dehydrogenase [NADP(+)] [Meleagris gallopavo] XP_003208849.1	AKR1A1	24	223	40306/8.77	1299.6627<br>1497.6682<br>1529.6619	1298.6367<br>1496.6460<br>1528.6371	34<br>50<br>42	K.HHPEDVEPALR.K<br>R.YDYTDYKDTWK.A<br>R.DAGHPHYPFNDPY.-
296	PREDICTED: non-POU domain-containing octamer-binding protein isoform X2 [Meleagris gallopavo] XP_010713300.1	NONO	29	153	53144/9.00	1248.5884<br>1229.5775	1247.6081<br>1228.5949	33<br>33	R.FACHSASLTVR.N<br>R.GNPGGEFGPNKR.R
297	PREDICTED: polypyrimidine tract-binding protein 1-like [Meleagris gallopavo] gi|326934420|ref|XP_003213288.1|	PTBP1	11	212	56850/9.35	1461.7589<br>1897.8863<br>978.5568	1460.7412<br>1896.8822<br>977.5407	85<br>33<br>46	R.SQPIYIQFSNHK.E<br>K.LSLDGQNIYNACCTLR.I<br>K.HQTVQLPR.E
298	polypyrimidine tract-binding protein 1 isoform X3 [Columba livia] XP_021152653.1	PTBP1	10	239	64204/9.30	1461.7785<br>1897.9275<br>978.5790	1460.7412<br>1896.8822<br>977.5407	76<br>64<br>35	R.SQPIYIQFSNHK.E<br>K.LSLDGQNIYNACCTLR.I<br>K.HQTVQLPR.D
299	PREDICTED: phosphatidylinositol transfer protein alpha isoform [Meleagris gallopavo] XP_010720029.1	PITPNA	56	313	31443/5.80	2047.9452<br>1200.5353<br>1391.7309<br>934.4965	2046.9494<br>1199.5182<br>1390.7092<br>933.4821	90<br>34<br>44<br>30	K.NETGGGEGVEVLVNEPYER.D<br>K.AWNAYPYCR.T<br>K.NVEAIYIDIADR.S<br>R.LFTNFHR.Q
300	PREDICTED: zona pellucida sperm-binding protein 3 [Meleagris gallopavo] XP_010715515.1	ZP3	10	188	48706/5.58	1590.6859<br>1666.8900	1589.6859<br>1665.8297	99<br>48	R.GDPSAWSYGAEAHSR.A<br>R.TNPAVIPIECHYPR.R

301	PREDICTED: electron transfer flavoprotein subunit alpha, mitochondrial-like [Meleagris gallopavo]	ETFA	48	404	36666/6.25	820.4006<br>2040.9357<br>1750.9091	819.4239<br>2039.9656<br>1749.9301	35<br>115<br>91	K.SPNTFVR.T<br>R.TIYAGNVLCTVQCDEAVK.V<br>K.VTPPAPVGLSEWIEQK.L
302	stathmin [Gallus gallus] NP_001001858.1	STMN1	18	241	17072/6.18	1358.8133<br>1497.8453<br>1282.7173	1357.7354<br>1496.7875<br>1281.6605	76<br>73<br>67	R.ASGQAFELILGPR.S<br>R.SKEAAPEFPLSPPK.K<br>K.EAAPEFPLSPPK.K
303	PREDICTED: translocon-associated protein subunit delta [Gallus gallus] XP_015129111.1	SSR4	20	337	18551/5.67	1304.7254<br>1363.7042<br>1491.8080<br>1439.9011	1303.6309<br>1362.6092<br>1490.7041<br>1438.8045	64<br>85<br>79<br>65	R.YQVSWSLEHR.S<br>K.FFDEESYSALR.K<br>K.FFDEESYSALRK.A<br>R.IRPLFTVSVDHR.G
304	PREDICTED: epididymal secretory protein E1, partial [Meleagris gallopavo] XP_010710061.1		21	246	16360/7.11	1557.8099<br>1030.5988<br>1068.5664	1556.7253<br>1029.5277<br>1067.5036	122<br>30<br>74	K.DGSIQEVNVSPCPR.Q<br>K.SGIQCPIQK.G<br>K.GHSYSYLNK.L
305	PREDICTED: hypothetical protein LOC100549033 [Meleagris gallopavo] gi|326934995|ref|XP_003213567.1|		23	129	15326/6.14	1660.8240<br>1676.8150<br>1785.9510	1659.7709<br>1675.7658<br>1784.8846	59<br>41<br>87	K.QQSVLGPEMEQMQR.S<br>K.QQSVLGPEMEQMQR.S<br>K.GGEGIAAPIGPSSYPWAR.N
306	PREDICTED: actin-related protein 2/3 complex subunit 5, partial [Meleagris gallopavo] XP_003208573.2	ARPC5	26	138	12973/9.81	2131.1362	2130.0018	116	K.GFESPSDNSSAVLLQWHEK.A
307	PREDICTED: LOW QUALITY PROTEIN: zona pellucida sperm-binding protein 1 [Meleagris gallopavo] gi|326919872|ref|XP_003206201.1|	ZP1	19	618	96950/ 8.00	2190.9666<br>1803.6671<br>1419.7257<br>2523.3231<br>1073.5899<br>1358.7808	2189.9663<br>1802.6658<br>1418.7154<br>2522.3129<br>1072.5665<br>1357.7718	80<br>75<br>89<br>81<br>70<br>47	R.YHYDCGDFGMQLLAFPTR.G<br>R.DACLQAGCCFDDTDR.A<br>R.GLSAQPYNLDSVR.L<br>R.LVYENQLISTIDVQPGPHGSVTR.D<br>R.DSVYILHAR.C<br>K.VLRDPIYVEVR.L

308	PREDICTED: LOW QUALITY PROTEIN: zona pellucida sperm-binding protein 1 [Meleagris gallopavo] gi|326919872|ref|XP_00320620 [illegible]	ZP1	19	519	96950/ 8.00	1803.6644	1802.6658	84	R.DACLQAGCCFDDTDR.A
						1419.7218	1418.7154	97	R.GLSAQPYNLDSVR.L
						2523.3372	2522.3129	69	R.LVYENQLISTIDVQPGPHGSVTR.D
						1073.5806	1072.5665	44	R.DSVYILHAR.C
						1358.7777	1357.7718	35	K.VLRDPIYVEVR.L
						[illegible]	[illegible]	[illegible]	[illegible]
309	PREDICTED: zona pellucida sperm-binding protein 1 [Meleagris gallopavo]	ZP1	8	141	98702/7.62	1803.7341	1802.6658	68	R.DACLQAGCCFDDTDR.A
						1419.7754	1418.7154	61	R.GLSAQPYNLDSVR.L
310	PREDICTED: zona pellucida sperm-binding protein 1 [Meleagris gallopavo] XP_003206201.2	ZP1	8	317	98702/7.62	1803.8056	1802.6658	55	R.DACLQAGCCFDDTDR.A
						1419.8356	1418.7154	61	R.GLSAQPYNLDSVR.L
						1073.6648	1072.5665	40	R.DSVYILHAR.C
						2301.1904	2300.0485	92	R.IATDESYSSYHPDTDYPLVK.V
311	PREDICTED: zona pellucida sperm-binding protein 1 [Meleagris gallopavo] XP_003206201.2	ZP1	7	256	98702/7.62	1803.7678	1802.6658	34	R.DACLQAGCCFDDTDR.A
						1419.7968	1418.7154	59	R.GLSAQPYNLDSVR.L
						1073.6354	1072.5665	30	R.DSVYILHAR.C
						2301.1534	2300.0485	98	R.IATDESYSSYHPDTDYPLVK.V
312	PREDICTED: plastin-3-like [Meleagris gallopavo] gi|326924289|ref|XP_003208362.1|	PLS3	19	218	71267/5.51	2785.3732	2784.2032	79	K.EGICAIGGTSELSSEGTQHSYSEEEK.Y
						1458.8700	1457.7515	53	R.QFVTPADVVSGNPK.L
	Plastin-3, partial [Chelonia mydas] EMP39187.1	PLS3	18	191	68813/5.35	2785.3732	2784.2032	79	K.EGICAIGGTSELSSEGTQHSYSEEEK.Y
						1458.8700	1457.7515	53	R.QFVTPADVVSGNPK.L
313	PREDICTED: transketolase [Meleagris gallopavo] XP_010716778.1	TKT	23	206	64056/7.25	1446.7977	1445.7991	41	R.KIDSVLEGHPVPR.Q
						2060.0227	2059.0487	71	R.LGQSDPAPLQHHVEIYQK.R
						2543.1868	2542.2200	31	R.TSRPENPVIYNNNEDFHIGQAK.V
314	PREDICTED: zona pellucida sperm-binding protein 1 [Meleagris gallopavo] XP_003206201.2	ZP1	7	145	98702/7.62	1419.7957	1418.7154	58	R.GLSAQPYNLDSVR.L
						1073.6389	1072.5665	31	R.DSVYILHAR.C
						1358.8457	1357.7718	42	K.VLRDPIYVEVR.L
315	PREDICTED: zona pellucida sperm-binding protein 1 [Meleagris gallopavo] XP_003206201.2	ZP1	7	204	98702/7.62	1419.8206	1418.7154	58	R.GLSAQPYNLDSVR.L
						2301.1835	2300.0485	60	R.IATDESYSSYHPDTDYPLVK.V
316	PREDICTED: calcium-binding protein p22-like [Meleagris gallopavo]		56	151	21800/ 4.90	1885.9705	1884.9006	59	R.IINAFFSEGEDQVNFR.G
						790.4439	789.4286	32	K.LHFAFR.L

	gi|326920476|ref|XP_003206498.1|								
317	PREDICTED: phosphatidylethanolamine-binding protein 1-like, partial [Meleagris gallopavo] gi|326929994|ref|XP_003211138.1|	PEBP1	31	178	20406/9.44	1560.8224<br>1324.6955<br>1414.7185	1559.8195<br>1323.6863<br>1413.7034	35<br>46<br>46	K.LYTLVLTDPDAPSR.K<br>R.YVWLVYEQPK.Q<br>K.QLACNEPILSNR.S
318	PREDICTED: thioredoxin [Calidris pugnax] XP_014811930.1	TXN	20	170	12000/5.12	1395.7329<br>1221.5580	1394.6929<br>1220.5358	110<br>41	K.SVGSLSEFEAELK.S<br>K.CMPTFQFYK.S
319	PREDICTED: enhancer of rudimentary homolog [Meleagris gallopavo] gi|326920608|ref|XP_003206561.1|	ERH	41	290	12896/5.63	2054.8910<br>1105.6255<br>1328.6927	2053.8067<br>1104.5386<br>1327.6044	144<br>44<br>60	R.TYADYESVNECMEGVCK.M<br>K.MYEEHLKR.M<br>R.ADTQTYQPYNK.D
320	PREDICTED: glutaredoxin-related protein 5, mitochondrial-like [Meleagris gallopavo] gi|326921012|ref|XP_003206758.1|	GLRX5	20	161	10413/5.21	988.5679<br>1278.7351	987.4774<br>1277.6364	57<br>91	R.LHGVEDYR.A<br>R.AHDVLQDPDLR.Q
321	ATP synthase subunit e, mitochondrial [Gallus gallus] NP_001091003.1	ATP5ME	18	147	8334/7.93	1525.8044<br>1681.8997	1524.7460<br>1680.8471	86<br>46	R.YDYLKPIAEEER.R<br>R.YDYLKPIAEEERR.I
322	PREDICTED: peptidyl-prolyl cis-trans isomerase FKBP1A-like [Meleagris gallopavo] gi|326932031|ref|XP_003212125.1|	FKBP1A	13	86	11872/5.66	1533.7312	1532.7042	78	R.GWEEGVAQMSVGQR.A
323	PREDICTED: pterin-4-alpha-carbinolamine dehydratase-like, partial [Meleagris gallopavo] gi|326923467|ref|XP_003207957.1|	PCBD1	38	253	11916/6.06	1695.0286<br>1116.6188<br>1239.6832<br>829.4431	1693.9111<br>1115.5360<br>1238.5833<br>828.3952	41<br>79<br>39<br>40	R.LNAEEREQLLPNLR.A<br>R.AVGWNEVEGR.D<br>K.EFHFKDFNR.A<br>R.AFGFMTR.V

324	PREDICTED: galectin-1 [Meleagris gallopavo] XP_003202288.1	LGALS1	22	226	15211/6.58	1393.6974<br>1293.5911<br>995.5196	1392.6898<br>1292.5819<br>994.5124	103<br>48<br>32	K.DSTHLGLHFNPR.F<br>K.KMEEWGTEQR.E<br>R.ETVFPFQK.G
325	PREDICTED: 40S ribosomal protein S12-like [Meleagris gallopavo] gi|326915903|ref|XP_003204251.1|	RPS12	52	370	15967/8.82	1066.6346<br>2135.0869<br>1061.5892<br>970.5774<br>1585.8282	1065.5931<br>2133.9646<br>1060.5376<br>969.5356<br>1584.7671	42<br>44<br>36<br>33<br>84	K.TALIHDGLAR.G<br>R.QAHLCVLASNCDEPMYVK.L<br>K.LGEWVGLCK.I<br>K.IDREGKPR.K<br>K.ESQAKDVIEEYFK.C
326	PREDICTED: ATPase inhibitor, mitochondrial [Picoides pubescens] XP_009909816.1	ATP5IF1	30	244	8772/9.46	1438.8282<br>1966.1161	1437.6385<br>1964.9089	84<br>132	K.HHEEEIDHHQK.E<br>K.HHEEEIDHHQKEIER.L
327	glutathione S-transferase alpha class A2 [Meleagris gallopavo] gi|356640670|gb|AET31409.1|	GSTA2	27	293	25540/8.45	945.4869<br>1557.8365<br>932.5192	944.4644<br>1556.7947<br>931.4876	39<br>109<br>31	R.YFPVYEK.A<br>K.ALKDHGQDYLVGNK.L<br>K.FLQPGSQR.K
328	PREDICTED: glutathione S-transferase alpha class A1.3 isoform X2 [Meleagris gallopavo] XP_010706101.1		32	235	25379/8.76	1238.5459<br>979.5285	1237.5840<br>978.5538	67<br>46	K.DHGHDFLVGNK.L<br>K.FPLLQSFK.A
329	PREDICTED: superoxide dismutase [Mn], mitochondrial-like [Meleagris gallopavo] gi|326915675|ref|XP_003204139.1|	SOD2	17	183	26336/8.58	1032.5219<br>1687.9135	1031.4713<br>1686.8478	50<br>86	R.DFGSFANFK.E<br>K.AIWNVINWENVSSR.Y
330	glutathione S-transferase alpha class A1.2 [Meleagris gallopavo] gi|290874551|gb|ADD65348.1|	GSTA4	28	105	25269/8.75	1490.7860<br>979.6023	1489.7565<br>978.5538	45<br>42	R.WLLAAAGVEFEER.I<br>K.FPLLQSFK.A
331	glutathione S-transferase alpha class A4 [Meleagris gallopavo] gi|356640658|gb|AET31403.1|	GSTA4	11	109	26343/9.05	1257.5656	1256.5898	95	K.DHGQDFLVGNR.L
332	PREDICTED: proteasome activator complex subunit 3-like [Meleagris gallopavo] gi|326934185|ref|XP_003213174.1|	PSME3	48	272	28383/ 5.84	1155.6943<br>1668.8178<br>1078.5098	1154.6634<br>1667.8002<br>1077.4879	32<br>39<br>53	K.MWVQLLIPR.I<br>R.TVESEAASYLDQISR.Y<br>K.YPHVEDYR.R

333	PREDICTED: 3-mercaptopyruvate sulfurtransferase isoform X2 [Meleagris gallopavo] XP_010709815.1	MPST	27	224	33453/5.66	1876.9032<br>2489.2345<br>1590.7543	1875.8574<br>2488.1983<br>1589.7321	45<br>44<br>48	R.HIPGAVFFDIDQCSDR.T<br>K.LGVGNDSHVVVYDGSDQGLFSAPR.V<br>K.TYEDILDNLDSHR.F
334	PREDICTED: NADH dehydrogenase [ubiquinone] 1 beta subcomplex subunit 8, mitochondrial-like, partial [Meleagris gallopavo] gi|326923625|ref|XP_003208035.1|	NDUFB8	50	152	16408/5.67	2680.1913<br>1618.7110	2679.1475<br>1617.7212	38<br>68	R.VEDYQPYPDDGFGYGDYPMLPNK.S<br>R.DPWYQWDQPDLR.R
335	PREDICTED: retinol-binding protein 4-like [Meleagris gallopavo] gi|326923741|ref|XP_003208093.1|	RBP4	25	118	22856/6.30	1198.6618<br>2702.1578	1197.6182<br>2701.1140	45<br>51	K.YWGVASFLQK.G<br>K.GNDDHWVVDTDYDTYALHYSCR.E
336	PREDICTED: enoyl-CoA hydratase, mitochondrial [Meleagris gallopavo] XP_003207855.3	ECHS1	52	519	30017/8.55	1893.8731<br>2111.1736<br>1336.7083<br>1352.6991<br>1447.7738	1892.8152<br>2110.1171<br>1335.6526<br>1351.6476<br>1446.7143	103<br>143<br>60<br>44<br>75	K.TFQECYSGGFLAGWDR.V<br>K.AQFGQPEILLGTIPGAGGTQR.L<br>K.SLAMEMVLTGER.I<br>K.SLAMEMVLTGER.I + Oxidation (M)<br>R.LFYATFATDDRK.E
337	PREDICTED: eukaryotic translation initiation factor 4E-like [Meleagris gallopavo] gi|326919063|ref|XP_003205803.1|	EIF4E	33	224	26051/5.49	1887.9206<br>1503.7832	1886.9261<br>1502.7729	36<br>62	K.TEPAPTQEVASPEQYIK.H<br>K.IVIGYQSHADTATK.S
338	PREDICTED: platelet-activating factor acetylhydrolase IB subunit beta [Meleagris gallopavo] XP_003212802.1	PAFAH1B2	37	294	25636/5.66	2377.9428<br>958.4013<br>1140.6099	2376.9989<br>957.4239<br>1139.6299	99<br>42<br>34	-.MSHGDSNPAAVPHAAEDTQGDDR.W<br>R.WMSQHNR.F<br>R.LINTQQPQAK.V
339	PREDICTED: cytochrome b-c1 complex subunit Rieske, mitochondrial [Meleagris gallopavo] XP_003209829.2	UQCRFS1	20	214	27012/6.38	816.4752<br>1660.7676<br>1264.6213	815.5018<br>1659.7951<br>1263.6320	44<br>60<br>46	R.GKPLFVR.H<br>R.TQAEINQEAEVDVSK.L<br>K.LRDPQHDLDR.V
340	PREDICTED: thioredoxin-dependent peroxide reductase,	PRDX3	36	208	25810/7.60	1220.6849<br>3443.6948	1219.6673<br>3442.6140	56<br>94	K.HLSINDLPVGR.S<br>K.AFQYVETHGEVCPANWTPDSPTIKP-SPEASK.E

	mitochondrial, partial [Meleagris gallopavo] XP_010713240.1								
341	PREDICTED: proteasome subunit beta type-2 [Meleagris gallopavo] XP_010721633.2	PSMB2	24	203	33249/9.65	1281.6970	1280.6877	77	R.FVLNLTSFNAR.F
						1952.0243	1951.0415	58	R.FIDKDGIHEVDNVPLLK.A
342	10 kDa heat shock protein, mitochondrial [Gallus gallus] NP_990398.1	HSPE1	56	305	11133/8.68	1035.6277	1034.5913	39	R.KFLPLFDR.V
						1327.8027	1326.7620	44	K.VLQATVVAVGSGAR.G
						1076.6287	1075.5914	56	K.VLLPEYGGTK.I
						1573.8694	1572.8188	87	K.IVLEDKDYYLFR.D
343	PREDICTED: ATP synthase subunit d, mitochondrial-like [Meleagris gallopavo] gi|326931068|ref|XP_003211658.1|	ATP5F1D	67	198	14109/ 5.64	1249.6507	1248.6350	72	R.IAEYEQQLQK.L
						1726.8919	1725.8879	63	R.EKYPFWPHKPVADL.-
344	PREDICTED: vitellogenin-2-like [Meleagris gallopavo] XP_019473941.1	VTG2	6	162	194504/8.71	1576.9400	1575.8620	114	K.IVLQPVHTDANIEK.I
						1605.7752	1604.7617	32	R.GNAPDIPMQSYGSLR.Y
345	NADH dehydrogenase [ubiquinone] iron-sulfur protein 5 [Numida meleagris] XP_021230868.1	NDUFS5	56	160	12903/8.38	1979.8518	1978.8956	50	R.QSTTQPYGVAGACHAFER.E
						1528.6607	1527.6889	58	R.EWVECGHGLGQTR.A
346	PREDICTED: dihydropteridine reductase [Mesitornis unicolor] gi|704559850|ref|XP_010182378.1|	QDPR	21	175	25113/6.89	1631.6818	1630.8137	55	K.VDAILCVAGGWAGGSAK.A
						1645.7030	1644.8471	66	K.QSVWTSTISSHLATK.H
347	phosphoglycerate mutase 1 [Gallus gallus] NP_001026727.1	PGAM1	57	494	29051/7.03	1312.5937	1311.5956	93	R.HGESAWNLENR.F
						2070.8862	2069.8901	115	R.FCGWYDADLSPAGQQEAR.R
						2375.1154	2374.1304	48	R.SFDIPPPPMQSDHPFFSTISK.D
						1683.8940	1682.9032	73	R.ALPFWNEEIVPQIK.E
348	PREDICTED: BAG family molecular chaperone regulator 2 [Meleagris gallopavo] XP_019468781.1	BAG2	44	325	18359/5.32	1467.7582	1466.7477	66	R.NAQQQESLLHATK.M
						2105.0342	2104.0511	36	R.LLSLYGACTSDVPAGPIDQK.F
						1593.7956	1592.7868	70	K.FQSVVIGCAIEDQK.K
						1721.8908	1720.8818	70	K.FQSVVIGCAIEDQKK.I

349	calpain small subunit, partial [Gallus gallus] BAA22638.1	CAPNS1	38	243	24014/5.05	1906.9676	1905.9585	83	R.SGTIGVQELPGAFEAAGFR.L
						1236.7130	1235.7026	88	R.LPPELWGVLGR.R
350	PREDICTED: apolipoprotein A-I [Meleagris gallopavo] XP_010722021.1	APOA1	51	412	30593/5.58	1741.7869	1740.7856	50	R.SFWQHDDPQTPLDR.I
						1285.6193	1284.6172	35	K.LREDMAPYYK.E
						1351.7326	1350.7296	43	K.IRPFLDQFSTK.W
						1253.5727	1252.5724	74	K.WTEELEQYR.Q
						1057.6049	1056.5927	41	K.VVEQLSNLR.E
						1120.5786	1119.5747	33	K.MTPLVQEFR.E
351	thioredoxin domain-containing protein 17 [Numida meleagris] XP_021271377.1	TXNDC17	49	277	14474/5.46	1255.6750	1254.5993	88	R.GYGEFVQTAQR.Y
						1675.8387	1674.7494	95	R.SWCPDCVTAEPVVR.K
352	PREDICTED: fatty acid-binding protein, brain-like [Meleagris gallopavo] gi|326915985|ref|XP_003204292.1|	FABP7	64	281	13504/6.13	1523.5097	1522.6423	109	K.LGEEFDETTPDDR.N
						1251.4959	1250.6044	49	K.WDGKETNFVR.E
353	PREDICTED: hypothetical protein LOC100549033, partial [Meleagris gallopavo] gi|326934995|ref|XP_003213567.1|		30	150	15326/6.14	1660.7835	1659.7709	47	K.QQSVLGPEMEQMQR.S
						1785.9060	1784.8846	79	K.GGEGIAAPIGPSSYPWAR.N
354	PREDICTED: membrane-associated progesterone receptor component 1-like [Meleagris gallopavo] gi|326924618|ref|XP_003208522.1|	PGRMC1	31	166	11527/ 4.56	2553.2021	2552.1878	75	K.EALKDDYDDLSDLNATQQETLR.D
						1187.5464	1186.5295	68	R.DWESQFTFK.Y
355	PREDICTED: cytochrome b5 [Meleagris gallopavo] XP_003204966.1	CYB5A	63	472	15513/4.96	1205.5707	1204.5473	46	M.VGSSEAGGEAWR.G
						1705.8472	1704.8444	118	K.HNNSQSTWIIVHNR.I
						1497.7371	1496.7260	107	K.FLDEHPGGEEVLR.E
						2205.9208	2204.9207	49	R.EQAGGDATENFEDVGHSTDAR.A
						2038.0335	2037.0531	72	R.ALSETFIIGELHPDDRPK.L
356	PREDICTED: sorcin [Meleagris gallopavo] XP_010711000.2	SRI	27	182	20870/5.37	1237.5389	1236.5524	63	K.QHFVSFDSDR.S
						1504.6590	1503.6738	59	K.ITFDDYIACCVK.L

357	cytochrome b5 [Numida meleagris] XP_021263894.1	CYB5A	31	253	1080.6066	1079.5876	1079.5876	57	R.EAWLVIHGR.V
						2010.0029	2008.9854	93	R.FLEEHPGGEEVLLEQAGR.D
						1505.7112	1504.6946	49	K.QYYIGEIHPDDR.K
358	PREDICTED: 60S acidic ribosomal protein P2-like [Meleagris gallopavo] gi|326920193|ref|XP_003206359.1|	RPLP2	43	231	11835/4.42	1589.9076	1588.7944	31	K.KILDSVGIETDDER.L
						1461.8223	1460.6995	49	K.ILDSVGIETDDER.L
						1817.0459	1815.9214	34	K.ILDSVGIETDDERLNK.V
						2808.5160	2807.4123	69	K.LASMPAGGAVAVST-GGVSAAPAAGAAPAAAEEK.K
359	PREDICTED: chloride intracellular channel protein 2-like [Meleagris gallopavo] gi|326924230|ref|XP_003208334.1|	CLIC2	41	144	28476/5.39	1807.8108	1806.7778	41	K.AGLDGENIGNCPFCQR.L
						1605.7930	1604.7722	36	K.YKESFDVGSDIFAK.F
360	chloride intracellular channel protein 4 [Numida meleagris] XP_021231376.1	CLIC4	22	155	28675/5.44	1681.7643	1680.7162	65	K.AGSDGESIGNCPFSQR.L
						1371.7017	1370.6538	33	K.NSRPEANEGLER.G
361	PREDICTED: glutamate--cysteine ligase regulatory subunit, partial [Meleagris gallopavo] XP_010714156.1	GCLM	20	122	24394/5.20	1265.7083	1264.6776	30	K.LFIVGSNSSSVR.D
						1568.8375	1567.7995	65	K.QFDIQLLTHNDPK.G
362	Ran-specific GTPase-activating protein, partial [Nestor notabilis] KFQ44929.1	RANBP1	42	179	22752/5.19	1381.6730	1380.6296	61	K.TLEEDEEELFK.M
						1335.6511	1334.6143	39	R.FASENDLPEWK.E
						1034.5484	1033.5192	33	R.FLNAENAQK.F
363	PREDICTED: SH3 domain-binding glutamic acid-rich-like protein-like [Meleagris gallopavo] gi|326924407|ref|XP_003208419.1|	SH3BGRL	41	262	12787/5.07	1161.6553	1160.6414	38	R.RPASGNPLPPR.L
						1480.6857	1479.6670	92	R.YLGDYEAFFEAR.E
						1848.9555	1847.9417	87	R.ENNAVYAFLGLTAPPGSK.E
364	OVA, partial [Gallus gallus] AUD54558.1		20	136	43181/5.13	1687.9458	1686.8325	53	R.GGLEPINFQTAADQAR.E
						1860.0579	1858.9425	37	R.ELINSWVESQTDGIIR.N

365	PREDICTED: hypothetical protein LOC100549033, partial [Meleagris gallopavo] gi|326934995|ref|XP_003213567.1|		47	232	15326/6.14	1660.7397<br>1785.8595	1659.7709<br>1784.8846	68<br>126	K.QQSVLGPEMEQMQR.S<br>K.GGEGIAAPIGPSSYPWAR.N
366	PREDICTED: hypothetical protein LOC100549033, partial [Meleagris gallopavo] gi|326934995|ref|XP_003213567.1|		37	294	15326/ 6.14	1692.7969<br>970.5961<br>2913.4852<br>1785.9228	1691.7607<br>969.5495<br>2912.4304<br>1784.8846	38<br>43<br>100<br>80	K.QQSVLGPEMEQMQR.S 2 Oxidation (M)<br>R.SNPLLGLEK.R<br>R.QLEEVAQSDKGGEGIAAPIGPSSY-PWAR.N<br>K.GGEGIAAPIGPSSYPWAR.N
367	PREDICTED: apolipoprotein B-100 [Meleagris gallopavo] XP_010706280.1	APOB	6	185	525494/8.32	1519.6970<br>1416.6382	1518.6779<br>1415.6317	77<br>39	K.ALFDYFGYSHDGK.Q<br>K.EYSASANYDLQR.E
368	PREDICTED: apolipoprotein B-100 [Meleagris gallopavo] XP_010706280.1	APOB	8	309	525494/8.32	1519.6430<br>1416.6014	1518.6779<br>1415.6317	78<br>69	K.ALFDYFGYSHDGK.Q<br>K.EYSASANYDLQR.E
369	PREDICTED: apolipoprotein B-100 [Meleagris gallopavo] XP_010706280.1	APOB	3	144	525494/8.32	1519.7238<br>1416.6754	1518.6779<br>1415.6317	55<br>31	K.ALFDYFGYSHDGK.Q<br>K.EYSASANYDLQR.E
370	PREDICTED: heterogeneous nuclear ribonucleoprotein K isoform X3 [Gallus gallus] XP_004949259.1	HNRNPK	45	504	47467/5.64	1579.6742<br>1780.7662<br>1533.7567<br>1098.4290<br>1340.7714<br>1194.6739<br>1259.5516	1578.6984<br>1779.7911<br>1532.7875<br>1097.4448<br>1339.7962<br>1193.6921<br>1258.5677	46<br>81<br>70<br>42<br>52<br>71<br>34	K.RPAEDMEEEQAFK.R<br>R.TDYNASVSVPDSSGPER.I<br>K.IIPTLEEYQHYK.G<br>K.GSDFDCELR.L<br>K.IILDLISESPIK.G<br>R.NLPLPPPPPPR.G<br>K.IDEPLEGSEDR.I
371	PREDICTED: bleomycin hydrolase [Meleagris gallopavo] XP_003211808.1	bleomycin hydrolase	31	212	53449/5/64	1417.7794<br>1091.4875	1416.7613<br>1090.4679	44<br>58	K.TLYNNQPIDVLK.K<br>R.VENSWGEDR.G
372	PREDICTED: rho GDP-dissociation inhibitor 1-like [Meleagris gallopavo] gi|326930769|ref|XP_003211514.1|	ARHGDIA	34	378	23305/5.04	1917.9837<br>964.5172<br>1615.8190	1916.9327<br>963.4926<br>1614.7712	114<br>53<br>93	K.SIQEIQELDKDDESLR.K<br>K.YIQHTFR.K<br>K.IDKTEYMVGSYGPR.A

373	probable global transcription activator SNF2L2 isoform X7 [Strigops habroptila] XP_030327249.1	SMARCA2	17	286	36925/6.36	1762.7771<br>1416.7977<br>1324.6945	1761.7727<br>1415.7773<br>1323.6710	77<br>105<br>83	K.AIEDGNLEEMEEEVR.L<br>R.QLSEVFIQLPSR.K<br>K.ELPEYYELIR.K
374	PREDICTED: proteasome subunit alpha type-3-like [Meleagris gallopavo] gi|326921206|ref|XP_003206853.1|	PSMA3	39	187	31756/ 5.10	1095.5953<br>1380.7945	1094.5356<br>1379.7343	35<br>74	K.LYEEGSNKR.L<br>R.HVGMAVAGLLADAR.S
375	RecName: Full=Cathepsin B; AltName: Full=Cathepsin B1; Contains: RecName: Full=Cathepsin B light chain; Contains: RecName: Full=Cathepsin B heavy chain; Flags: Precursor P43233.1		23	281	38475/5.74	1324.5953<br>1880.9661<br>1824.8836	1323.5554<br>1879.9289<br>1823.8472	38<br>70<br>87	R.HCEPGYSPSYK.E<br>K.SGVYQHVSGEQVGGHAIR.I<br>R.GEDHCGIESEIVAGVPR.M
376	Hydroxyacyl-coenzyme A dehydrogenase, mitochondrial, partial [Anas platyrhynchos] EOB01155.1	HADH	32	193	31796/6.62	1551.7304<br>1018.5104	1550.7616<br>1017.5244	99<br>46	K.FADKPEAGAEFIEK.T<br>K.DTPGFIVNR.L
377	PREDICTED: 3,2-trans-enoyl-CoA isomerase, mitochondrial-like [Meleagris gallopavo] gi|326929302|ref|XP_003210806.1|		35	205	34322/8.93	1325.5806<br>1032.5158	1324.5836<br>1031.5222	66<br>45	K.STEHYAEFWR.A<br>R.AVQEMWLR.L
378	PREDICTED: phosphatidylethanolamine-binding protein 1 [Meleagris gallopavo] XP_003211138.2	PEBP1	58	519	20406/9.44	1560.8364<br>1457.7019<br>1977.9661<br>1324.6922<br>1414.7159	1559.8195<br>1456.6922<br>1976.9327<br>1323.6863<br>1413.7034	69<br>36<br>181<br>75<br>68	K.LYTLVLTDPDAPSR.K<br>R.EWHHFLVTNMK.G + Oxidation (M)<br>K.GNDVESGTVLSDYVGSGPPK.G<br>R.YVWLVYEQPK.Q<br>K.QLACNEPILSNR.S
379	PREDICTED: FAS-associated death domain protein [Meleagris gallopavo] XP_003206412.1	FADD	11	128	23246/6.23	1386.8307<br>1300.7838	1385.7337<br>1299.6783	58<br>54	K.VVIEVICENVGR.D<br>R.DLQEQLAQSLR.E

380	hypothetical protein ASZ78_002096 [Callipepla squamata] OXB64166.1 endoplasmic reticulum resident protein 29 [Meleagris gallopavo]	ERP29	24	278	28274/7.63	1340.7273	1339.7024	53	K.GSVPLDTITFYK.V
						1247.5745	1246.5506	74	K.FDTQYPYGEK.Q
						1703.8933	1702.8526	106	K.ILEQGEEFAANEVVR.I
381	PREDICTED: NADH dehydrogenase [ubiquinone] 1 beta subcomplex subunit 10 [Meleagris gallopavo] XP_003210738.1	NDUFB10	25	179	20748/6.22	988.4619	987.4450	30	K.SYYYHQK.F
						1311.7803	1310.7558	34	R.LVDQQIVEIVR.E
						1158.5787	1157.5578	55	R.YGDLGVHGNAR.T
382	PREDICTED: ovalbumin isoform X1 [Meleagris gallopavo] XP_010706723.1	SERPINB14	24	340	43274/5.18	1677.8007	1676.8118	104	R.GGLESINFQTAADQAR.G
						1537.7484	1536.7572	70	K.AFKDEDTQAIPFR.V
						1848.9033	1847.9166	94	K.ISQAVHAAYAEIYEAGR.E
383	PREDICTED: peptidyl-prolyl cis-trans isomerase B, partial [Fulmarus glacialis] XP_009572727.1	PPIB	53	396	18298/9.63	1378.7031	1377.7140	87	K.TVENFVALATGEK.G
						1286.5718	1285.5761	97	K.DFMIQGGDFTR.G
						1587.7398	1586.7365	38	K.SIYGDRFPDENFK.L
384	PREDICTED: ovalbumin isoform X1 [Meleagris gallopavo] XP_010706723.1	SERPINB14	23	207	43274/5.18	1677.8262	1676.8118	80	R.GGLESINFQTAADQAR.G
						1537.7670	1536.7572	54	K.AFKDEDTQAIPFR.V
						1848.9336	1847.9166	33	K.ISQAVHAAYAEIYEAGR.E
