# Supplementary material for: Transcriptome and Proteome Analysis Revealed Key Pathways Regulating Final Stage of Oocyte Maturation of the Turkey (Meleagris gallopavo)
Source: Int J Mol Sci. 2021 Sep 30;22(19):10589. doi: 10.3390/ijms221910589 (PMC8508634; doi:10.3390/ijms221910589)
Supplement: Supplementary file 1 [file ijms-22-10589-s001.zip › Table S4.pdf]

Table S4. Identification of protein spots visible after Western blot signal using anti-ubiquitin antibodies.

Spot no	Protein accession, description	Sequence coverage	Mascot score	Precursor mass observed	Precursor mass theoretical	Peptide score	Peptide sequence
1	zona pellucida sperm-binding protein 1 [Meleagris gallopavo] gi|733887584|ref|XP_003206201.2|	19	398	2190.9753	2189.9663	98	R.YHYDCGDFGMQLLAFPTR.G
				2206.9769	2205.9612	30	R.YHYDCGDFGMQLLAFPTR.G + Oxidation (M)
				1803.6707	1802.6658	45	R.DACLQAGCCFDDTDR.A
				2523.3312	2522.3129	50	R.LVYENQLISTIDVQPGPHGSVTR.D
				2349.2824	2348.2641	90	R.TQLVPVGPATLQLPFPSHYQR.F
2		17	436	2190.9886	2189.9663	82	R.YHYDCGDFGMQLLAFPTR.G
				1803.6845	1802.6658	88	R.DACLQAGCCFDDTDR.A
				2523.3531	2522.3129	100	R.LVYENQLISTIDVQPGPHGSVTR.D
				2349.2993	2348.2641	94	R.TQLVPVGPATLQLPFPSHYQR.F
3		20	397	2191.0169	2189.9663	72	R.YHYDCGDFGMQLLAFPTR.G
				1803.7186	1802.6658	88	R.DACLQAGCCFDDTDR.A
				2523.3871	2522.3129	85	R.LVYENQLISTIDVQPGPHGSVTR.D
				2349.3282	2348.2641	100	R.TQLVPVGPATLQLPFPSHYQR.F
4		16	311	2190.9154	2189.9663	63	R/YHYDCGDFGMQLLAFPTR.G
				1803.6084	1802.6658	89	R.DACLQAGCCFDDTDR.A
				2349.2246	2348.2641	73	R.TQLVPVGPATLQLPFPSHYQR.F
5		14	439	2190.9484	2189.9663	101	R.YHYDCGDFGMQLLAFPTR.G
				1803.6299	1802.6658	75	R.DACLQAGCCFDDTDR.A
				2523.3225	2522.3129	57	R.LVYENQLISTIDVQPGPHGSVTR.D
				2349.2609	2348.2641	95	R.TQLVPVGPATLQLPFPSHYQR.F
6		14	490	2190.9140	2189.9663	117	R.YHYDCGDFGMQLLAFPTR.G
				1803.6210	1802.6658	98	R.DACLQAGCCFDDTDR.A
				1358.7600	1357.7718	128	K.VLRDPIYVEVR.L
				2349.2310	2348.2641	84	R.TQLVPVGPATLQLPFPSHYQR.F
7		13	348	2190.9074	2189.9663	91	R.YHYDCGDFGMQLLAFPTR.G
				1803.6043	1802.6658	79	R.DACLQAGCCFDDTDR.A
				2349.2140	2348.2641	110	R.TQLVPVGPATLQLPFPSHYQR.F
