# Supplementary material for: Transcriptome and Proteome Analysis Revealed Key Pathways Regulating Final Stage of Oocyte Maturation of the Turkey (Meleagris gallopavo)
Source: Int J Mol Sci. 2021 Sep 30;22(19):10589. doi: 10.3390/ijms221910589 (PMC8508634; doi:10.3390/ijms221910589)
Supplement: Supplementary file 1 [file ijms-22-10589-s001.zip › Table S5.pdf]

Table S5. Primary antibodies used in this study.

Antibody	Host species	Vendor	Catalog no	Application(s)/dilution(s)
Anti-ubiquitin antibody	Rabbit	Sigma–Aldrich	U5379	IHC (1:100)<br>WB (1:100)
Anti-N-cadherin antibody	Mouse	Thermo Fisher	33-3900	IHC (1:50)
Anti-beta catenin antibody	Rabbit	Invitrogen	71-2700	IHC (1:150)
Anti-occludin antibody	Rabbit	Invitrogen	71-1500	IHC (1:50)
Anti-ZO-1/TJ-1 antibody	Rabbit	Invitrogen	61-7300	IHC (1:100)
Anti-connexin 43 antibody	Rabbit	Sigma–Aldrich	C6219	IHC (1:400)
