# Supplementary material for: Transcriptome and Proteome Analysis Revealed Key Pathways Regulating Final Stage of Oocyte Maturation of the Turkey (Meleagris gallopavo)
Source: Int J Mol Sci. 2021 Sep 30;22(19):10589. doi: 10.3390/ijms221910589 (PMC8508634; doi:10.3390/ijms221910589)
Supplement: Supplementary file 1 [file ijms-22-10589-s001.zip › Table S6.pdf]

Table S6. Primer–probe sequences used for real-time PCR.

Made to order TaqMan® Gene Expression Assays			
**Gene symbol**	**Gene name**	**Primer/probe**	**Sequence 5′→3′**
ZP1	*Zona pellucida sperm-binding protein 1*	Forward primer<br>Reverse primer<br>Probe	CCTCTTCCACTTCCCTGTCA<br>GCAGCTCAGTGGCATTGTAA<br>ACCGGTTGGTGTACGAGAAC
ZP2	*ZP domain-containing protein*	Forward primer<br>Reverse primer<br>Probe	TGTCGTTGATGGGTGTGAGT<br>GACGCTGCAGTGGAAGTACA<br>TGGAAGTGAAGGCTTTTGCT
ZP3	*Zona pellucida sperm-binding protein 3*	Forward primer<br>Reverse primer<br>Probe	GGCAGGAAACTCTGCAGTTC<br>CTCCAGTACCCTCCACAGGA<br>GTTCCTTCAACAAAGCCAGC
ZP4	*ZP4 protein*	Forward primer<br>Reverse primer<br>Probe	GTGACCCTGCCACCTGTTAT<br>GGATGCCACCAACTCATTCT<br>TGGCTGTGTCCCTGTAATGA
ZPAX	*ZPAX protein*	Forward primer<br>Reverse primer<br>Probe	TGCTTTGGCATGTCCTGTAG<br>GAAGGCCATTTCTTCAGCAG<br>GCTTGTTGAAGCTGGTGTGA
ZPD	*Zona pellucida protein D*	Forward primer<br>Reverse primer<br>Probe	CTCCGGTACTTCCTGCTGAG<br>AAGCTGAACTTGGCTGTGGT<br>TCCCCATGATGAGACAGTGA
ESR1	*Estrogen receptor 1*	Forward primer<br>Reverse primer<br>Probe	TAGAGGGCATGGTGGAAATC<br>TGTCCAGAACACGGTGGATA<br>TGATGAACCTTCAAGGGGAG
ESR2	*Estrogen receptor 2*	Forward primer<br>Reverse primer<br>Probe	GGTTCCGAGAGCTGAAACTG<br>TTGCAATAACCCACACCAGA<br>ATTCCAGCATGTTTCCCTTG
GPER1	*G_PROTEIN_RECEP_F1_2 domain-containing protein*	Forward primer<br>Reverse primer<br>Probe	CAGGTTGAGCTGTGGTCTCA<br>AAAGGCCGATGATCACAAAG<br>AACCCTGGGGTTTATCATCC
PRDX1	*Peroxiredoxin-1*	Forward primer<br>Reverse primer<br>Probe	ACCCCCTGGACTTCACTTTT<br>ATAGTGCCCAAACCACCTTG<br>GCCTGGGTCAACACTCCTAA
SOD1	*Superoxide dismutase 1*	Forward primer<br>Reverse primer<br>Probe	CACTTCAATCCTGAAGGCAAG<br>AATGATGCAGTGTGGTCCAG<br>TCGGCAATGTGACTGCTAAG
TXN	*Thioredoxin*	Forward primer<br>Reverse primer<br>Probe	TGGTTGATTTCTCTGCCACA<br>GTTGGCATGCACTTCACATC<br>TGTGGACCATGCAAAATGAT
GAPDH	*Glyceraldehyde-3-phosphate dehydrogenase*	Forward primer<br>Reverse primer<br>Probe	GGACACTTCAAGGGCACTGT<br>TACTCAGCACCTGCATCTGC<br>TCCAGGAGCGTGACCCCAGC
