# Supplementary figures and images for: Transcriptome and Proteome Analysis Revealed Key Pathways Regulating Final Stage of Oocyte Maturation of the Turkey (Meleagris gallopavo)
Source: Int J Mol Sci. 2021 Sep 30;22(19):10589. doi: 10.3390/ijms221910589 (PMC8508634; doi:10.3390/ijms221910589)

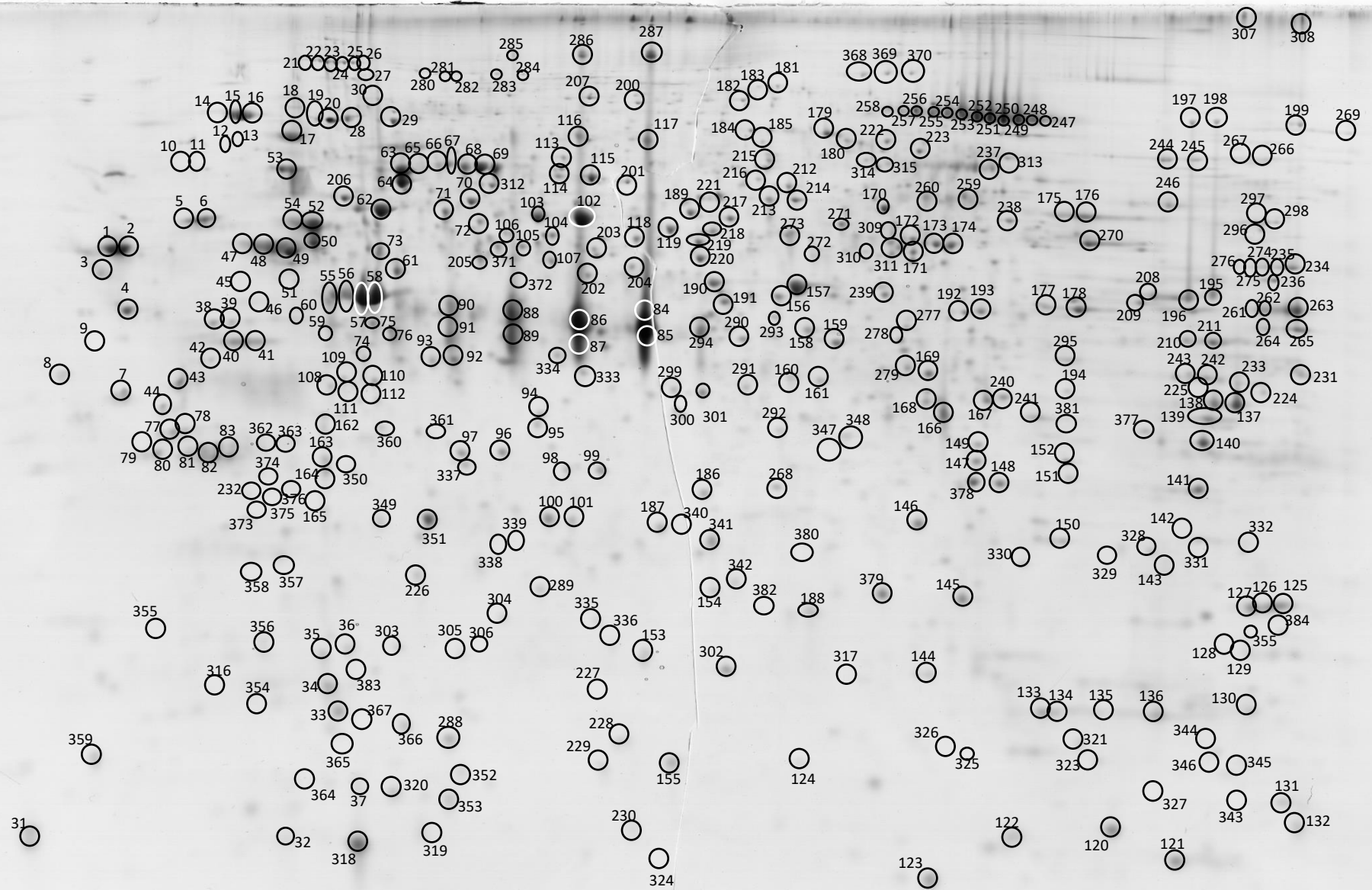

Supplement: Supplementary file 1 [file ijms-22-10589-s001.zip › Figure S1.pdf]

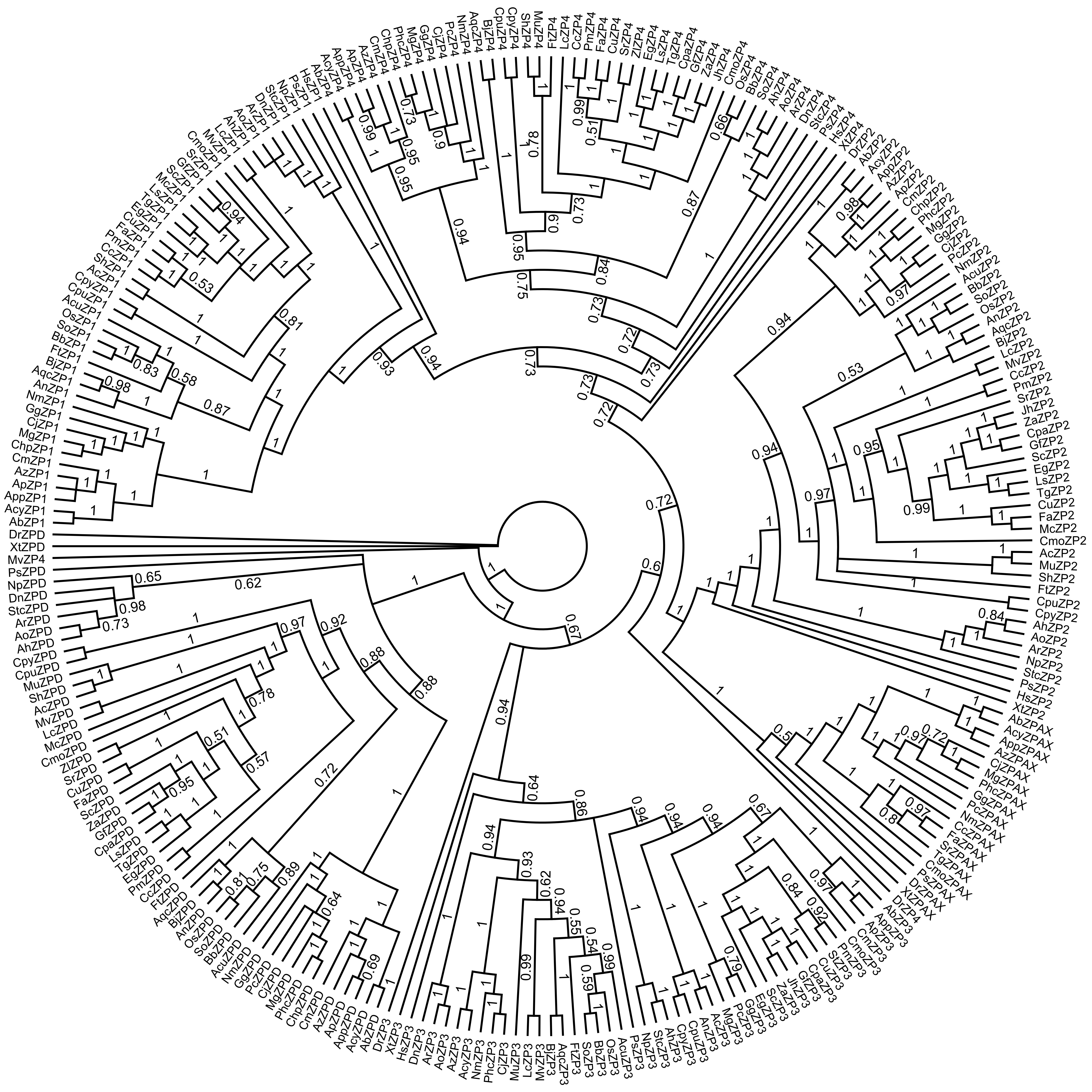

Supplement: Supplementary file 1 [file ijms-22-10589-s001.zip › Figure S2.jpg]

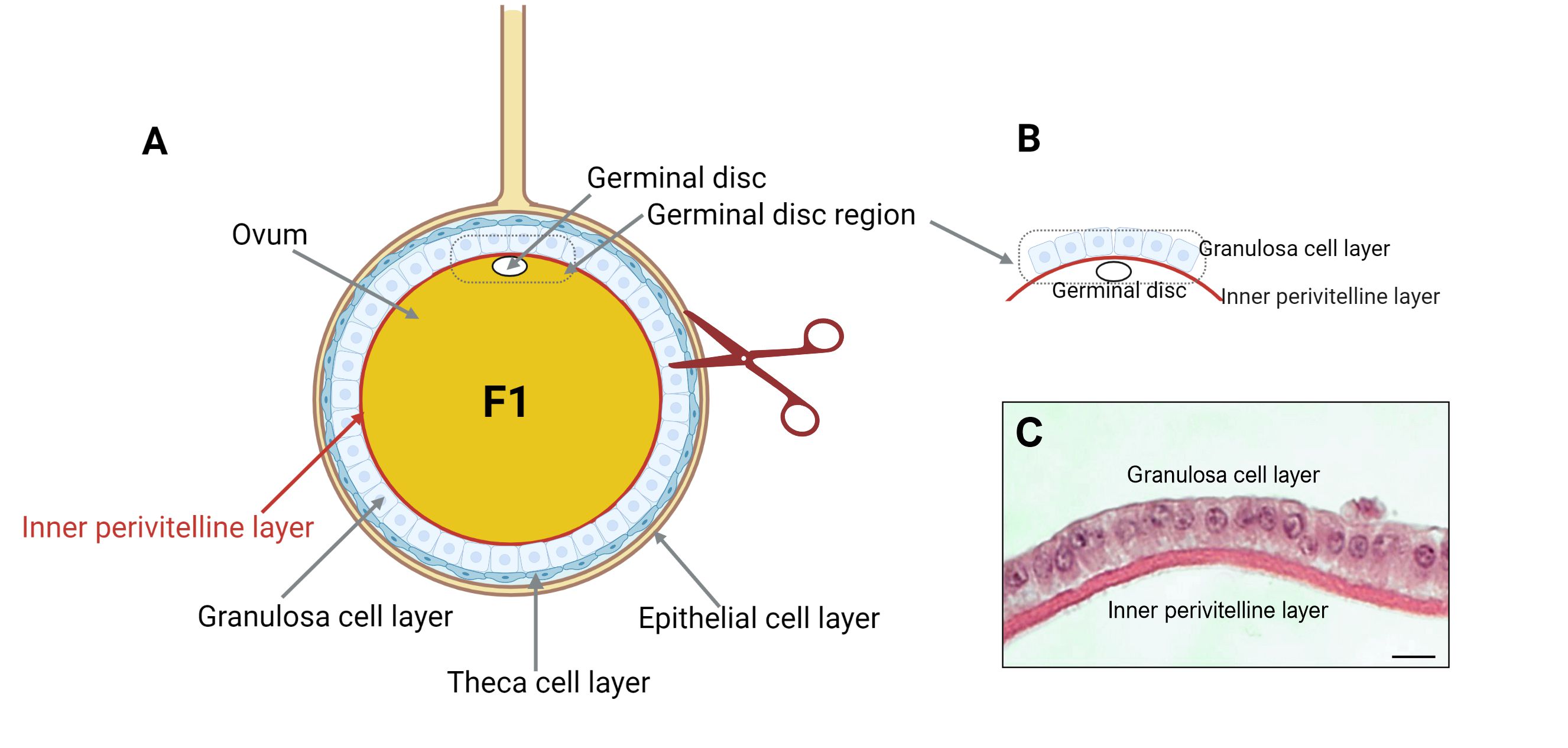

Supplement: Supplementary file 1 [file ijms-22-10589-s001.zip › Figure S3.jpg]
